# An (*R*)-Selective Transaminase From *Thermomyces stellatus*: Stabilizing the Tetrameric Form

**DOI:** 10.3389/fbioe.2020.00707

**Published:** 2020-07-22

**Authors:** Christian M. Heckmann, Louise J. Gourlay, Beatriz Dominguez, Francesca Paradisi

**Affiliations:** ^1^School of Chemistry, University of Nottingham, Nottingham, United Kingdom; ^2^Department of Biosciences, Università degli Studi di Milano, Milan, Italy; ^3^Johnson Matthey, Cambridge, United Kingdom; ^4^Department of Chemistry and Biochemistry, University of Bern, Bern, Switzerland

**Keywords:** biocatalysis, amino transferase, crystal structure, chiral amine, thermostability, quaternary structure, enzyme engineering, enzyme characterization

## Abstract

The identification and 3D structural characterization of a homolog of the (*R*)-selective transaminase (RTA) from *Aspergillus terreus* (*At*RTA), from the thermotolerant fungus *Thermomyces stellatus* (*Ts*RTA) is here reported. The thermostability of *Ts*RTA (40% retained activity after 7 days at 40°C) was initially attributed to its tetrameric form in solution, however subsequent studies of *At*RTA revealed it also exists predominantly as a tetramer yet, at 40°C, it is inactivated within 48 h. The engineering of a cysteine residue to promote disulfide bond formation across the dimer-dimer interface stabilized both enzymes, with *Ts*RTA_G205C retaining almost full activity after incubation at 50°C for 7 days. Thus, the role of this mutation was elucidated and the importance of stabilizing the tetramer for overall stability of RTAs is highlighted. *Ts*RTA accepts the common amine donors (*R*)-methylbenzylamine, isopropylamine, and d-alanine as well as aromatic and aliphatic ketones and aldehydes.

## Introduction

A green chemistry approach to minimize the environmental impact of synthetic processes entails the use of biocatalysts, as they often offer unmatched regio-, enantio-, and chemo-selectivity (Truppo, 2017). In addition, biocatalysts are bio-renewable and biodegradable (Sheldon and Woodley, 2018). One class of biocatalysts, at the forefront of industrial applications, are transaminases (TAs) (Fuchs et al., 2015; Kelly et al., 2018), pyridoxal-5′-phosphate (PLP) dependent enzymes catalyzing the shuttling of an amino group between an amine and a carbonyl group, producing chiral primary amines, a highly desired reaction in industry (Constable et al., 2007; Savile et al., 2010). Both (*S*)-selective transaminases (STAs) and (*R*)-selective transaminases (RTAs), belonging to separate fold types within the family of PLP-dependent enzymes, are known. RTAs share their fold type with branched chain amino transferases (BCATs) and d-amino acid transaminases (dATAs), which are significantly more abundant in nature and well-characterized, while RTAs remained elusive prior to Höhne et al. (2010) describing a consensus motif. Since then, several more RTAs have been reported, with just a few crystal structures solved. However the overall number of known RTAs (and their solved structures) is still significantly smaller compared to STAs (Guo and Berglund, 2017; Slabu et al., 2017).

The application of TAs (as well as other classes of enzymes) in chemical synthesis is often hindered by their insufficient stability (Bommarius and Paye, 2013). Key strategies to address this limitation include the sourcing of biocatalysts from extremophilic organisms (Littlechild et al., 2007), as well as enzyme engineering (Bommarius, 2015). To expand the toolbox of RTAs in order to include more inherently stable catalysts, the identification of a homolog of the RTA from *Aspergillus terreus* (*At*RTA) (Höhne et al., 2010; Łyskowski et al., 2014) from the thermotolerant fungus *Thermomyces stellatus* (*Ts*RTA) is here described together with its 2.2 Å crystal structure. An in-depth investigation of the quaternary structures of both RTAs is presented. RTAs are commonly accepted to exist exclusively as dimers, while both dimeric and tetrameric STAs have been reported (Börner et al., 2017). Finally, we describe the introduction of a mutation that stabilizes the quaternary structure, improving the thermostability of both enzymes.

## Materials and Methods

All reagents were purchased from Sigma Aldrich, Thermo Fisher, Alfa Aesar, Apollo Scientific, or Fluorochem and used without further purification. Restriction enzymes, polymerases, and ligases were purchased from New England Biolabs. MALDI-TOF MS was carried out using ground steel target plates on a Bruker ultraFlex III MALDI-TOF mass spectrometer. NMR spectra were obtained using a Bruker 400 MHz NMR spectrometer (Bruker AV3400HD). ESI-MS data were obtained on a Bruker MicroTOF spectrometer.

### Discovery of Novel RTA Sequences

Protein BLAST searches were performed with the sequences of several reported RTAs against the NCBI non-redundant protein sequence database (https://blast.ncbi.nlm.nih.gov/Blast.cgi) as well as the now defunct fungal genomics database (https://genome.fungalgenomics.ca/) of the Genozymes project. Candidate sequences were searched for sequences from extremophilic or extremotolerant organisms which were then inspected for the consensus sequence described by Höhne et al. (2010) Multiple sequence alignments against several reported RTAs were performed using MUSCLE (Madeira et al., 2019) to assess sequence identities.

### pCH93b

The QuikChange Lightning Multi Site-Directed Mutagenesis Kit from Agilent was used to introduce a TEV recognition sites it pET22b(+) and to insert a second NdeI restriction site to facilitate excision of the PelB sequence by digestion with NdeI for 2–3 h [30 μL reaction: CutSmart (10 x) buffer (3 μL), restriction enzyme (2 μL; 40 U), DNA (2 μg)]. Following gel purification, the backbone was then closed in a 16°C ligation reaction [20 μL reaction: 10x T4 ligase buffer (2 μL), vector-backbone (200 ng), T4 ligase (1 μL)] and transformed into *E. coli* XL10-Gold using electroporation (see Supporting Information for full plasmid map).

The following primers were used:

TEV recognition site insertion:

5′-GCTTGCGGCCGCAGAGAACCTCTATTTCCAAGGGCTCGAGCACCACC-3′

Introduction of second NdeI restriction site:

5′-GAATTAATTCCGATATCCATATGCATCGCCGGCTGGGCAGCG-3′

Mutations in red.

### Cloning

The codon-optimized synthetic gene of *Ts*RTA was purchased from GeneArt in the cloning vector pMA, flanked by BamHI and HindIII restriction sites. Restriction digests of pMAT-*Ts*RTA and pCH93b-*Cv*STA were set up in separate vials as follows: pMAT-*Ts*RTA (700 ng) or pCH93b-*Cv*STA (1,500 ng), CutSmart (10x) buffer (3 μL), BamHI HF (2 μL), HindIII HF (2 μL) and nuclease free water to 30 μL followed by incubation at 37°C for 30 min. The backbone pCH93b and the insert *Ts*RTA were purified from an agarose gel (1%, 150 mA, 75 V, 50 min) using the GeneJet gel purification kit (excised backbone and insert were combined prior to purification). DNA was eluted in 43 μL nuclease free water (45°C). 10x T4 ligase buffer (5 μL) as well as T4 ligase (2 μL) were added. The reaction was incubated at 16°C overnight. DNA was isolated by ethanol precipitation and transformed into electrocompetent *E. coli* XL10-gold (1750 V, 5.5 ms) and, following outgrowth in SOC medium (37°C, 180 rpm 1 h), plated onto selective LB-agar plates (ampicillin (amp) 100 μg/mL), and incubated at 37°C overnight. The presence of the insert and absence of mutations were verified by sequencing.

The synthetic gene of *At*RTA was kindly provided by Johnson Matthey. The gene was amplified (Q5 polymerase 2x Master Mix (12.5 μL), AtRTA_fwd (0.5 μM), AtRTA_rev (0.5 μM), template (ca 1–10 ng), final volume 25 μL. Cycling conditions: 98°C, 30 s; 30x (98°C, 10 s, 67°C 30 s, 72°C 30 s); 72°C 5 min, 4°C. The mix was purified (GeneJet PCR purification kit), digested (PCR product (1.4 μg) or pCH93b (2.4 μg) (23 μL), SacI HF (2 μL), HindIII HF (2 μL), CutSmart (10x) buffer (3 μL); incubation at 37°C for 90 min), gel purified as above (elution in 25 μL diluted kit elution buffer (25% *v/v* in nuclease free water, 65°C), and ligated (sample (25 μL), 10x T4 ligase buffer (3 μL), T4 ligase (2 μL), incubation at 25°C for 30 min, followed by 65°C for 10 min). Electroporation was then carried out as above, using 5 μL of the ligation mix.

AtRTA_fwd: 5′-ACAGATAGAGCTCCATGGCCAGCATGGACAAAG-3′

AtRTA_rev: 5′-ACAGATAAAGCTTATTACGCTCGTTATAGTCGATTTCAAACG-3′

### Mutagenesis

The mutants *At*RTA_G207C and *Ts*RTA_G205C were prepared using the QuikChange Lightning multi site-directed mutagenesis kit from Agilent, following the manufacturer protocol: 10 × QuikChange Lightning Multi reaction buffer (2.5 μL), pCH93b harboring the RTA gene (ca 100 ng), primer (0.4 μM), dNTP mix (1 μL), QuikSolution (1 μL), QuikChange Lightning Multi enzyme blend (1 μL), nuclease free water (to 25 μL). Cycling conditions: 95°C, 2 min; 30x (95°C, 20 s, 55°C 30 s, 65°C 3.5 min); 65°C 5 min, 4°C. DpnI (1 μL) was added and the mix incubated at 37°C for 1 h. A portion (4 μL) of the mix were then transformed into the provided XL10-gold cells and, following outgrowth in SOC medium (37°C, 180 rpm 1 h), plated onto selective LB-agar plates (amp 100 μg/mL), and incubated at 37°C overnight. The mutations were then verified by in-house Sanger sequencing.

The following primers were used:

AtRTA_G207C: 5′-CAACCTATCCATTTTTAACGGATTGTGATGCCCATTTAACGG-3′;

TsRTA_G205C: 5′-ATCCGTTTCTGACCGATTGTGATGCCAATCTGAC-3′.

Mutated codon in red.

### Expression and Purification

pCH93b containing the synthetic gene was transformed into BL21 STAR (DE3) *E. coli* cells. TB-medium (amp 100 μg/mL) (300 mL) supplemented with lactose (5 g/L) were inoculated with a single colony and incubated at 37°C, with shaking (180 rpm) for 4 h followed by 25°C with shaking (180 rpm) for 20 h. Cells were harvested by centrifugation (4,500 g, 15 min, 4°C) and stored at −20°C either as pellets or in resuspension buffer. Proteins were purified by immobilized metal affinity chromatography (IMAC) as described by Cerioli et al. (2015). Enzyme concentration was estimated by the absorbance at 280 nm (non-denatured protein), using predicted extinction coefficients (*Ts*RTA: 40493.38 Da, 53860 M^−1^ cm^−1^, *At*RTA: 39860.55 Da, 50880 M^−1^ cm^−1^; https://web.expasy.org/protparam/).

### Size-Exclusion Chromatography

Size-exclusion chromatography (SEC) was carried on a Superdex 200 10/300 GL column (GE Healthcare), using a mobile phase of potassium phosphate buffer (50 mM), sodium chloride (100 mM), PLP (0.1 mM), pH 8. Injection volume: 100 μL, flow rate 0.75 mL/min. Samples were prepared in potassium phosphate buffer [50 mM), PLP (0.1 mM), pH 8 (with or without sodium chloride (100 mM)], to a final protein concentration of ca 2 mg/mL. Samples were either injected directly after preparation or incubated at ambient temperatures for varying amounts of time to follow the interconversion of different quaternary states. A calibration curve was generated using the Sigma Aldrich Gel Filtration Markers Kit for Protein Molecular Weights 12,000–200,000 Da (MWGF200) (Figure S4 insert).

### Stability, Activity, and Kinetic Assays

Activity assays were based on the method by Schätzle et al. (2009) as applied in Cerioli et al. (2015), with (*R*)-methylbenzylamine (RMBA) in place of SMBA in UV-free 96-well plates using the EPOCH 2 plate reader at 30°C unless otherwise stated (pathlength 0.84 cm, calculated according to $\frac{A_{977}-A_{900}}{0.18}$), ε = 12.6 mM^−1^ cm^−1^).

#### Temperature, pH, and Co-solvent Stability Assays

The purified enzyme in potassium phosphate buffer (50 mM, pH 8, 0.1 mM PLP) was diluted to a final conc. of 0.30–0.38 mg/mL with either phosphate buffer (temperature stability assays), universal buffer adjusted to the desired pH (pH stability assays) (Cerioli et al., 2015), or phosphate buffer containing 10 or 20% [(*v/v*), final concentration] co-solvents (co-solvent stability assays). Aliquots (30 μL) were stored at 35–60°C for the temperature stability assays, 4°C for pH stability, and 25°C for the co-solvent stability assays. For each time point, one aliquot was briefly centrifuged in a microfuge to ensure the complete collection of the sample at the bottom of the tube, and incubated on ice for ca. 10 min. The standard activity assay described above was performed in triplicate on each sample, using 3 μL of the enzyme sample per reaction. The sample was then discarded.

#### Temperature, pH, and Co-solvent Activity Assays

Modified activity assays were performed as follows: for temperature activity assays, 3 μL of enzyme solution, stored on ice at the appropriate dilution (0.07–0.09 mg/mL), and 300 μL of assay buffer, pre-heated to the desired temperature, were combined. The assay was performed in a 96-well plate in a pre-heated plate reader. For pH activity assays, assay buffer was prepared in universal buffer (Davies, 1959) at the desired pH. For the co-solvent activity assays, assay buffer containing the desired co-solvent was prepared. To initiate the assay, 3 μL of enzyme solution, stored on ice at the appropriate dilution (0.37 mg/mL), and 300 μL of assay buffer, were combined and the assay performed as usual.

#### Kinetic Studies

Activity assays were performed by either fixing the pyruvate concentration at 10 mM (1% (*v/v*) DMSO) while the RMBA concentration was varied (0.005 to 10 mM) or with the RMBA concentration fixed at 2.5 mM (0.25% (*v/v*) DMSO) and the pyruvate concentration varied (0.005 to 10 mM), using purified enzyme at the appropriate dilution (0.0030–0.0038 mg/mL, final concentration in the assay). Assays at each concentration were performed in triplicate. Kinetic parameters *k*_*cat*_, *K*_*m*_, and *K*_*i*_ where obtained by fitting a substrate inhibition curve $\left(v=\frac{k_{cat}}{1+\frac{K_{m}}{\left[S\right]}+\frac{\left[S\right]}{K_{i}}}\right)$ using GraphPad Prism (Copeland, 2000).

### Substrate Scope Analysis

Reactions were set up containing RMBA (10 mM) [IPA (50 mM) for acetophenone] and a carbonyl acceptor (10 mM), or benzaldehyde (10 mM) [pyruvate (10 mM) for MBA] and an amino donor [10 mM (20 mM for racemates)], purified *Ts*RTA (wild-type and mutant, 0.5 mg/mL), PLP (0.1 mM), DMSO (5 or 10% *v/v*) in potassium phosphate buffer (50 mM pH 8), in a final volume of either 600 μL or 1 mL. Reactions were incubated at 37°C, 180 rpm in triplicate. Samples (100 μL) were quenched in with 900 μL acetonitrile and aq. HCl (0.2%) (1:1 *v/v*) and analyzed by reverse-phase HPLC. Enantiopreference was determined by chiral GC-FID or chiral reverse-phase HPLC (see below). For the intensification studies, reactions were set up analogously but with an increasing amount of RMBA and phenoxyacetone, and the ratio of enzyme to the substrate was kept constant (0.1 mg/mL for a 10 mM reaction or 0.025 mol%). Reactions were quenched analogously but with proportionally increasing dilution factors.

### MALDI-TOF MS

Protein samples (20 μL) in potassium phosphate buffer [50 mM; PLP (0.1 mM), pH 8] were diluted with an equal volume 1% TFA. For preparation of reduced samples, TCEP (10 μL of a 200 mM stock) was added to the sample, followed by incubation at 70°C for 15 min. Samples were then desalted using C4 ZipTip® pipette tips (Merck Millipore) as follows: The sample was bound to the tip under saturating conditions, the resin washed (5% methanol, 0.1% TFA in water; 20 × 10 μL) and the protein eluted in 5 μL elution buffer (80% acetonitrile, 0.1% TFA in water). The desalted samples (2 μL) were mixed with 2 μL of a solution of sinapic acid (20 mg/mL; in elution buffer) of which 1 μL was spotted onto a ground steel target plate, dried, and coated with 1 μL of the sinapic acid solution. The spots were then analyzed using a Bruker ultraFlex III MALDI-TOF mass spectrometer (laser amplitude 60%). External calibration relative to HSA (66,440 kDa).

### Reverse-Phase HPLC Analysis of Conversions

Samples were analyzed using a ThermoFisher Ultimate 3000 Reverse-phase HPLC (diode array detector) on a Waters XBridge C18 column (3.5 μm, 2.1 × 150 mm) with the following method: A: 0.1% TFA in water, B: 0.1% TFA in acetonitrile. Gradient: 0 min 95% A 5% B; 1 min 95% A 5% B; 5 min 5% A 95% B; 5.10 min 0% A 100% B; 6.60 min 0% A 100% B; 7 min 95% A 5% B; 10 min 95% A 5% B. Injection volume 2 μL, at 45°C with a flow rate of 0.8 mL/min. Retention times in min: acetophenone (3.85), MBA (2.11), benzaldehyde (3.60), benzylamine (1.06), phenoxyacetone (3.90), 1-phenoxypropan-2-amine (3.10). Conversions were calculated from a calibration curve of authentic standards, following the production of product(s).

### Enantiopreference

Samples were basified by adding 1:10 sodium hydroxide (5 M), saturated with sodium chloride, and extracted into 2 × 500 μL ethyl acetate. Extracted samples were derivatized with 20 μL each triethylamine and acetic anhydride and analyzed by GC-FID: Thermo Scientific™ Trace™ 1310 GC equipped with an Agilent CHIRASIL-DEX CB (25 m × 0.25 mm × 0.25 μm) column: 0 min 40°C, 1 min 40°C, 4 min 100°C, 5 min 100°C, 15 min 110°C, 16 min 110°C, 17.8 min 200°C, 22.8 min 200°C. Injector temperature 230°C, split ratio 1:10, continuous flow 1.7 mL/min, FID temperature 250°C. Helium was used as carrier gas. Retention times in min: (*S*)-*o*-fluoro-α-methylbenzylamine (11.2), (*R*)-*o*-fluoro-α-methylbenzylamine (11.1), (*S*)-1-aminoindan (14.9), (*R*)-1-aminoindan (15.0), (*S*)-4-phenylbutan-2-amine (14.7), (*R*)-4-phenylbutan-2-amine (14.8), (*S*)-hexan-2-amine (8.0), (*R*)-hexan-2-amine (8.1), (*S*)-tetrahydrothiophene-3-amine (13.1), (*R*)-tetrahydrothiophene-3-amine (13.0), (*S*)-α-Ethylbenzylamine (13.3), (*R*)-α-Ethylbenzylamine (13.4), SMBA (12.3), RMBA (12.6), (*S*)-1-phenoxypropan-2-amine (14.7), (*R*)-1-phenoxypropan-2-amine (14.8).

Alternatively, samples were derivatized with FMOC-Cl [100 μL sample, 200 μL borate buffer (100 mM, pH 9), 400 μL FMOC-Cl (15 mM in acetonitrile)], diluted 5-fold with acetonitrile and aq. HCl (0.2%) (1:1 *v/v*) and analyzed by reverse-phase HPLC (diode array detector) on a Phenomenex Lux Cellulose-2 chiral column (5 μm, 44.6 × 250 mm) with the following isocratic methods: A: 0.1% TFA in water, B: 0.1% TFA in acetonitrile. Tetrahydrofuran-3-amine and butan-2-amine: 40% A 60% B, serine: 55% A 45% B. Injection volume 2–20 μL, at ambient temperature with a flow rate of 1 mL/min. Retention times in min: (*S*)-tetrahydrofuran-3-amine (11.3), (*R*)-tetrahydrofuran-3-amine (12.3), (*S*)-butan-2-amine (13.0), (*R*)-butan-2-amine (11.7), l-serine (6.0), d-serine (6.2; shoulder: acetophenone (6.4)].

Retention times of each enantiomer were identified by comparing to commercially available samples (either a racemate and one enantiomer, or both enantiomers), except for phenoxypropan-2-amine, where (*S*)-phenoxypropan-2-amine synthesized using the *Halomonas elongata* transaminase (Cerioli et al., 2015) was used (see below), tetrahydrothiophene-3-amine, where a commercial racemate and (*S*)-tetrahydrothiophene-3-amine synthesized from l-methioninol according to the procedure by Pan et al. (2011) was used and serine, where commercial l-serine was used.

#### (S)-Tetrahydrothiophene-3-Amine

[α]_D_−32.2, c 1, acetone [lit. (Dehmlow and Westerheide, 1992) −37.77], ^1^H-NMR (400 mHz, CDCl_3_) δ 1.51 (3 H, br s, NH_2_ + H_2_O), 1.81–1.90 (1 H, m, SCH_2_C*H*_*a*_H_b_), 1.94–2.04 (1 H, m, SCH_2_CH_a_*H*_*b*_), 2.59 (1 H, ddd (*J* 10.7, 4.4, 0.9 Hz), SC*H*_*a*_H_b_CHN), 2.84–2.96 (2 H, m, SC*H*_2_CH_2_), 2.98 (1 H, dd (*J* 10.6, 5.1 Hz), SCH_a_*H*_*b*_CHNH_2_), 3.71 (1 H, p (*J* 4.9 Hz), C*H*NH_2_), ^13^C-NMR (100 mHz, CDCl_3_) δ 28.4 (S*C*H_2_CH_2_), 38.7 (SCH_2_*C*H_2_), 40.0 (S*C*H_2_ CHNH_2_), 55.9 (CHNH_2_); in agreement with lit. (Dehmlow and Westerheide, 1992; Pan et al., 2011) ESI-MS (*m/z*): [M+H]: calc. 104.0528, found 104.0538.

#### Synthesis of (S)-1-Phenoxypropan-2-Amine

Phenoxyacetone (274 μL, 2 mmol), isopropylamine (IPA) [5 mmol, from a pH adjusted 2 M stock in potassium phosphate buffer (50 mM, pH8)], PLP [2 mL of a 10 mM stock in potassium phosphate buffer (50 mM, pH8)], and DMSO (2 mL) were diluted with potassium phosphate buffer (50 mM, pH8) to a final volume of 20 mL. Lyophilized HEwT cfe (25.5 mg) (Cerioli et al., 2015) was added and the mixture incubated with gentle agitation at 25–30°C. After 24 h, an additional 25 mg of HEwT and after another 24 h a further 50 mg were added. The reaction was incubated for another 72 h after which the reaction was basified with 3 mL NaOH (5 M) and extracted with 3 × 20 mL EtOAc. The combined organic extracts were dried with MgSO_4_, filtered, and concentrated *in vacuo* to ca 2 mL. Methanolic HCl (80 μL, 3M, prepared from acetyl chloride and methanol) was added and the sample concentrated *in vacuo*. The resulting oil was recrystalized from EtOAc to give the HCl salt of (*S*)-1-phenoxypropan-2-amine as white needles (290.3 mg, 77% yield, >99.5% *ee*). [α]_D_ 33.8, c 2, methanol (lit. Koszelewski et al., 2008 −28.1 for the (*R*)-enantiomer), ^1^H-NMR (400 mHz, DMSO-*d*_6_) δ 1.29 (3 H, t (*J* 6.7 Hz), Me), 3.58 (1 H, pd (*J* 6.8, 4.0 Hz), C*H*NH_3_), 3.99 (1 H, dd (*J* 10. 2, 7.0 Hz), C*H*_*a*_H_b_), 4.12 (1 H, dd (*J* 10.2, 4.0 Hz), CH_a_*H*_*b*_), 6.95–7.02 (3 H, m, *o, p*-Ar-H), 7.32 (2 H, dd (*J* 8.8, 7.0 Hz), *m*-Ar-H), 8.22 (3 H, br s, NH_3_), ^13^C-NMR (100 mHz, DMSO-*d*_6_) δ 15.0 (Me), 46.0 (CHNH_3_), 68.5 (CH_2_), 114.6 (*o*-C), 121.2 (*p*-C), 129.5 (*m*-C), 157.8 (Ar-C-O); in agreement with lit. (Knutsen et al., 1999; Franchini et al., 2003) ESI-MS (*m/z*): [M+H]: calc. 152.1070, found 152. 7078.

### TsRTA Crystallization and Data Collection

Crystallization trials of *Ts*RTA (10 mg/ml; 20 mM Tris-HCl pH 8.0; 0.1 mM PLP) were carried out using an Orxy 4 crystallization robot (Douglas Instruments) and flat-bottomed, Greiner CrystalQuick 96 well sitting drop plates (Greiner Bio-one). *Ts*RTA microcrystals grown over 2–3 days at 20°C in a 500 nl drop, containing 30% protein and PACT screen (Molecular Dimensions) condition G3 (0.2 M sodium iodide, 0.1 M Bis-Tris Propane pH 7.5, 20% *(w/v)* PEG 3350), were used to prepare a seed stock. Seeds were prepared using the Seed Bead Kit (Hampton Research), crushing the crystals in 50 μl well solution by vortexing for 2 min. 0.15 μl seed stock was used to seed a second PACT screen, preparing 0.8 μl drops at diverse protein concentrations (31.25, 50, and 68.75%). For microseeding, the protein concentration was halved to 5 mg/ml. Crystals were harvested from PACT condition C12 (0.01 M zinc chloride, 0.1 M HEPES pH 7.0, 20% *(w/v)* PEG 6K) from a drop containing 68.75% protein. For cryoprotection, crystals were soaked in a solution comprising 18.75% *(v/v)* PACT condition G3, 20% *(w/v)* PEG6K, 0.1M HEPES pH 7.0, 0.002 M ZnCl and 28% *(w/v)* ethylene glycol.

X-ray diffraction data were collected on a single TsRTA crystal at 2.2 Å resolution on the XDR2 beamline at the ELETTRA synchrotron facility (Trieste, Italy). Two TsRTA chains (Chains A and B) were present in the asymmetric unit, with an estimated Matthew's coefficient of 2.7 Å^3^/Da (54.4 % solvent content). Data reduction was carried out using Mosflm and assigned to the body-centered monoclinic space group I_1_2_1_ using POINTLESS and scaled with AIMLESS (Evans, 2011; Powell et al., 2017). Molecular replacement was carried out using MOLREP and chain A of the omega transaminase from *Aspergillus terreus* (*At*RTA; PDB entry 4ce5; 82% sequence identity over 321 residues) as a search model (Vagin and Teplyakov, 1997; Łyskowski et al., 2014). All programs are available under the CCP4 suite (Winn et al., 2011). The structure was manually built and refined to convergence using coot and phenix.refine and structure geometry was validated by Molprobity in the PHENIX platform (Table S1) (Davis et al., 2007).

For data collection and refinement parameters see Table S1. Atomic coordinates and structure factors are available for download from the RCSB Protein Data Bank (www.rcsb.org) under accession code 6XWB.

## Results and Discussion

### Expression and Initial Characterization of TsRTA

A putative RTA from the thermotolerant fungus *Thermomyces stellatus* (*Ts*RTA) with 81% identity to *At*RTA (Łyskowski et al., 2014) was identified from a protein BLAST search (Figure S1). *Ts*RTA was readily expressed at 25°C and, following Ni-IMAC, the enzyme was obtained in high yields (800 mg_enzyme_/L_culture_) and was judged to be pure by SDS-PAGE (Figure S2). The specific activity with pyruvate and RMBA was determined to be 2.5 U/mg (*At*RTA: 3 U/mg). The catalytic efficiency (*k*_*cat*_/*K*_*m*_) was comparable to *At*RTA for RMBA and ca. 2-fold lower for pyruvate (Table 1). Substrate inhibition was observed in both *Ts*RTA and *At*RTA for both pyruvate and RMBA, with higher inhibition from the latter (Figure S3).

**Table 1:** Kinetic parameters for wild-type (wt) and mutant *Ts*RTA and *At*RTA.

		***K_**m**_* (mM)**	***k_**cat**_* (s^**−1**^)**	***K_**i**_* (mM)**	***k_**cat**_* /*K_**m**_* (s^**−1**^ mM^**−1**^)**
*Ts*RTA_wt	RMBA	0.13 ± 0.01	1.82 ± 0.03	15 ± 1	13.8 ± 0.6
	Pyruvate	0.57 ± 0.03	2.21 ± 0.04	39 ± 5	3.9 ± 0.1
*Ts*RTA_G205C	RMBA	0.12 ± 0.01	1.35 ± 0.03	20 ± 2	11.0 ± 0.6
	Pyruvate	0.42 ± 0.01	1.33 ± 0.02	130 ± 30	3.2 ± 0.1
*At*RTA_wt	RMBA	0.17 ± 0.01	2.24 ± 0.04	19 ± 1	13.3 ± 0.5
	Pyruvate	0.23 ± 0.01	2.25 ± 0.02	52 ± 4	10.0 ± 0.2
*At*RTA_G207C	RMBA	0.30 ± 0.02	1.96 ± 0.05	19 ± 2	6.4 ± 0.3
	Pyruvate	0.19 ± 0.01	1.71 ± 0.03	60 ± 10	9.2 ± 0.5

*Parameters were obtained by fitting a substrate inhibition curve (using GraphPad Prism) to the data obtained when either RMBA or pyruvate concentrations were varied. Reaction velocities at each concentration were measured in triplicate. Standard errors (SE) are quoted, accounting for covariance*.

The resting stability and activity of *Ts*RTA were then investigated under varying conditions. *Ts*RTA was stable in universal buffer between pH 5-9 for at least 14 days at 4°C (Figure S4a), and most active between pH 8-9, as is commonly observed for RTAs (Schätzle et al., 2011) (Figure S4b; it should be noted the overall specific activity in universal buffer Davies, 1959 was lowered by ca. 20-fold). *Ts*RTA was stable in the presence of co-solvents such as 20% (*v/v*) methanol, ethanol, and DMSO, which are commonly used co-solvents in biotransformations, with no loss of activity after 1 week (Figure S4d). Stability decreased with increasing chain length of alcohols, as well as THF, acetonitrile and to a lesser extent DMF. The activity in the presence of co-solvents followed the same trends as the stability (Figure S4c).

**Figure 1:**
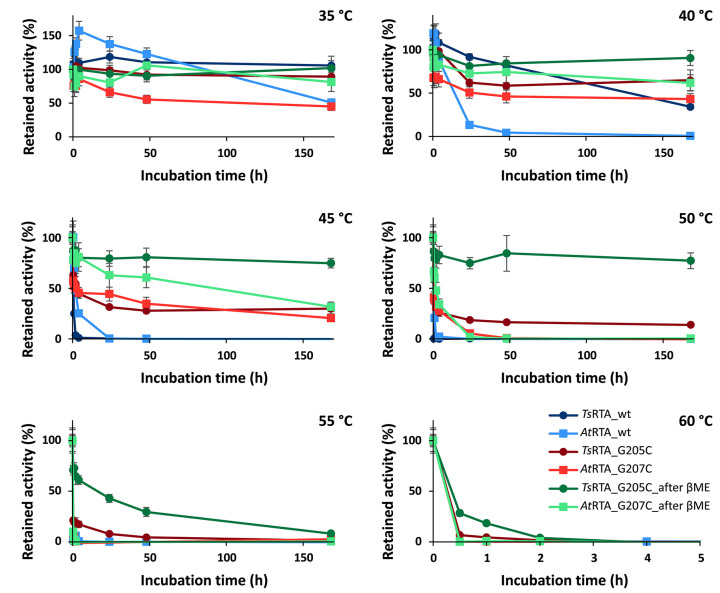
Thermal resting stability profiles of wild-type *Ts*RTA and *At*RTA, as well as mutants both before and after equilibration in the presence of β-mercaptoethenol. Retained activity after incubation at 35–60°C for 30 min−7 d (pH 8). Activity expressed relative to the activity of each enzyme at t = 0. Error bars represent standard errors (*n* = 3).

**Figure 2:**
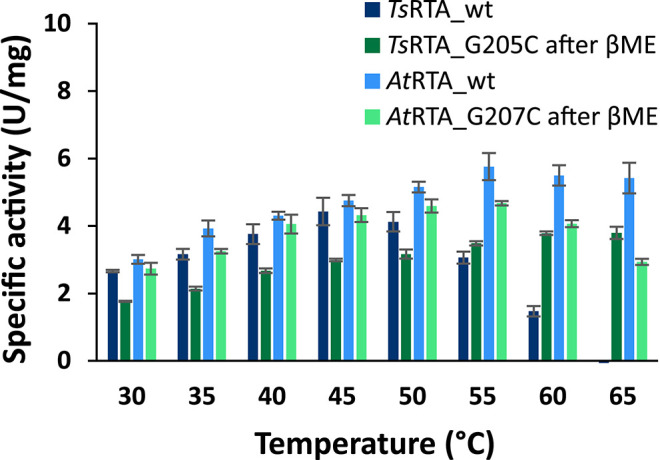
Temperature–activity relationship of wild-type and mutant *Ts*RTA and *At*RTA: specific activity at 30–65°C. Error bars represent standard errors (*n* = 3).

To assess the extent to which the thermotolerant origin of *Ts*RTA impacts its stability, thermostability assays were carried out in parallel with both *Ts*RTA and *At*RTA. *Ts*RTA retained 40% activity when incubated at 40°C for 7 days, whereas *At*RTA lost almost 90% activity within 24 h. However, at 45°C, *Ts*RTA almost completely lost its activity within 2 h, while *At*RTA retained ca. 25% activity after 4h (Figure 1). Indeed, during temperature activity assays *At*RTA showed a higher optimum temperature compared to *Ts*RTA (Figure 2), which showed loss of activity over the 10 min activity assay from 50°C onward.

SEC unexpectedly revealed that 90% of *Ts*RTA in solution exists as a tetramer, with only 10% adopting the dimeric form (Figure S5). Other highly similar RTAs, including *At*RTA (4ce5, 81% identity) (Łyskowski et al., 2014), and RTAs from *Nectria haematococca* (4cmd, 78% identity) (Sayer et al., 2014), *Exophiala xenobiotica* (6fte, 70% identity) (Telzerow et al., 2019), and *Aspergillus fumigatus* (4uug, 73% identity) (Thomsen et al., 2014) are reported as dimers based on their crystal packing. Two thermostable BCATs, belonging to the same fold type IV as RTAs, with hexameric structures have recently been reported by Isupov et al. (2019). In addition, increased operational stability of tetrameric STAs compared to dimeric STAs has been reported (Börner et al., 2017). Thus, the improved thermostability of *Ts*RTA was initially attributed to its tetrameric nature (Littlechild et al., 2007).

### 3D Structural Analysis and Relation to Thermostability

Electron density was well-defined for residues 1–319 in both polypeptide chains (A and B) present in the asymmetric unit, but it was absent for *Ts*RTA residues 320–334, in addition to the C-terminal His-tag, due to flexibility in this region. Both polypeptide chains exhibit high structural similarity, with an RMSD of 0.24 Å for 319 aligned Cα atoms, as calculated using the CCP4i program SUPERPOSE (Krissinel and Henrick, 2004). Regions of poor electron density were observed for several side chains from residues 126–132 that are located in a loop region and solvent exposed. There was an area of positive density that appeared to be continuous with the sidechain of D65 (Chain A), observed in the difference map (mFo-DFc), that could not be identified or modeled.

**Figure 3:**
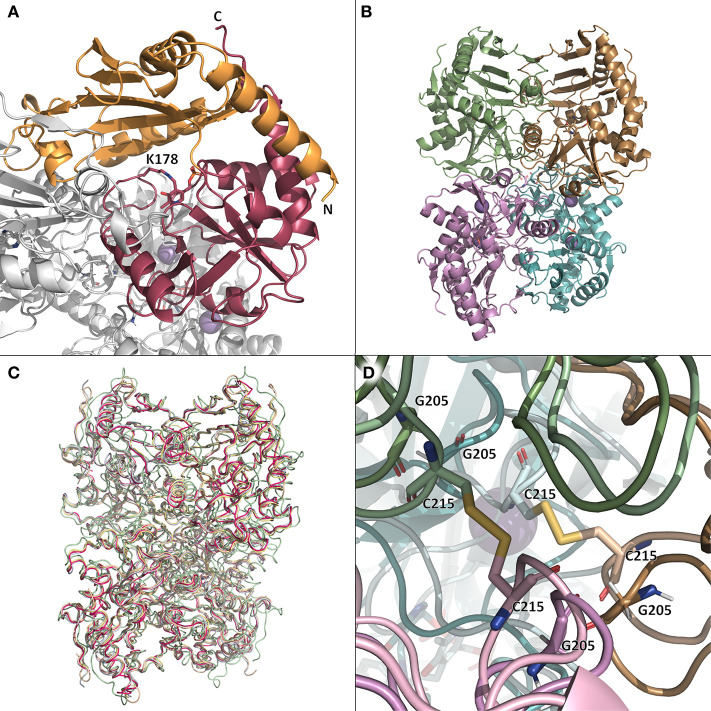
**(A)** The monomer (chain B) of *Ts*RTA in the context of the overall quaternary structure. The two sub-domains are colored orange and pink. The co-factor PLP (attached to K178) is shown as sticks. **(B)** The tetramer of *Ts*RTA obtained by applying the symmetry operation (-x, y, -z) to the asymmetric unit dimer. **(C)** superimposition of the crystallographic tetramers of *Ts*RTA (pink), *At*RTA (4ce5, wheat), ATA-117-Rd11 (pale green), *N. haematococca* RTA (4cmd, blue-white), and *A. fumigatus* RTA (4uug, pale yellow). **(D)** Zoom view of the two disulfide bridges (C215) that extend across the dimer-dimer interface of 5fr9 (lighter colors) and the equivalent glycine residues (G205) of *Ts*RTA (darker colors). This figure was generated with open source PyMOL 2.1.0.

The *Ts*RTA monomer adopts the classical aminotransferase class IV fold (InterPro: IPR001544; Pfam PF01063) with a significant content of both α-helices and β-strands (3 β-sheets) and can be observed to be sub-divided into two smaller sub-domains (see Figure 3A and Figures S6, S7). A Profunc search (http://www.ebi.ac.uk/thornton-srv/databases/profunc/) (Laskowski et al., 2005), as expected, identified *At*RTA as the top structural homolog (RMSD of 0.67 Å over 318 C-alpha matched pairs).

The active site pocket houses a single PLP molecule, covalently bound to each *Ts*RTA chain via an imino bond with the conserved active site lysine residue (K178) and additional hydrogen bonds (2.6–3.0 Å) formed between the phosphate group of PLP and side chain atoms from residues R77, E211, T237, and T273 (conserved in *At*RTA); the phosphate group is further stabilized by hydrogen bonds with R77 and N219 via two conserved water molecules (Figure S8) (Łyskowski et al., 2014). E211 forms a hydrogen bond (2.8 Å) with the pyridine nitrogen; for other class IV members, it is suggested that the role of this residue is to maintain the pyridine ring in its protonated form, thus stabilizing the carbanion reaction intermediate. The pyridine ring is further stabilized by hydrophobic residues with a conserved leucine residue (L233) and the backbone of a conserved phenylalanine (F215).

An analysis of the dimer interface formed with the PDBePISA server (https://www.ebi.ac.uk/pdbe/pisa/) revealed an interaction surface area of 2143.5 Å^2^ mediated by 57 interacting residues (Complexation Significance Score (CSS) of 1.0). Contact surface interactions comprise 30 hydrogen bonds and 10 salt bridges, with an estimated solvation energy gain upon interface formation of ΔG^i^ = −22.6 kcal mol^−1^.

A tetrameric structure of ATA-117-Rd11 (40% sequence identity) has been deposited in the PDB as 5fr9 (Cuetos et al., 2016) This structure contains two disulfide bridges at the tetrameric interface (Figures 3C,D), while glycine residues are found in all of the above-mentioned RTAs, as well as wild-type ATA-117 (Figure 3D and Figure S9). Despite the fact that a tetramer interface was not predicted by PDBePISA, yet in agreement with our experimental findings, a *Ts*RTA tetramer was generated by applying the symmetry operation (-x, y, -z) to the asymmetric dimer, which superimposed well with the tetramer of ATA-117-Rd11 (Figure 3B). Additionally, for all of the above mentioned structures of RTAs, except for *E. xenobiotica*, a similar crystallographic-tetramer can be obtained (Figure 3C), yet, interface analysis with PDBePISA, only predicted *At*RTA to form a tetramer. Gel-filtration chromatography of *At*RTA then revealed a composition of 97% tetramer, 2% dimer and 1% monomer (Figure S5). Analysis of known tetrameric STAs (Börner et al., 2017) (PDB entries 4b9b, 4atp, 3n5m, 3a8u, and 5lh9) showed PDBePISA only predicted the first three to be stable tetramers in solution. Thus, the analysis of the “dimer of dimers” structure by PDBePISA was deemed unreliable. Attempts to disrupt the tetrameric structure by simple variation of the ionic strength of the buffer (0–1,200 mM NaCl) were made but no effect was observed.

*Ts*RTA_G205C and *At*RTA_G207C variants, produced to mimic the equivalent tetramer-bridging G215C mutation of ATA-117-Rd11, exhibited a disrupted quaternary structure immediately following purification: a composition of ca. 55% tetramer, 42% dimer, and 2% monomer and 28% tetramer, 19% dimer, and 52% monomer were observed for *Ts*RTA_G205C and *At*RTA_G207C, respectively (Figure S5), as judged by SEC. This can be attributed to an enhanced energy barrier for tetramer formation due to increased steric hinderance from the cysteines. Indeed, incubation at room temperature with gentle agitation showed slow reconstitution of the tetrameric form, but it tapered off over time (Figures S10, S11), possibly due to incorrect disulfide bond formation within the dimer. Incubation at 4°C in the presence of β-mercaptoethanol (10 mM) for 2 days, followed by dialysis to remove the reducing agent and an overnight incubation with aeration (to form the correct disulfide bond), on the other hand, resulted in 89% tetramer and 11% dimer, and 89% tetramer, 5% dimer, and 6% monomer for *Ts*RTA_G205C and *At*RTA_G207C, respectively (Figure S12). The presence of a disulfide bond bridging two subunits was confirmed by MALDI-TOF MS (Figure S13). For *At*RTA_G207C, a 65% increase in activity was observed following β-mercaptoethanol pre-treatment. This matches the increase in the relative amount of dimer and tetramer from 57 to 94%. Thus, the increase in activity is due to the inactive monomer being converted into the active dimeric and tetrameric forms. For *Ts*RTA_G205C, no such increase was observed implying that both the dimeric and tetrameric forms have similar activities. Both enzymes, displayed only marginally lower specific activities over their respective wild-type forms (1.8 and 2.7 U/mg vs. 2.5 and 3 U/mg for *Ts*RTA_G205C and *At*RTA_G207C, respectively) due to a decreased *k*_*cat*_. Inhibition from pyruvate was no longer detectable. The equivalent G207C mutation in *At*RTA largely had the same effect, but, in addition, it increased the *K*_*m*_ of RMBA, lowering the catalytic efficiency further. Any effect on substrate inhibition was less pronounced and statistically insignificant (Table 1).

The slow formation of the quaternary structure provided an opportunity to probe the thermostability of *Ts*RTA_G205C and *At*RTA_G207C with different proportions of the tetrameric form. Both mutants showed increased thermostability compared to the corresponding wild-type. However, the enzyme samples with a lower proportion of tetramer (In this case, *Ts*RTA_G205C: 75% tetramer, *At*RTA_G207C: 55% tetramer) showed a drop in activity within 30 min, followed by a more stable profile. Samples with 90% tetramer showed a much less pronounced activity loss (Figure 1). All samples showed a very similar profile, offset by the initial drop, thus supporting that the tetramer, stabilized by disulfide bonds, has improved thermostability over the dimer. *Ts*RTA_G205C showed higher thermostability than *At*RTA_G207C, and the switch in thermostability observed for the wild-type enzymes between 40°C and 45°C was absent in case of the mutants. The temperature optimum was shifted for *Ts*RTA_G205C showing considerable activity at 60°C and 65°C but not for *At*RTA_G207C which followed a similar profile to the wild-type (Figure 2).

These data suggest two different mechanisms by which these enzymes may lose their activity at elevated temperatures. The first, proceeding via dissociation of the tetramer followed by unfolding of the dimer, and the second being the direct unfolding of the tetramer. The first mechanism presumably is eliminated by the disulfide bond and it implies that *Ts*RTA is inherently more stable toward the second mechanism. This could explain why, at 40°C, wild-type *Ts*RTA is more stable than *At*RTA. Yet, at 45°C, *Ts*RTA might more rapidly dissociate into the dimeric form than *At*RTA, thus being less stable at higher temperatures (a weaker dimer-dimer interaction is consistent with the higher proportion of dimer observed for *Ts*RTA at equilibrium).

### Substrate Scope

To further characterize the newly identified *Ts*RTA, the scope toward carbonyl substrates was examined (Table 2). Aldehydes such as benzaldehyde, cinnamaldehyde, and vanillin were accepted, but only traces of conversion were observed with phenylacetaldehyde. Pyruvate was an excellent substrate. α-Ketoglutarate, the natural ketone acceptor for branched-chain aminotransferases (Höhne et al., 2010), was not a substrate, as well as the bulky 2,2-dimethylhexan-3-one. However, traces of conversion were observed when the propyl group was shortened to a methyl group. Butanone gave low levels of conversion. Extending the chain-length to hexan-2-one increased conversion. In both cases only the (*R*)-enantiomer was detected, showing excellent discrimination between the methyl and ethyl group in butanone in particular. Cyclohexanone on the other hand gave only low conversions. The heterocyclic ketones tetrahydrofuran-3-one and tetrahydrothiophene-3-one gave higher conversions, yet poor enantioselectivity. While the expected (*R*)-3-aminotetrahydrothiophene was produced, (*S*)-3-aminotetrahydrofuran was preferred, in both cases with ca. 20% *ee*. This is most likely due to different electronic interactions of the negatively polarized oxygen compared to the more neutral and bulkier sulfur atom. Coincidentally, this revealed that the enantiomer produced by the *Halomonas elongata* STA had been mis-assigned as (*S*) (Planchestainer et al., 2019), and it is the unexpected (*R*)-enantiomer that is produced preferentially in that case. β-Hydroxy-pyruvate reached its final conversion of ca. 50% after just 30 min, probably due to thermodynamic limitations. Acetophenone (with 5 eq. of isopropylamine) gave only traces of conversion, similarly to 1-indanone and propiophenone. *o*-Fluoroacetophenone and phenoxyacetone gave good conversions. Substituting the oxygen of the latter with a methylene (4-phenylbutanone) resulted in moderate conversion. In all cases only the (*R*)-enantiomer was detected. To verify that *Ts*RTA_G205C has a similar substrate scope, biotransformations with 5 representative substrates which showed lower conversions with the wild-type, were carried out with the mutant variant: vanillin, hexan-2-one, *o*-fluoroacetophenone, 4-phenylbutanone, and phenoxyacetone. In all cases higher conversions were achieved after 24 h, while conversions after 30 min remained similar to the wild-type. This indicates that the higher resting stability of *Ts*RTA_G205C also translates into improved operational stability, increasing the number of turnovers of the mutant. As expected, the mutant maintained excellent enantioselectivity.

**Table 2:** Carbonyl substrate scope (10 mM scale) of wild-type *Ts*RTA, unless stated otherwise^a^.

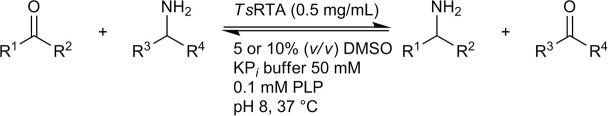				
**Substrate**		**Conversion (%)**		**Product** ***ee*** **(%)**
		**After 30 min**	**After 24 h**	
Benzaldehyde	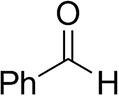	79 ± 1	89 ± 2	
Phenylacetaldehyde	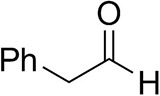	1.5 ± 0.8	2.5 ± 0.8	
Cinnamaldehyde	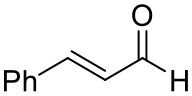	38 ± 1	60 ± 1	
Vanillin	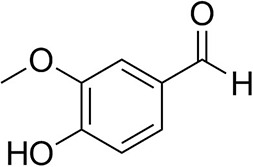	16 ± 1 12 ± 1^d^	72 ± 3 93 ± 3^d^	
Butanone	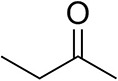	<0.5	12 ± 1	>99.5 (*R*)
Hexan-2-one^b^	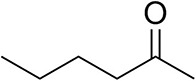	30 ± 1 30 ± 1^d^	57 ± 1 70 ± 2^d^	>99.5 (*R*)
Pinacolone	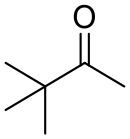	<0.5	1.8 ± 0.8	n.d.
2,2-Dimethylhexan-3-one	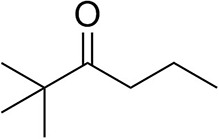	<0.5	<0.5	n.d.
Cyclohexanone	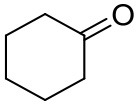	<0.5	7.7 ± 0.7	
Tetrahydrothiophene-3-one^b^	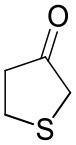	5.5 ± 0.7	32 ± 1	21 ± 1 (*R*)
Tetrahydrofuran-3-one^b^	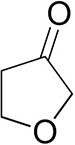	1.0 ± 0.8	25 ± 1	22 ± 1 (*S*)
Pyruvate	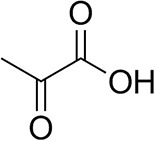	75 ± 1	95 ± 1	n.d.
β-Hydroxy-pyruvate	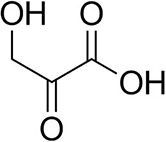	52 ± 1	46 ± 1	>99.5 (*R*)
α-Ketoglutarate	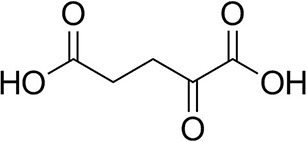	<0.5	<0.5	n.d.
Acetophenone^b^^,^^c^	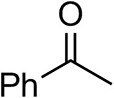	<0.5	4 ± 3	traces (*R*)
*o*-Fluoroacetophenone^b^	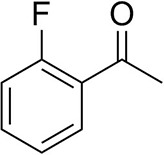	34 ± 1 35 ± 2^d^	70 ± 1 80 ± 3^d^	>99.5 (*R*)
1-Indanone^b^	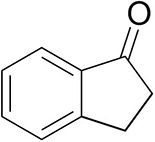	1.8 ± 0.8	3.6 ± 0.8	traces (*R*)
Propiophenone^b^	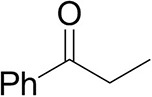	<0.5	1.2 ± 0.8	traces (*R*)
Phenoxyacetone^b^	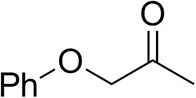	65 ± 1 50 ± 1^d^^,^^e^	81 ± 4 95 ± 1^d^^,^^e^	>99.5 (*R*)
4-Phenylbutanone^b^	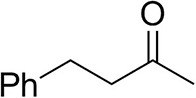	17 ± 1 13 ± 1^d^	51 ± 3 75 ± 1^d^	>99.5 (*R*)

a *Unless otherwise stated, for the carbonyl acceptor scope, 10 mM RMBA was used as the amine donor. Conversions are based on the formation of acetophenone as determined by HPLC and calculated from a calibration curve. All reactions were carried out in triplicate. Standard errors include the SE of the calibration curve (Ellison and Williams, 2012; Theodorou et al., 2012). Any conversion <1% may be due to the exchange of PMP for PLP following the first half reaction with RMBA. Enantiomeric excesses were determined after 24 h by chiral GC-FID or chiral RP-HPLC (butanone, tetrahydrofuran-3-one, β-hydroxy-pyruvate)*.

b *10% (v/v) DMSO*.

c *50 mM isopropylamine*.

d *TsRTA_G205C*.

e *0.1 mg/mL enzyme*.

*“ < 0.5” = calculated conversion smaller than uncertainty from calibration curve. “n.d.” = no product detected*.

*Ts*RTA accepts the commonly used amine donors RMBA, IPA, and d-alanine (d-Ala), with the other enantiomers of MBA and Ala not being accepted. As no pyruvate removal or recycling system was used, the final conversion with d-Ala and benzaldehyde was only 30%, however this was reached within 30 min. Only low conversion was obtained with β-Ala, and only traces with *o*-xylylenediamine (Green et al., 2014). However, *p*-nitrophenethylamine (Baud et al., 2015) was readily accepted, making *Ts*RTA suitable for colourimetric screening during directed evolution (Planchestainer et al., 2019), as was the “smart” amine donor cadaverine (Gomm et al., 2016) (Table 3).

**Table 3:** Amine substrate scope (10 mM scale) of wild-type *Ts*RTA^a^.

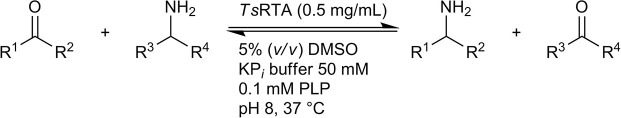			
**Substrate**		**Conversion (%)**	
		**after 30 min**	**after 24 h**
(*R*)-Methylbenzylamine^d^	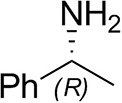	75 ± 1	96 ± 1
(*S*)-Methylbenzylamine^b^	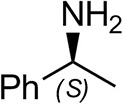	<0.5	<0.5
(±)-Methylbenzylamine^b^^c^	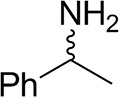	82 ± 3	94 ± 1
L-Alanine	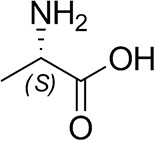	n.d.	n.d.
D-Alanine	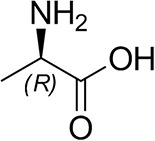	29 ± 4	30 ± 4
(±)-Alanine^c^	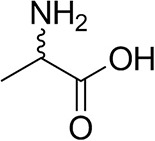	30 ± 4	31 ± 4
β-Alanine	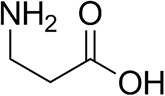	6 ± 5	12 ± 4
Isopropylamine	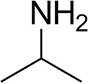	28 ± 4	88 ± 4
Cadaverine	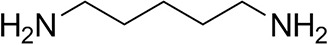	23 ± 4	44 ± 3
*o*-Xylylenediamine	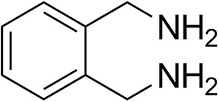	n.d.	7 ± 5
*p*-Nitrophenethylamine	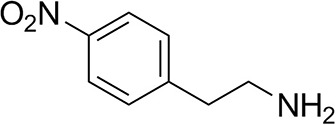	23 ± 4	53 ± 6

a *Unless otherwise stated, 10 mM benzaldehyde was used as the carbonyl acceptor. Conversions are based on the formation of either acetophenone or benzylamine, as determined by HPLC and calculated from a calibration curve. All reactions were carried out in triplicate. Standard errors include the SE of the calibration curve. (Ellison and Williams, 2012; Theodorou et al., 2012)*.

b *10 mM pyruvate*.

c *20 Mm*.

*“ < 0.5” = calculated conversion smaller than uncertainty from calibration curve. “n.d.” = no product detected*.

**Figure 4:**
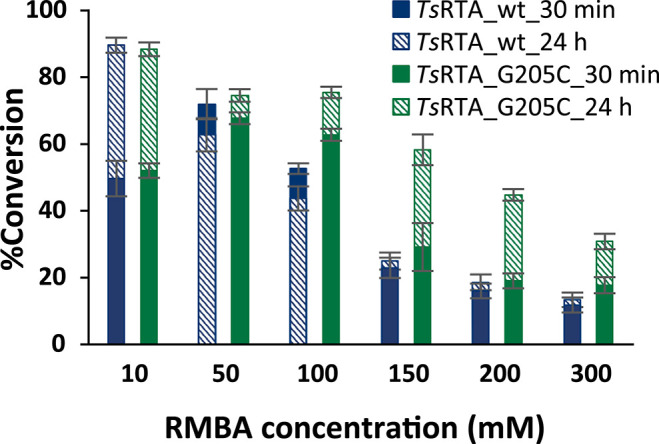
Intensification of biotransformations employing equimolar amounts of RMBA and phenoxyacetone, at 37°C. Reactions contained 0.025 mol% of transaminase, PLP (0.1 mM), DMSO (10% *v/v* for 10–200 mM, 15% for 300 mM), and KP_i_ buffer (50 mM, pH 8). Samples were taken after 30 min and 24 h. Conversions are based on the formation of phenoxypropan-2-amine as determined by HPLC and calculated from a calibration curve. All reactions were carried out in triplicate. Error bars represent standard errors and include the SE of the calibration curve (Ellison and Williams, 2012; Theodorou et al., 2012).

To further investigate the benefit of the increased thermostability of *Ts*RTA_G205C, the intensification of biotransformations with phenoxyacetone with one equivalent of RMBA as the amino donor were investigated for RMBA concentration of 10 to 300 mM (incomplete dissolution of phenoxyacetone for 100 mM and higher), employing a catalyst loading of 0.025 mol%. Both variants showed similar performance at the 10 mM scale, but the wild-type exhibited a faster drop in conversion at increasing scales, reaching 13% conversion at 300 mM vs. 31% for *Ts*RTA_G205C (Figure 4). In addition, the wild-type reached its final conversion within 30 min for concentrations of 50 mM and higher, while *Ts*RTA_G205C showed increased conversions after 30 min even at the 300 mM scale.

## Conclusion

The predominant tetrameric composition in solution of two RTAs, *Ts*RTA and its homolog *At*RTA, has been discovered. Upon comparisons made between the crystal structure of *Ts*RTA and the crystal structures of RTAs deposited in the PDB, a likely interface for the dimer-dimer interaction was also identified. This interface was then probed by introducing a cysteine residue mimicking ATA-117-Rd11, which stabilized both RTAs and thus rationalized the role of this mutation in the directed evolution of ATA-117-Rd11. This dimer-dimer interaction was observed in all but one deposited crystal structure of RTAs, although the position of the equilibrium between the dimeric and tetrameric forms in solution is not known in most cases. SEC studies of the RTA from *Nectria haematococca* have been reported to be consistent with a dimeric form (Sayer et al., 2014). While the wild-type *Ts*RTA was only marginally more stable than *At*RTA, this difference was significantly amplified in the mutant variants. Thus, we propose that two mechanisms, one where tetramer dissociation precedes unfolding and one where unfolding occurs in the tetramer state, might play a role in the inactivation of these RTAs. Clearly, the full inactivation kinetics of both enzymes would need to be studied in detail and in particular the relative kinetic and thermodynamic stability (Bommarius and Paye, 2013) of the tetrameric and dimeric forms for the wild-type enzymes should be elucidated.

The full understanding of the quaternary structure of RTAs is particularly important with regard to rational approaches to enzyme engineering. The stabilization of the tetrameric form of RTAs through the mutation described herein, which could not have been predicted from a dimeric model, appears to be a promising strategy to stabilize RTAs (the residue mutated herein being present in all reported RTA structures). Additional mutations stabilizing this interface may also be possible, creating more stable and therefore more evolvable (Bloom et al., 2006) and industrially useful catalysts.

## Data Availability Statement

The datasets presented in this study can be found in online repositories. The names of the repository/repositories and accession number(s) can be found in the article/Supplementary Material.

## Author Contributions

FP, BD, and CH contributed conception and design of the study. LG carried out protein crystallography, structural analysis, and wrote the relevant sections. CH carried out all other experimental work, data analysis, and wrote the initial manuscript. All authors contributed to manuscript revision, read, and approved the submitted version.

## Conflict of Interest

The authors declare that the research was conducted in the absence of any commercial or financial relationships that could be construed as a potential conflict of interest.

## References

[B1] Baud D. Ladkau N. Moody T. Ward J. M. Hailes H. (2015). A rapid, sensitive colorimetric assay for the high-throughput screening of transaminases in liquid or solid-phase. Chem. Commun. 51, 17225–17228. 10.1039/C5CC06817G 26458082

[B2] Bloom J. D. Labthavikul S. T. Otey C. R. Arnold F. H. (2006). Protein stability promotes evolvability. Proc. Natl. Acad. Sci. U.S.A. 103, 5869–5874. 10.1073/pnas.0510098103 16581913 PMC1458665

[B3] Bommarius A. S. (2015). Biocatalysis: a status report. Annu. Rev. Chem. Biomol. Eng. 6, 319–345. 10.1146/annurev-chembioeng-061114-123415 26247293

[B4] Bommarius A. S. Paye M. F. (2013). Stabilizing biocatalysts. Chem. Soc. Rev. 42, 6534–6565. 10.1039/c3cs60137d 23807146

[B5] Börner T. Rämisch S. Reddem E. R. Bartsch S. Vogel A. Thunnissen A. M. W. H. (2017). Explaining operational instability of amine transaminases: substrate-induced inactivation mechanism and influence of quaternary structure on enzyme-cofactor intermediate stability. ACS Catal. 7, 1259–1269. 10.1021/acscatal.6b02100

[B6] Cerioli L. Planchestainer M. Cassidy J. Tessaro D. Paradisi F. (2015). Characterization of a novel amine transaminase from Halomonas elongata. J. Mol. Catal. B: Enzym. 120, 141–150. 10.1016/j.molcatb.2015.07.009

[B7] Constable D. J. C. Dunn P. J. Hayler J. D. Humphrey G. R. Leazer J. L. Linderman R. J. (2007). Key green chemistry research areas - a perspective from pharmaceutical manufacturers. Green Chem. 9, 411–420. 10.1039/B703488C

[B8] Copeland R. A. (2000). Enzymes. A Practical Introduction to Structure, Mechanism, and Data Analysis. 2nd Edn. New York, NY: Wiley-VCH, Inc.

[B9] Cuetos A. García-Ramos M. Fischereder E. M. Díaz-Rodríguez A. Grogan G. Gotor V.. (2016). Catalytic promiscuity of transaminases: preparation of enantioenriched β-fluoroamines by formal tandem hydrodefluorination/deamination. Angew. Chemie - Int. Ed. 55, 3144–3147. 10.1002/anie.201510554 26836037

[B10] Davies M. T. (1959). A universal buffer solution for use in ultra-violet spectrophotometry. Analyst 84, 248–251. 10.1039/an9598400248

[B11] Davis I. W. Leaver-Fay A. Chen V. B. Block J. N. Kapral G. J. Wang X.. (2007). MolProbity: all-atom contacts and structure validation for proteins and nucleic acids. Nucleic Acids Res. 35, 375–383. 10.1093/nar/gkm216 17452350 PMC1933162

[B12] Dehmlow E. V. Westerheide R. (1992). (S)-3-Aminothiolane: a new chiral building block. Synthesis 10, 947–949. 10.1055/s-1992-26273

[B13] Ellison S. L. R. Williams A. (eds.). (2012). Eurachem/CITAC guide: Quantifying Uncertainty in Analytical Measurement. 3rd Edn. Available online at: https://www.eurachem.org/index.php/publications/guides/quam

[B14] Evans P. R. (2011). An introduction to data reduction: space-group determination, scaling and intensity statistics. Acta Crystallogr. Sect. D. Biol. Crystallogr. 67, 282–292. 10.1107/S090744491003982X 21460446 PMC3069743

[B15] Franchini C. Carocci A. Catalano A. Cavalluzzi M. M. Corbo F. Lentini G.. (2003). Optically active mexiletine analogues as stereoselective blockers of voltage-gated Na^+^ channels. J. Med. Chem. 46, 5238–5248. 10.1021/jm030865y 14613326

[B16] Fuchs M. Farnberger J. E. Kroutil W. (2015). The industrial age of biocatalytic transamination. Eur. J. Org. Chem. 2015, 6965–6982. 10.1002/ejoc.201500852 26726292 PMC4690199

[B17] Gomm A. Lewis W. Green A. P. O'Reilly E. (2016). A new generation of smart amine donors for transaminase-mediated biotransformations. Chem. A Eur. J. 22, 12692–12695. 10.1002/chem.201603188 27411957

[B18] Green A. P. Turner N. J. O'Reilly E. (2014). Chiral amine synthesis using w-transaminases: an amine donor that displaces equilibria and enables high-throughput screening. Angew. Chemie Int. Ed. 53, 10714–10717. 10.1002/anie.201406571 25138082 PMC4497610

[B19] Guo F. Berglund P. (2017). Transaminase biocatalysis: optimization and application. Green Chem. 19, 333–360. 10.1039/C6GC02328B

[B20] Höhne M. Schätzle S. Jochens H. Robins K. Bornscheuer U. T. (2010). Rational assignment of key motifs for function guides *in silico* enzyme identification. Nat. Chem. Biol. 6, 807–813. 10.1038/nchembio.447 20871599

[B21] Isupov M. N. Boyko K. M. Sutter J. M. James P. Sayer C. Schmidt M. (2019). Thermostable branched-chain amino acid transaminases from the archaea geoglobus acetivorans and archaeoglobus fulgidus: biochemical and structural characterization. Front. Bioeng. Biotechnol. 7:79 10.3389/fbioe.2019.00079 30733943 PMC6353796

[B22] Kelly S. A. Pohle S. Wharry S. Mix S. Allen C. C. R. Moody T. S.. (2018). Application of ω-transaminases in the pharmaceutical industry. Chem. Rev. 118, 349–367. 10.1021/acs.chemrev.7b00437 29251912

[B23] Knutsen L. J. S. Lau J. Petersen H. Thomsen C. Weis J. U. Shalmi M.. (1999). N-substituted adenosines as novel neuroprotective A1 agonists with diminished hypotensive effects. J. Med. Chem. 42, 3463–3477. 10.1021/jm960682u 10479279

[B24] Koszelewski D. Lavandera I. Clay D. Rozzell D. Kroutil W. (2008). Asymmetric synthesis of optically pure pharmacologically relevant amines employing ω-transaminases. Adv. Synth. Catal. 350, 2761–2766. 10.1002/adsc.200800496

[B25] Krissinel E. Henrick K. (2004). Secondary-structure matching (SSM), a new tool for fast protein structure alignment in three dimensions. Acta Crystallogr. Sect. D Biol. Crystallogr. 60, 2256–2268. 10.1107/S0907444904026460 15572779

[B26] Laskowski R. A. Watson J. D. Thornton J. M. (2005). ProFunc: a server for predicting protein function from 3D structure. Nucleic Acids Res. 33, W89–W93. 10.1093/nar/gki414 15980588 PMC1160175

[B27] Littlechild J. A. Guy J. Connelly S. Mallett L. Waddell S. Rye C. A.. (2007). Natural methods of protein stabilization: thermostable biocatalysts. Biochem. Soc. Trans. 35, 1558–1563. 10.1042/BST0351558 18031266

[B28] Łyskowski A. Gruber C. Steinkellner G. Schürmann M. Schwab H. Gruber K. (2014). Crystal structure of an (R)-selective ω-transaminase from aspergillus terreus. PloS ONE 9:e87350. 10.1371/journal.pone.0087350 24498081 PMC3907554

[B29] Madeira F. Park Y. Lee J. Buso N. Gur T. Madhusoodanan N.. (2019). The EMBL-EBI search and sequence analysis tools APIs in 2019. Nucleic Acids Res. 47, W636–W641. 10.1093/nar/gkz268 30976793 PMC6602479

[B30] Pan X. Tao X. Ruan L. Li Y. Ou W. Liu F. (2011). An efficient synthesis of (R)-3-aminothiolane. J. Chem. Res. 35, 729–730. 10.3184/174751911X13237056070415

[B31] Planchestainer M. Hegarty E. Heckmann C. M. Gourlay L. J. Paradisi F. (2019). Widely applicable background depletion step enables transaminase evolution through solid-phase screening. Chem. Sci. 10, 5952–5958. 10.1039/C8SC05712E 31360401 PMC6566068

[B32] Powell H. R. Battye T. G. G. Kontogiannis L. Johnson O. Leslie A. G. W. (2017). Integrating macromolecular X-ray diffraction data with the graphical user interface iMosflm. Nat. Protoc. 12, 1310–1325. 10.1038/nprot.2017.037 28569763 PMC5562275

[B33] Savile C. K. Janey J. M. Mundorff E. C. Moore J. C. Tam S. Jarvis W. R.. (2010). Biocatalytic asymmetric synthesis of chiral amines from ketones applied to sitagliptin manufacture. Science 329, 305–310. 10.1126/science.1188934 20558668

[B34] Sayer C. Martinez-Torres R. J. Richter N. Isupov M. N. Hailes H. C. Littlechild J. A.. (2014). The substrate specificity, enantioselectivity and structure of the (R)-selective amine: pyruvate transaminase from Nectria haematococca. FEBS J. 281, 2240–2253. 10.1111/febs.12778 24618038 PMC4255305

[B35] Schätzle S. Höhne M. Redestad E. Robins K. Bornscheuer U. T. (2009). Rapid and sensitive kinetic assay for characterization of ω-transaminases. Anal. Chem. 81, 8244–8248. 10.1021/ac901640q 19739593

[B36] Schätzle S. Steffen-Munsberg F. Thontowi A. Höhne M. Robins K. Bornscheuer U. T. (2011). Enzymatic asymmetric synthesis of enantiomerically pure aliphatic, aromatic and arylaliphatic amines with (R)-selective amine transaminases. Adv. Synth. Catal. 353, 2439–2445. 10.1002/adsc.201100435

[B37] Sheldon R. A. Woodley J. M. (2018). Role of biocatalysis in sustainable chemistry. Chem. Rev. 118, 801–838. 10.1021/acs.chemrev.7b00203 28876904

[B38] Slabu I. Galman J. L. Lloyd R. C. Turner N. J. (2017). Discovery, engineering, and synthetic application of transaminase biocatalysts. ACS Catal. 7, 8263–8284. 10.1021/acscatal.7b02686

[B39] Telzerow A. Paris J. Håkansson M. Gonzalez-Sabin J. Rios-Lombardia N. Schuermann M. (2019). Amine transaminase from exophiala xenobiotica – crystal structure and engineering of a fold IV transaminase that naturally converts biaryl ketones. ACS Catal. 9, 1140–1148. 10.1021/acscatal.8b04524

[B40] Theodorou D. Zannikou Y. Zannikos F. (2012). Estimation of the standard uncertainty of a calibration curve: application to sulfur mass concentration determination in fuels. Accredit. Qual. Assur. 17, 275–281. 10.1007/s00769-011-0852-4

[B41] Thomsen M. Skalden L. Palm G. J. Höhne M. Bornscheuer U. T. Hinrichs W. (2014). Crystallographic characterization of the (R)-selective amine transaminase from *Aspergillus fumigatus*. Acta Crystallogr. Sect. D. Biol. Crystallogr. 70, 1086–1093. 10.1107/S1399004714001084 24699652

[B42] Truppo M. D. (2017). Biocatalysis in the pharmaceutical industry: the need for speed. ACS Med. Chem. Lett. 8, 476–480. 10.1021/acsmedchemlett.7b00114 28523096 PMC5430392

[B43] Vagin A. Teplyakov A. (1997). MOLREP: an automated program for molecular replacement. J. Appl. Crystallogr. 30, 1022–1025. 10.1107/S002188989700676620057045

[B44] Winn M. D. Ballard C. C. Cowtan K. D. Dodson E. J. Emsley P. Evans P. R.. (2011). Overview of the CCP4 suite and current developments. Acta Crystallogr. Sect. D. Biol. Crystallogr. 67, 235–242. 10.1107/S0907444910045749 21460441 PMC3069738

