# Supplementary material for: An (R)-Selective Transaminase From Thermomyces stellatus: Stabilizing the Tetrameric Form
Source: Front Bioeng Biotechnol. 2020 Jul 22;8:707. doi: 10.3389/fbioe.2020.00707 (PMC7387707; doi:10.3389/fbioe.2020.00707)

Supplementary Material

1 Supplementary Figures

```
#
#
#  Percent Identity  Matrix - created by Clustal2.1
#
#
```

1: GEO1900	100.00	78.62	29.31	28.52	30.21	30.93	28.18	28.62	30.34	27.93
2: AF0933	78.62	100.00	32.29	31.49	33.45	31.83	29.76	30.21	30.90	29.86
3: ATA-117	29.31	32.29	100.00	91.82	37.92	40.00	43.12	43.75	42.01	42.99
4: ATA-117-Rd11	28.52	31.49	91.82	100.00	38.13	37.38	39.25	41.25	40.00	40.37
5: HoRTA	30.21	33.45	37.92	38.13	100.00	40.86	43.51	44.97	45.78	42.86
6: Aspergillus-fumigatus	30.93	31.83	40.00	37.38	40.86	100.00	68.42	69.06	72.98	72.36
7: Exophiala-xenobiotica	28.18	29.76	43.12	39.25	43.51	68.42	100.00	73.44	70.06	73.68
8: Nectria-haematococca	28.62	30.21	43.75	41.25	44.97	69.06	73.44	100.00	78.06	77.43
9: TsRTA	30.34	30.90	42.01	40.00	45.78	72.98	70.06	78.06	100.00	81.42
10: AtRTA	27.93	29.86	42.99	40.37	42.86	72.36	73.68	77.43	81.42	100.00

Figure S1: Identity matrix of *Ts*RTA, *Ho*RTA with a variety of previously reported RTAs and two thermostable BCATs, created using MUSCLE (Madeira et al., 2019)**.**

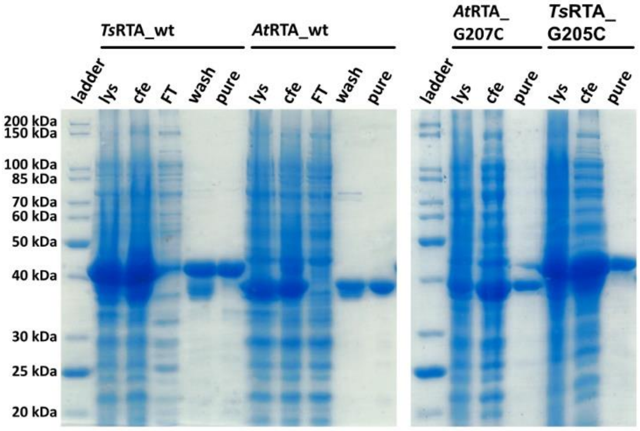

Figure S2: SDS-PAGE gels of *Ts*RTA, *At*RTA, *At*RTA_G207C, *Ts*RTA_G205C. lys: lysate, cfe: cell-free extract, FT: flow-through (IMAC purification), wash: eluted with 10% elution buffer, pure: eluted with 100% elution buffer.

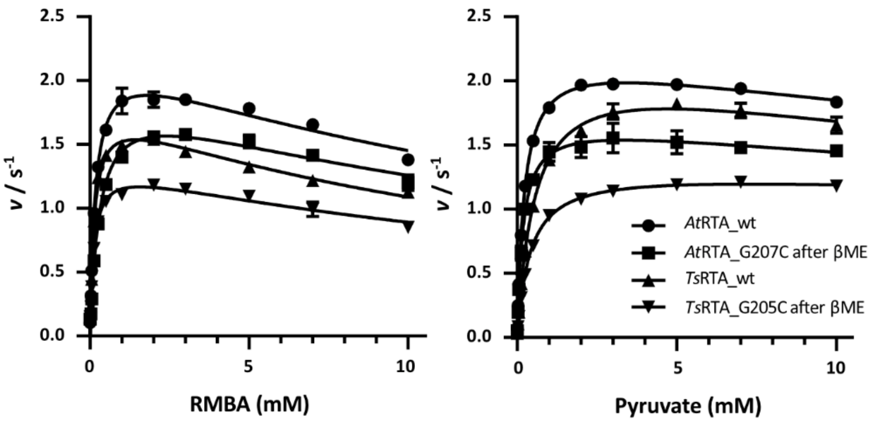

Figure S3: Substrate inhibition curves fitted to the reaction velocities obtained when RMBA and pyruvate were varied, respectively.

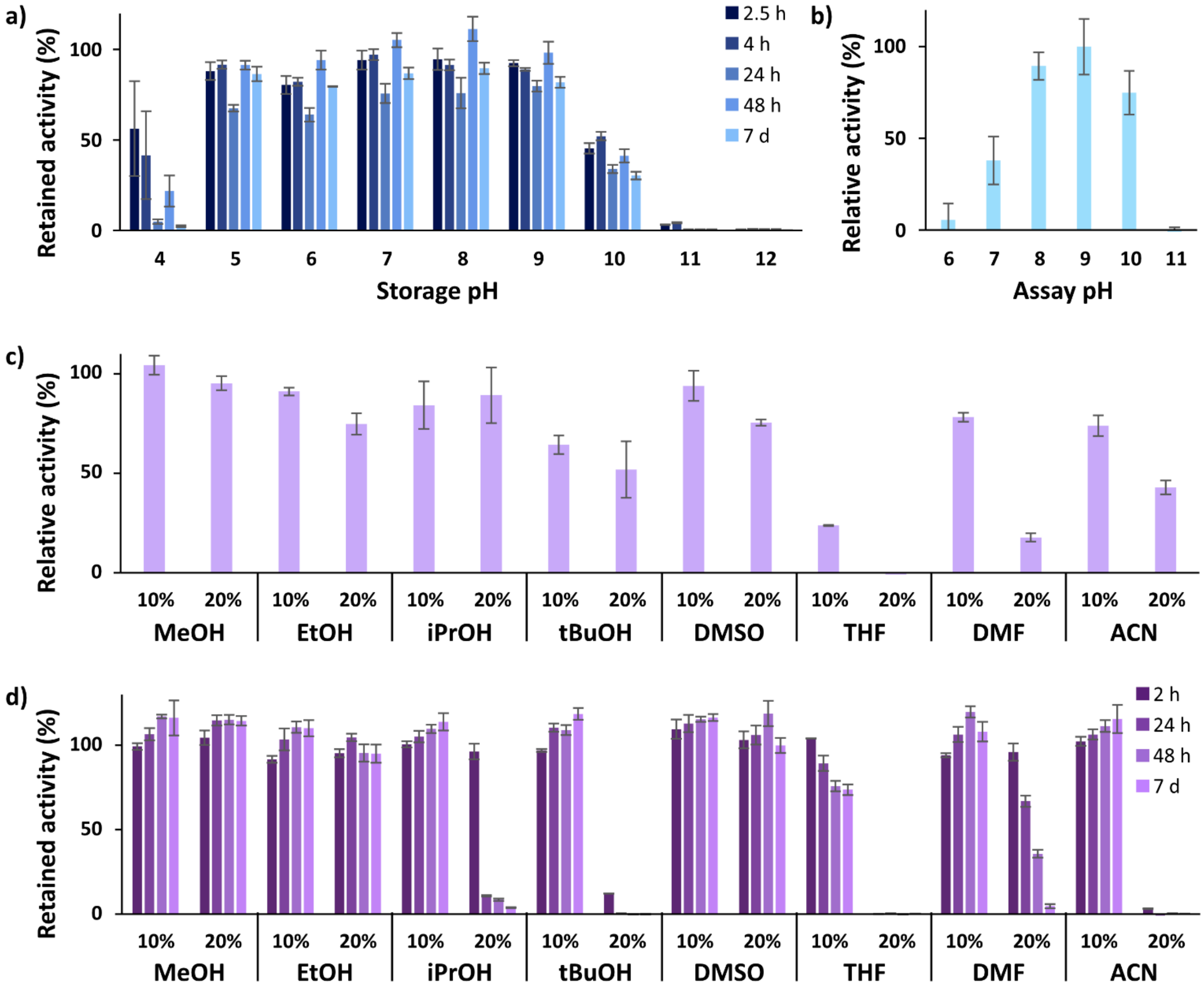

Figure S4: a) pH stability profile of *Ts*RTA: retained activity after incubation at pH 4 – 12 for 2.5 h – 7 d (4 °C), relative to freshly purified enzyme. b) pH–activity relationship of *Ts*RTA: relative activity at pH 6 – 11, relative to maximum activity at pH 9. c) Co-solvent–activity relationship of *Ts*RTA: relative activity in the presence of 10 or 20 % (*v/v*) co-solvents, relative to activity without co-solvent. d) Co-solvent stability profile of *Ts*RTA: retained activity after incubation with 10 or 20 % (*v/v*) co-solvents for 2 h – 7 d (25 °C, pH 8), relative to freshly purified enzyme. Error bars represent standard errors (n=3).

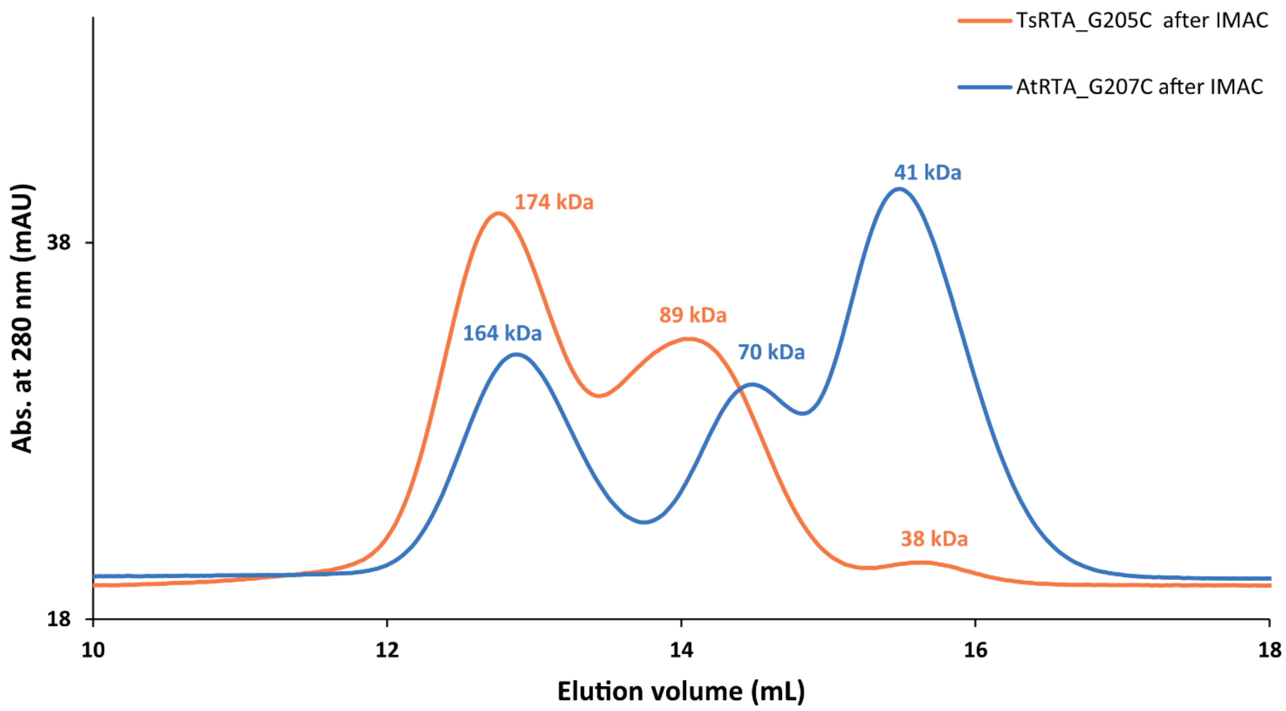

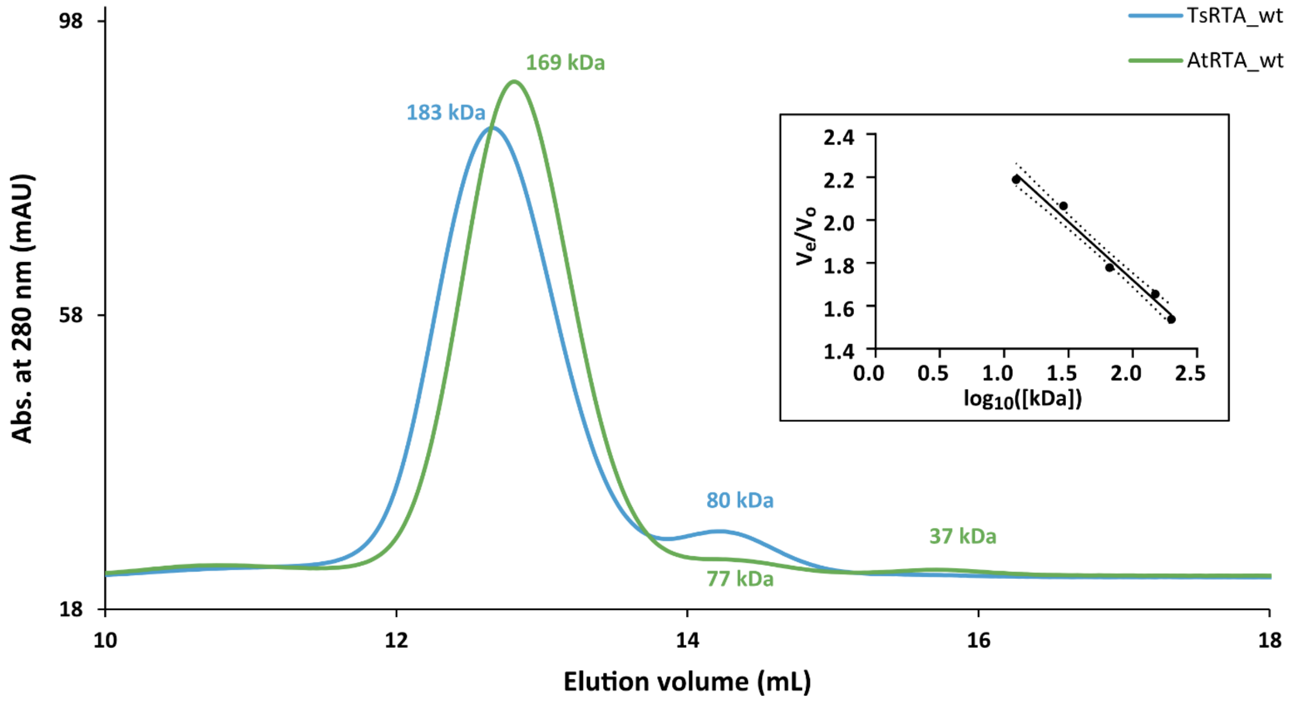

Figure S5: Gel filtration of TsRTA_G205C and AtRTA_G207C immediately after IMAC purification in contrast to the wild-type enzymes, showing the initially disrupted quaternary structure of the mutants. Insert: calibration curve using the Sigma Aldrich Gel Filtration Markers Kit for Protein Molecular Weights 12,000-200,000 Da (MWGF200).

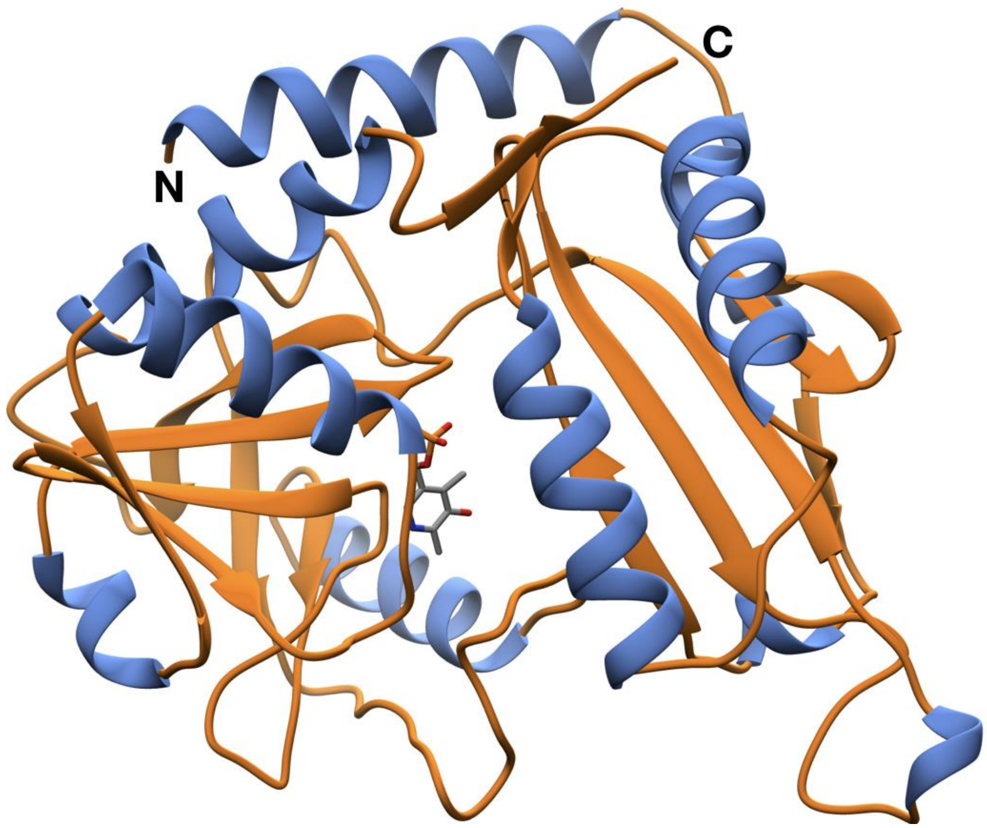

Figure S6: The 3D structure of the *Ts*RTA monomer. Secondary structure ribbon representation of the overall fold of the *Ts*RTA monomer (Chain A) bound to its PLP cofactor (sticks).

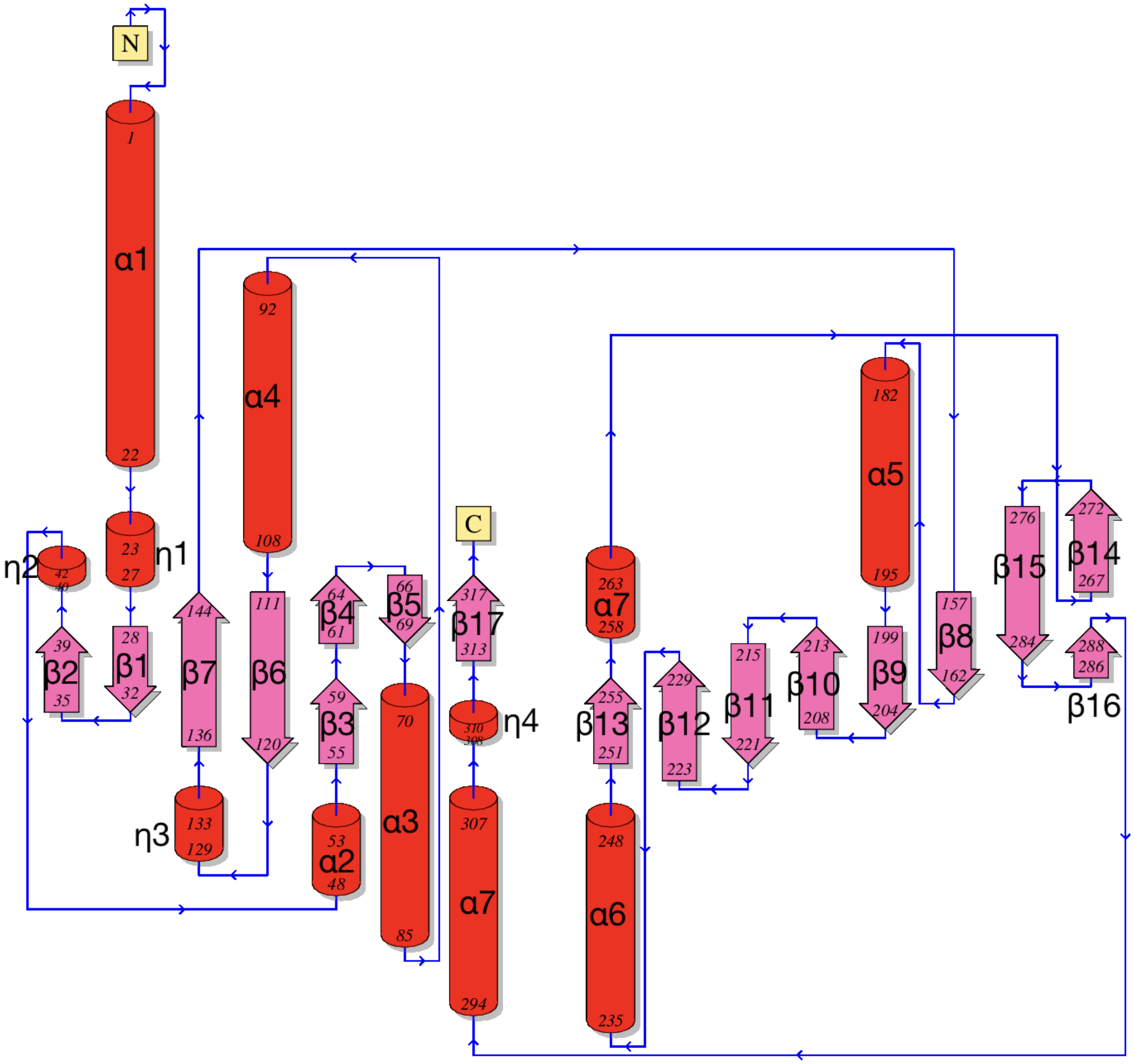

Figure S7: Secondary structure topology diagram of the secondary structure organization of the *Ts*RTA monomer. This diagram generated by the PDBsum server indicates the diverse secondary structure elements (β-strands, α-helices and 3^{10} helices) present in the *Ts*RTA monomer and clearly reveals the presence of two subdomains (Laskowski et al., 2018).

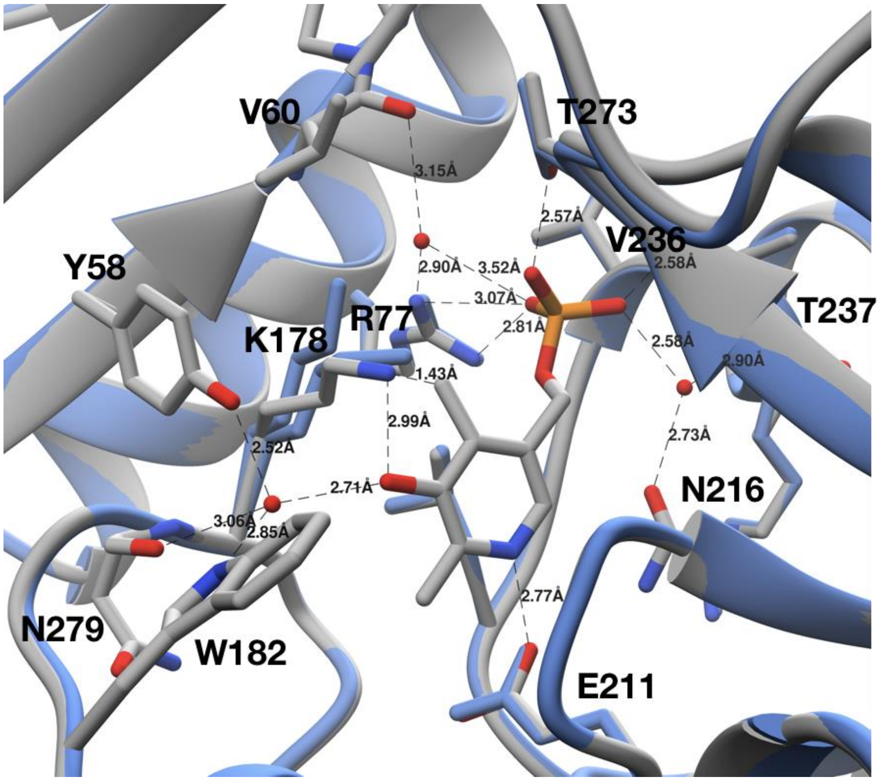

Figure S8: Detailed view of the interactions between PLP and *Ts*RTA. The stabilizing hydrogen bonds formed between active site residues and water molecules and PLP are highlighted. To illustrate the high structural identity between *Ts*RTA and *At*RTA, the structure of the latter (PDB entry 4ce5; blue ribbons and sticks) is superposed with Chain A of *Ts*RTA (grey ribbons and sticks) (Łyskowski et al., 2014). All panels were generated using Chimera (Pettersen et al., 2004).

```
GEO1900                      ---------------------------------MSELLVYMNGEFVPESQAKVSVFDHGF
AF0933                       ------------------------------------MLYVYMDGEFVPENEAKVSIFDHGF
ATA-117                      MAFSADTSEIVYTHDTGLDYITYSDYELDPANPLAGGAAWIEGAFVPPSEARISIFDQGY
ATA-117-Rd11                 MAFSADTPEIVYTHDTGLDYITYSDYELDPANPLAGGAAWIEGAFVPPSEARISIFDQGF
HoRTA                        MNA----------------------------DP-GAGVALIEDEIVPVAEARLPILDWGF
Aspergillus-fumigatus        MASMDKVFSGYYARQKLLERSD---------NPFSKGIAYVEGKLVLPSDARIPLLDEGF
Exophiala-xenobiotica        MATMEKVFAGYEARQKVLEAST---------NPFAKGVAWVEGKLVPVNEARIPLMDQGF
Nectria-haematococca         MATMDKVFAGYAERQAVLEASK---------NPLAKGVAWIQGELVPLHEARIPLLDQGF
TsRTA                        MATMDKVFAGYAARQKAMEAAG---------NPLSEGIAWVEGEMVPLHEARIPMLDEGF
AtRTA                        MASMDKVFAGYAARQAILESTETT-------NPFAKGIAWVEGELVPLAEARIPLLDQGF
                                                                   . ::. :*   :*.:.::* *:

GEO1900                      LYGDGVFEGIRAYNGKVFKLYEHIDRLYDCARVIDLKIPLSKEEFAEAILETLRRNNLRD
AF0933                       LYGDGVFEGIRAYNGRVFRLKEHIDRLYDSAKAIDLEIPITKEEFMEIILETLRKNNLRD
ATA-117                      LHSDVTYTVFHVWNGNAFRLDDHIERLFSNAESMRIIPPLTQDEVKEIALELVAKTELRE
ATA-117-Rd11                 YTSDATYTTFHVWNGNAFRLGDHIERLFSNAESIRLIPPLTQDEVKEIALELVAKTELRE
HoRTA                        LHSDATYDVAHVWQGRFFRLEEHLDRFFAGMDALRMYIPHDREAVSNRLHDLVAASGLRD
Aspergillus-fumigatus        MHSDLTYDVISVWDGRFFRLDDHLQRILESCDKMRLKFPLALSSVKNILAEMVAKSGIRD
Exophiala-xenobiotica        LHSDLTYDVPSVWDGRFFRLDDHLDRFELSCSKMRFKMPLPRQEVKRILVDMVAKSGIKD
Nectria-haematococca         MHSDLTYDVPSVWDGRFFRLEDHLNRLEASCKKMRLRMPLPREEVIKTLVDMVAKSGIRD
TsRTA                        MRSDLTYDVPSVWDGRFFRLDDHLSRLEASCAKLRLKLPLPREEVKKILVEMVAKSGIRD
AtRTA                        MHSDLTYDVPSVWDGRFFRLDDHITRLEASCTKLRLRLPLPRDQVKQILVEMVAKSGIRD
                               .* .:    .::*. *.* :*: *:      : :  *   . .     : :  . :.:

GEO1900                      AYIRPIVTRGA-GDLGLDPRKCPSPNVIIITKP--WGKLYGDLYEKGLKAITVAIRRNAI
AF0933                       AYIRPIVTRGI-GDLGLDPRKCQNPSIIVITKP--WGKLYGDLYEKGLTAITVAVRRNSF
ATA-117                      AFVSVSITRGYSSTPGERDITKHRPQVYMYAVPYQWIVPFDRIRDGVHAMVAQSVRRTPR
ATA-117-Rd11                 AMVTVTITRGYSSTPFERDITKHRPQVYMSACPYQWIVPFDRIRDGVHLMVAQSVRRTPR
HoRTA                        AYVEMICTRGQPRAGSRDPRTCT-NRFLAFAVPFVWIADPAKQETGLHLTISR-MQRIPP
Aspergillus-fumigatus        AFVEVIVTRGLTGVRGSKPEDLYNNNIYLLVLPYIWVMAPENQLHGGEAIITRTVRRTPP
Exophiala-xenobiotica        AFVEIIVTRGLKGVRGLKAGESLTNNLYMWIQPYIWVMEPEMQRTGGSAIIARTVKRTSP
Nectria-haematococca         AFVELIVTRGLTGVRGAKPEELLNNNLYMFIQPYVWVMDPDVQYTGGRAIVARTVRRVPP
TsRTA                        AFVEIIVTRGLKGVRGSRPEEIV-NRLYMLVQPYVWVMEPEVQPVGGDAVIARTVRRVPP
AtRTA                        AFVELIVTRGLKGVRGTRPEDIV-NNLYMFVQPYVWVMEPDMQRVGGSAVVARTVRRVPP
                             * :    ***                .     *  *              ::  :.* .

GEO1900                      DSLPPNIKSLNYLNNILAKIEANAKGGDEAIFLDHNGYISEGSGDNIFIVKNGTITTPPT
AF0933                       DALPPNIKSLNYLNNILAKIEANAKGGDEAIFLDRNGYVSEGSGDNIFVVKNGAITTPPT
ATA-117                      SSIDPQVKNFQWGDLIRAVQETHDRGFEAPLLLDGDGLLAEGSGFNVVVIKDGVVRSPGR
ATA-117-Rd11                 SSIDPQVKNFQWGDLIRAIQETHDRGFELPLLLDCDNLLAEGPGFNVVVIKDGVVRSPGR
HoRTA                        ASVDPTVKNYHWLDMVQALFEAYDRGAETAITVDAEDNVVEGPGFNLFVVQGGDLATPAT
Aspergillus-fumigatus        GAFDPTIKNLQWGDLTKGLFEAMDRGATYPFLTDGDTNLTEGSGFNIVLVKNGIIYTPDR
Exophiala-xenobiotica        GSMDPTVKNLQWGDLTRGMLEAQDRGADYPFLTDGDGNITEGSGFNIVFIKDGVLYTPDR
Nectria-haematococca         GSIDPTIKNLQWGDLVRGLFEANDRGATYPFLTDGDANLTEGSGFNVVLIKDGVLYTPDR
TsRTA                        GSIDPTVKNLQWGDFVRGLFEASDRGATYPFLTDGDANLTEGSGFNIVLVKDGVLYTPDR
AtRTA                        GAIDPTVKNLQWGDLVRGMFEAADRGATYPFLTDGDAHLTEGSGFNIVLVKDGVLYTPDR
                              :. * :*. :: :   .  *:  .*   .:  * :  : **.* *:..::.* : :*

GEO1900                      LNNLKGITRQVVIELINELEIPFREANIGLFDLYSADEIFVTGTAAEIAPVTYIDGRTVG
AF0933                       INNLRGITREAVIEIINRLGIPFKETNIGLYDLYTADEVFVTGTAAEIAPIVVIDGRKIG
ATA-117                      -AALPGITRKTVLEIAESLGHEAILADITLAELLDADEVLGCTTAGGVWPFVSVDGNPIS
ATA-117-Rd11                 -AALPGITRKTVLEIAESLGHEAILADITPAELYDADEVLGCSTGGGVWPFVSVDGNSIS
HoRTA                        -GVLPGVTRRTVIELGDAHGRGVQAQSVRADTVRGADEVFITSTAGGVMPVTRVDGALIG
Aspergillus-fumigatus        -GVLRGITRKSVIDVARANSIDIRLEVVPVEQAYHSDEIFMCTTAGGIMPITLLDGQPVN
Exophiala-xenobiotica        -GVLKGVTRKSVADAAKANGIEMRIEFVPTEMAYQCDECFMCTTAGGVMPITSMDGQPIG
Nectria-haematococca         -GVLQGITRKSVIDAARSCGYEIRVEHVPIEATYQADEILMCTTAGGIMPITTLDDKPVK
TsRTA                        -GVLQGVTRKSVIDVANAKGFEVRVEYVPVEAAYHADEIFMCTTAGGIMPIRSLDGKPVN
AtRTA                        -GVLQGVTRKSVINAAEAFGIEVRVEFVPVELAYRCDEIFMCTTAGGIMPITTLDGMPVN
                                * *:**  * :             :       .** :   *.. : *.   :*.  :

GEO1900                      NGKPGKVTKMLMEKFRERTENEGVEI----YR--------------
AF0933                       DGKPGEITRKLMEEFSKLTESEGVPI----YE--------------
ATA-117                      DGVPGPVTQSIIRRYWELNVESSSLLTPVQY---------------
ATA-117-Rd11                 DGVPGPVTQSIIRRYWELNVEPSSLLTPVQYA--------------
HoRTA                        DGKPGPVTRALRDAYWRLHEEPWYTQ-AVSYERA-----AGVRHGD
Aspergillus-fumigatus        DGQVGPITKKIWDGYWEMHYNPAYSF-PVDYGSG------------
Exophiala-xenobiotica        DGKVGPVTKEIWDGYWAMHYDDKYSF-KIDYEEKSANGTNGVNGVH
Nectria-haematococca         DGKVGPITKAIWDRYWAMHWEDEFSF-KINY---------------
TsRTA                        DGKVGPITKAIWDGYWEMHYDPAYSF-EIKYQVAEGKPLAGYRFQE
AtRTA                        GGQIGPITKKIWDGYWAMHYDAAYSF-EIDYNERN-----------
                             .*  * :*. :   :     .         *
```

Figure S9: CLUSTAL multiple sequence alignment by MUSCLE (3.8) (Madeira et al., 2019): Bold red: residues differing from *At*RTA. Highlighted in blue: Cysteine forming a disulfide bridge in the tetrameric structure of ATA-117-RD11, and the corresponding glycine in ATA-117.

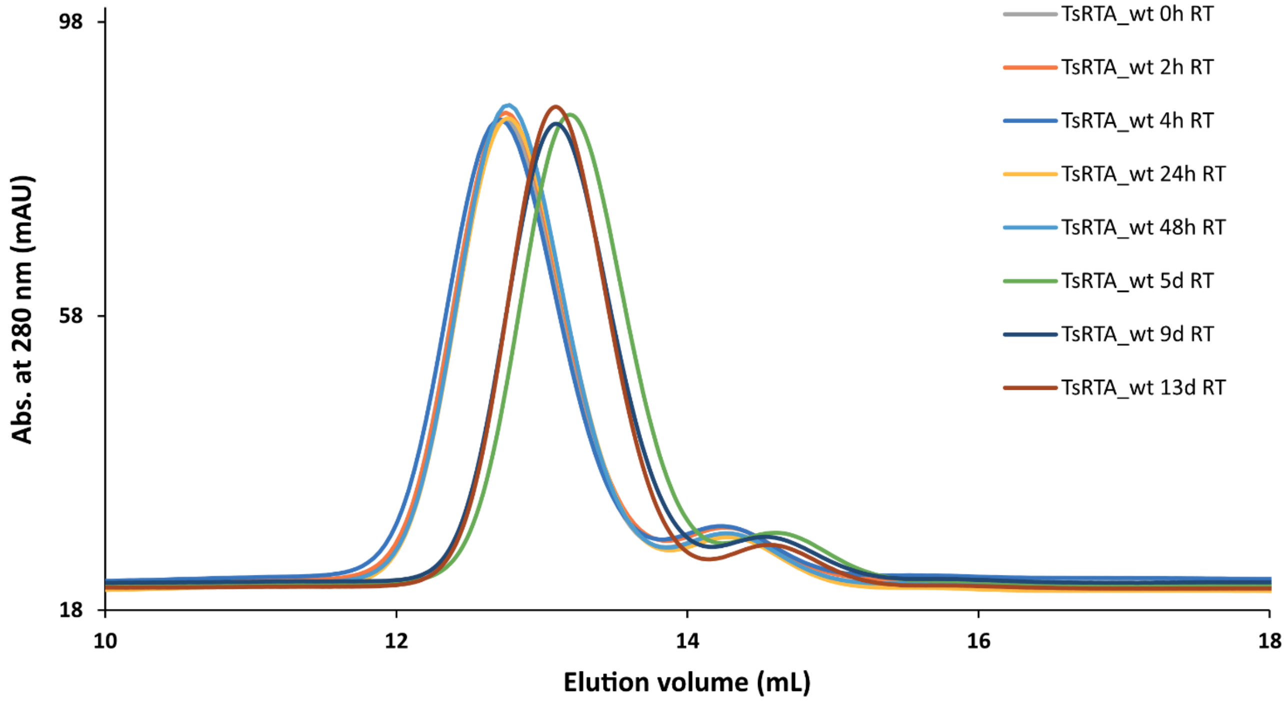

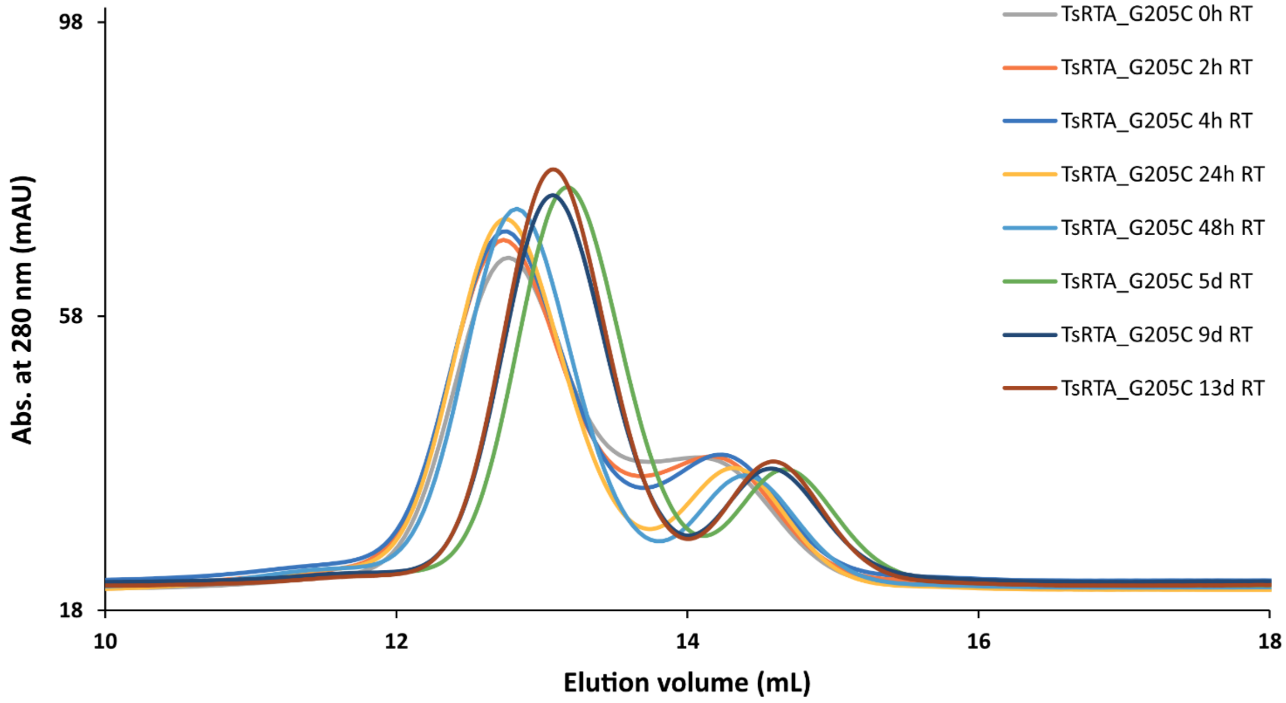

Figure S10: Gel-filtration chromatograms following the incubation of wild-type and mutant TsRTA at ambient temperature with gentle agitation (aeration).

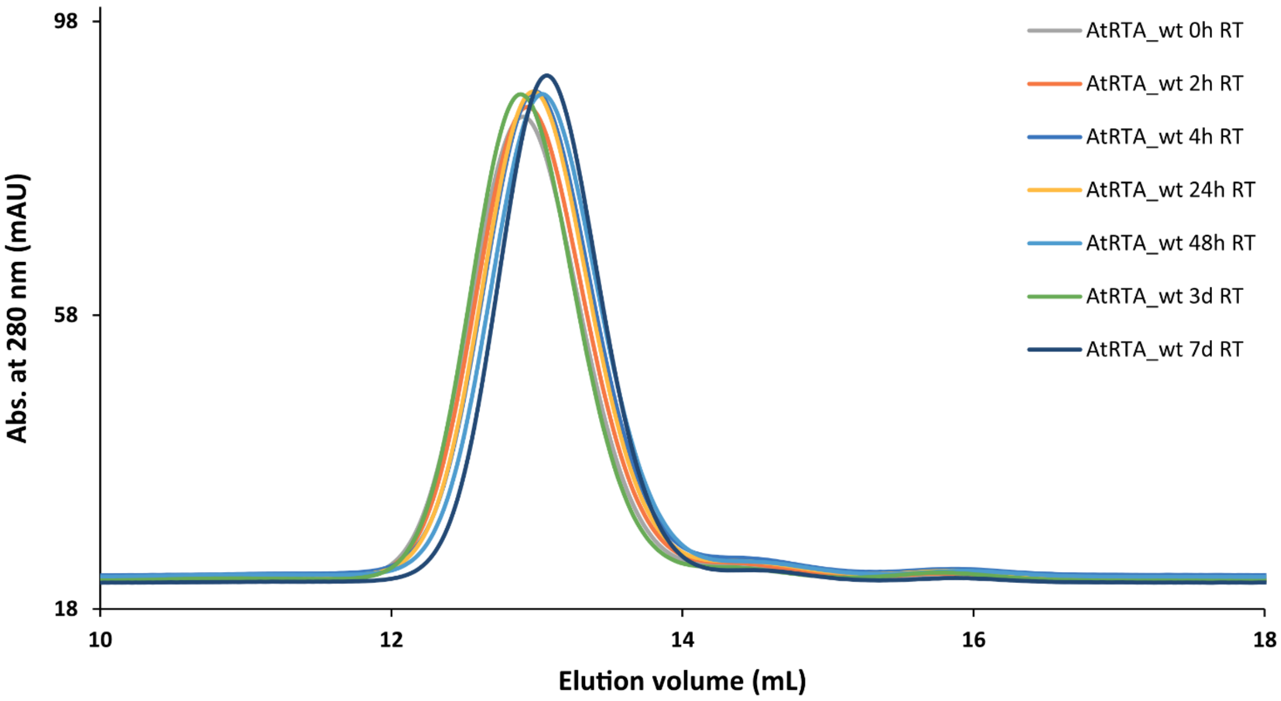

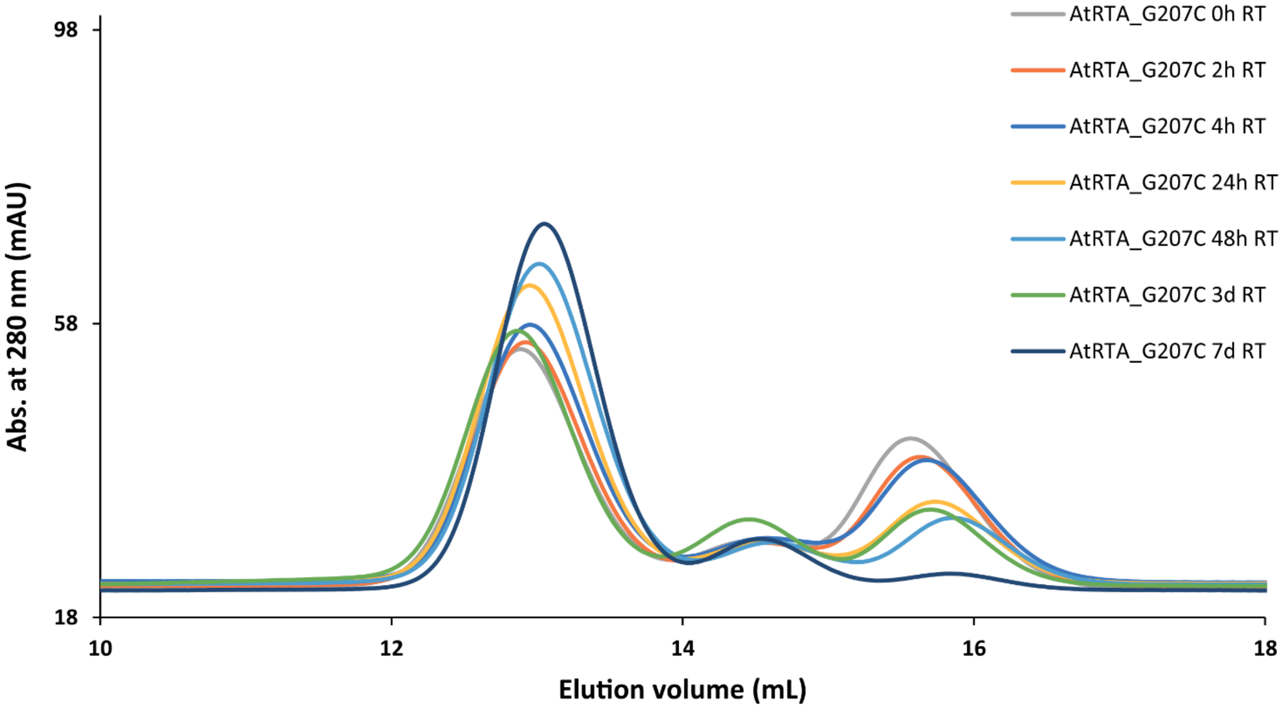

Figure S11: Gel-filtration chromatograms following the incubation of wild-type and mutant AtRTA at ambient temperature with gentle agitation (aeration).

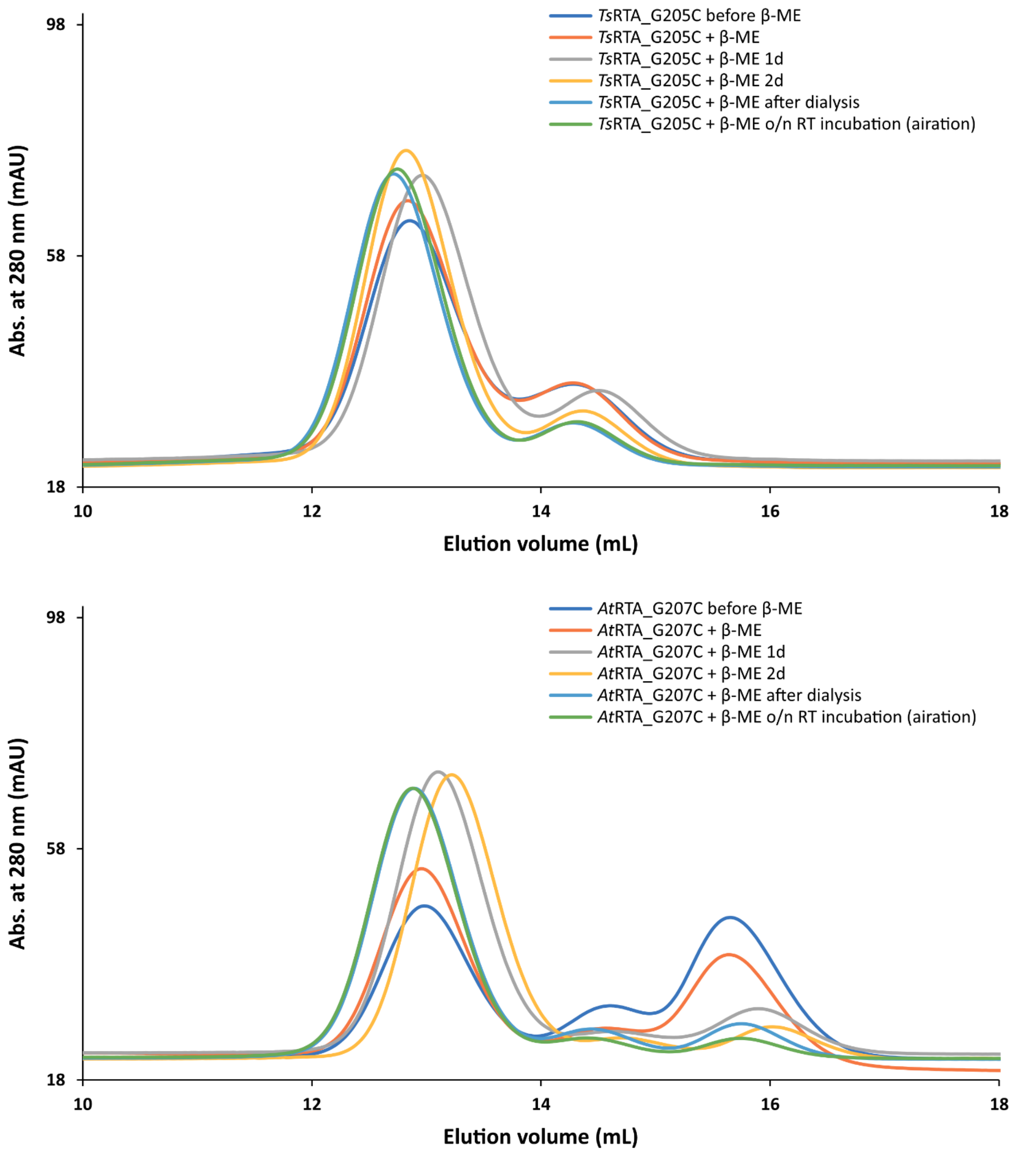

Figure S12: Gel-filtration chromatograms following the β-mercaptoethanol treatment of *Ts*RTA_G205C and *At*RTA_G207C.

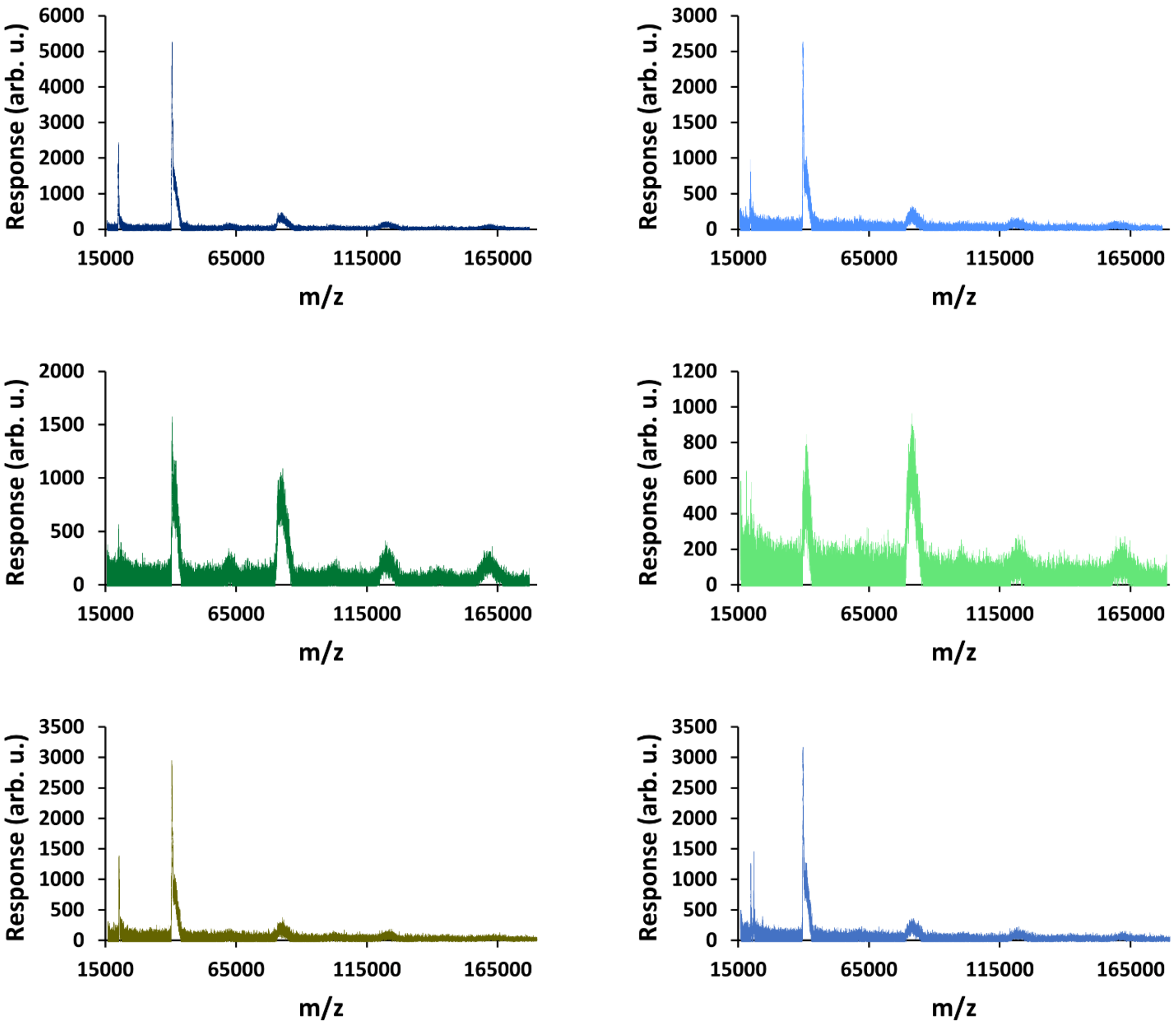

Figure S13: MALDI-TOF MS traces. Left: TsRTA, right: AtRTA. Top: wt. Middle: mutant. Bottom: mutant after incubation with TCEP.

2 Supplementary Tables

Table S1: Data collection statistics and refinement parameters for TsRTA.[a]

	***Ts*RTA**
**Data collection**	
Space group	I_12_1
Cell dimensions	
a, *b*, *c* (Å)	67.8, 98.0, 117.3
α, β, γ (°)	90.0, 91.2, 90.0
Resolution (Å)	59-2.2 (2.2-2.27)
$^aR_{merge}$	0.112 (0.526)
$I / \sigma I$	10.2 (2.7)
Completeness (%)	100 (100)
Redundancy	6.1(6.2)
$^bCC^{1/2}$	100 (91.2)
**Refinement**	
Resolution (Å)	2.2-59.0
No. unique reflections	38887
$^cR_{work}$ / $^dR_{free}$	20.7/23.4
No. atoms	
Protein	2482(A)2496(B)
Ethylene glycol	114
PLP	30
Sodium ion	2
Water	165
B-factors (Å^2)	
Protein	29 (A) 31 (B)
Water	27 (A) 26 (B)
Ethylene glycol	32
PLP	23
Sodium ion	26
Water	25
RMSD:	
Bond lengths (Å)	0.003
Bond angles (°)	0.598
Ramachandran Plot (%)	
Allowed Regions	100

[a]Data were collected AT 2.2Å on a single TsRTA crystal. Parentheses indicate parameters related to the high-resolution cell (2.2 – 2.27 Å). $^aR_{merge} = \sum | I - (I) | / \sum I \times 100$, where I is the intensity of a reflection and *(I)* is the average intensity. $^bCC^{1/2}$ is the correlation between random half-sets of data. $^cR_{work} = \Sigma|F_o - F_c| / \Sigma F_o \times 100$; [d]For cross-validation, 10 % experimental reflections were randomly selected to calculate the R_{free}.

3 Supplementary References

Laskowski, R. A., Jabłońska, J., Pravda, L., Vařeková, R. S., and Thornton, J. M. (2018). PDBsum: Structural summaries of PDB entries. *Protein Sci.* 27, 129–134. doi:10.1002/pro.3289.

Łyskowski, A., Gruber, C., Steinkellner, G., Schürmann, M., Schwab, H., Gruber, K., et al. (2014). Crystal structure of an (R)-selective ω-transaminase from Aspergillus terreus. *PloS one* 9, e87350. doi:10.1371/journal.pone.0087350.

Madeira, F., Park, Y. mi, Lee, J., Buso, N., Gur, T., Madhusoodanan, N., et al. (2019). The EMBL-EBI search and sequence analysis tools APIs in 2019. *Nucleic Acids Res.* 47, W636–W641. doi:10.1093/nar/gkz268.

Pettersen, E. F., Goddard, T. D., Huang, C. C., Couch, G. S., Greenblatt, D. M., Meng, E. C., et al. (2004). UCSF Chimera?A visualization system for exploratory research and analysis. *J. Comput. Chem.* 25, 1605–1612. doi:10.1002/jcc.20084.

4 Sequences

4.1 pCH93b

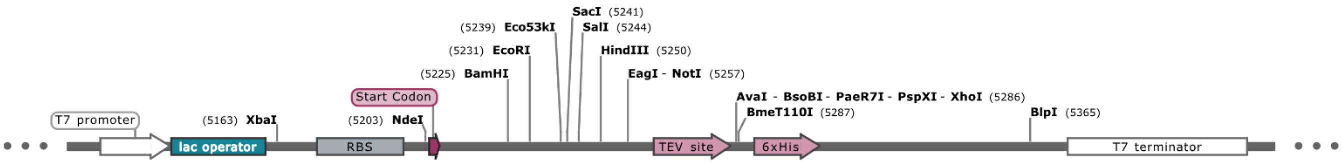

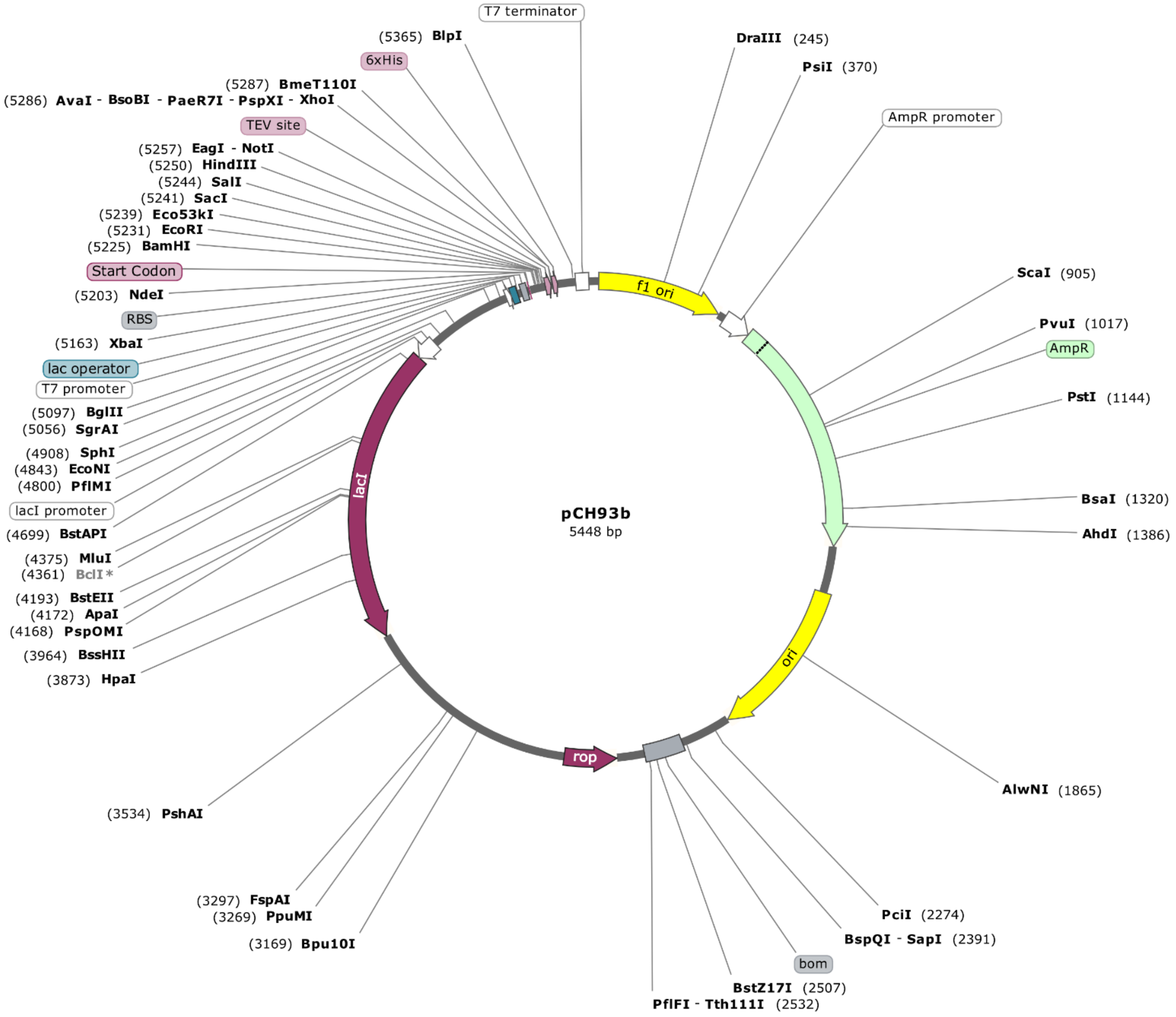

TGGCGAATGGGACGCGCCCTGTAGCGGCGCATTAAGCGCGGCGGGTGTGGTGGTTACGCGCAGCGTGACCGCTACACTTGCCAGCGCCCT
AGCGCCCGCTCCTTTCGCTTTCTTCCCTTCCTTTCTCGCCACGTTCGCCGGCTTTCCCCGTCAAGCTCTAAATCGGGGGCTCCCTTTAGG
GTTCCGATTTAGTGCTTTACGGCACCTCGACCCCAAAAAACTTGATTAGGGTGATGGTTCACGTAGTGGGCCATCGCCCTGATAGACGGT

TTTTCGCCCTTTGACGTTGGAGTCCACGTTCTTTAATAGTGGACTCTTGTTCCAAACTGGAACAACACTCAACCCTATCTCGGTCTATTC
TTTTGATTTATAAGGGATTTTGCCGATTTCGGCCTATTGGTTAAAAAATGAGCTGATTTAACAAAAATTTAACGCGAATTTTAACAAAAT
ATTAACGTTTACAATTTCAGGTGGCACTTTTCGGGGAAATGTGCGCGGAACCCCTATTTGTTTATTTTTCTAAATACATTCAAATATGTA
TCCGCTCATGAGACAATAACCCTGATAAATGCTTCAATAATATTGAAAAAGGAAGAGTATGAGTATTCAACATTTCCGTGTCGCCCTTAT
TCCCTTTTTTGCGGCATTTTGCCTTCCTGTTTTTGCTCACCCAGAAACGCTGGTGAAAGTAAAAGATGCTGAAGATCAGTTGGGTGCACG
AGTGGGTTACATCGAACTGGATCTCAACAGCGGTAAGATCCTTGAGAGTTTTCGCCCCGAAGAACGTTTTCCAATGATGAGCACTTTTAA
AGTTCTGCTATGTGGCGCGGTATTATCCCGTATTGACGCCGGGCAAGAGCAACTCGGTCGCCGCATACACTATTCTCAGAATGACTTGGT
TGAGTACTCACCAGTCACAGAAAAGCATCTTACGGATGGCATGACAGTAAGAGAATTATGCAGTGCTGCCATAACCATGAGTGATAACAC
TGCGGCCAACTTACTTCTGACAACGATCGGAGGACCGAAGGAGCTAACCGCTTTTTTGCACAACATGGGGGATCATGTAACTCGCCTTGA
TCGTTGGGAACCGGAGCTGAATGAAGCCATACCAAACGACGAGCGTGACACCACGATGCCTGCAGCAATGGCAACAACGTTGCGCAAACT
ATTAACTGGCGAACTACTTACTCTAGCTTCCCGGCAACAATTAATAGACTGGATGGAGGCGGATAAAGTTGCAGGACCACTTCTGCGCTC
GGCCCTTCCGGCTGGCTGGTTTATTGCTGATAAATCTGGAGCCGGTGAGCGTGGGTCTCGCGGTATCATTGCAGCACTGGGGCCAGATGG
TAAGCCCTCCCGTATCGTAGTTATCTACACGACGGGGAGTCAGGCAACTATGGATGAACGAAATAGACAGATCGCTGAGATAGGTGCCTC
ACTGATTAAGCATTGGTAACTGTCAGACCAAGTTTACTCATATATACTTTAGATTGATTTAAAACTTCATTTTTAATTTAAAAGGATCTA
GGTGAAGATCCTTTTTGATAATCTCATGACCAAAATCCCTTAACGTGAGTTTTCGTTCCACTGAGCGTCAGACCCCGTAGAAAAGATCAA
AGGATCTTCTTGAGATCCTTTTTTTCTGCGCGTAATCTGCTGCTTGCAAACAAAAAAACCACCGCTACCAGCGGTGGTTTGTTTGCCGGA
TCAAGAGCTACCAACTCTTTTTCCGAAGGTAACTGGCTTCAGCAGAGCGCAGATACCAAATACTGTCCTTCTAGTGTAGCCGTAGTTAGG
CCACCACTTCAAGAACTCTGTAGCACCGCCTACATACCTCGCTCTGCTAATCCTGTTACCAGTGGCTGCTGCCAGTGGCGATAAGTCGTG
TCTTACCGGGTTGGACTCAAGACGATAGTTACCGGATAAGGCGCAGCGGTCGGGCTGAACGGGGGGTTCGTGCACACAGCCCAGCTTGGA
GCGAACGACCTACACCGAACTGAGATACCTACAGCGTGAGCTATGAGAAAGCGCCACGCTTCCCGAAGGGAGAAAGGCGGACAGGTATCC
GGTAAGCGGCAGGGTCGGAACAGGAGAGCGCACGAGGGAGCTTCCAGGGGGAAACGCCTGGTATCTTTATAGTCCTGTCGGGTTTCGCCA
CCTCTGACTTGAGCGTCGATTTTTGTGATGCTCGTCAGGGGGGCGGAGCCTATGGAAAAACGCCAGCAACGCGGCCTTTTTACGGTTCCT
GGCCTTTTGCTGGCCTTTTGCTCACATGTTCTTTCCTGCGTTATCCCCTGATTCTGTGGATAACCGTATTACCGCCTTTGAGTGAGCTGA
TACCGCTCGCCGCAGCCGAACGACCGAGCGCAGCGAGTCAGTGAGCGAGGAAGCGGAAGAGCGCCTGATGCGGTATTTTCTCCTTACGCA
TCTGTGCGGTATTTCACACCGCATATATGGTGCACTCTCAGTACAATCTGCTCTGATGCCGCATAGTTAAGCCAGTATACACTCCGCTAT
CGCTACGTGACTGGGTCATGGCTGCGCCCCGACACCCGCCAACACCCGCTGACGCGCCCTGACGGGCTTGTCTGCTCCCGGCATCCGCTT
ACAGACAAGCTGTGACCGTCTCCGGGAGCTGCATGTGTCAGAGGTTTTCACCGTCATCACCGAAACGCGCGAGGCAGCTGCGGTAAAGCT
CATCAGCGTGGTCGTGAAGCGATTCACAGATGTCTGCCTGTTCATCCGCGTCCAGCTCGTTGAGTTTCTCCAGAAGCGTTAATGTCTGGC
TTCTGATAAAGCGGGCCATGTTAAGGGCGGTTTTTTCCTGTTTGGTCACTGATGCCTCCGTGTAAGGGGGATTTCTGTTCATGGGGGTAA
TGATACCGATGAAACGAGAGAGGATGCTCACGATACGGGTTACTGATGATGAACATGCCCGGTTACTGGAACGTTGTGAGGGTAAACAAC
TGGCGGTATGGATGCGGCGGGACCAGAGAAAAATCACTCAGGGTCAATGCCAGCGCTTCGTTAATACAGATGTAGGTGTTCCACAGGGTA
GCCAGCAGCATCCTGCGATGCAGATCCGGAACATAATGGTGCAGGGCGCTGACTTCCGCGTTTCCAGACTTTACGAAACACGGAAACCGA
AGACCATTCATGTTGTTGCTCAGGTCGCAGACGTTTTGCAGCAGCAGTCGCTTCACGTTCGCTCGCGTATCGGTGATTCATTCTGCTAAC
CAGTAAGGCAACCCCGCCAGCCTAGCCGGGTCCTCAACGACAGGAGCACGATCATGCGCACCCGTGGGGCCGCCATGCCGGCGATAATGG
CCTGCTTCTCGCCGAAACGTTTGGTGGCGGGACCAGTGACGAAGGCTTGAGCGAGGGCGTGCAAGATTCCGAATACCGCAAGCGACAGGC
CGATCATCGTCGCGCTCCAGCGAAAGCGGTCCTCGCCGAAAATGACCCAGAGCGCTGCCGGCACCTGTCCTACGAGTTGCATGATAAAGA
AGACAGTCATAAGTGCGGCGACGATAGTCATGCCCCGCGCCCACCGGAAGGAGCTGACTGGGTTGAAGGCTCTCAAGGGCATCGGTCGAG
ATCCCGGTGCCTAATGAGTGAGCTAACTTACATTAATTGCGTTGCGCTCACTGCCCGCTTTCCAGTCGGGAAACCTGTCGTGCCAGCTGC
ATTAATGAATCGGCCAACGCGCGGGGAGAGGCGGTTTGCGTATTGGGCGCCAGGGTGGTTTTTCTTTTCACCAGTGAGACGGGCAACAGC
TGATTGCCCTTCACCGCCTGGCCCTGAGAGAGTTGCAGCAAGCGGTCCACGCTGGTTTGCCCCAGCAGGCGAAAATCCTGTTTGATGGTG
GTTAACGGCGGGATATAACATGAGCTGTCTTCGGTATCGTCGTATCCCACTACCGAGATATCCGCACCAACGCGCAGCCCGGACTCGGTA
ATGGCGCGCATTGCGCCCAGCGCCATCTGATCGTTGGCAACCAGCATCGCAGTGGGAACGATGCCCTCATTCAGCATTTGCATGGTTTGT
TGAAAACCGGACATGGCACTCCAGTCGCCTTCCCGTTCCGCTATCGGCTGAATTTGATTGCGAGTGAGATATTTATGCCAGCCAGCCAGA
CGCAGACGCGCCGAGACAGAACTTAATGGGCCCGCTAACAGCGCGATTTGCTGGTGACCCAATGCGACCAGATGCTCCACGCCCAGTCGC
GTACCGTCTTCATGGGAGAAAATAATACTGTTGATGGGTGTCTGGTCAGAGACATCAAGAAATAACGCCGGAACATTAGTGCAGGCAGCT
TCCACAGCAATGGCATCCTGGTCATCCAGCGGATAGTTAATGATCAGCCCACTGACGCGTTGCGCGAGAAGATTGTGCACCGCCGCTTTA
CAGGCTTCGACGCCGCTTCGTTCTACCATCGACACCACCACGCTGGCACCCAGTTGATCGGCGCGAGATTTAATCGCCGCGACAATTTGC
GACGGCGCGTGCAGGGCCAGACTGGAGGTGGCAACGCCAATCAGCAACGACTGTTTGCCCGCCAGTTGTTGTGCCACGCGGTTGGGAATG
TAATTCAGCTCCGCCATCGCCGCTTCCACTTTTTCCCGCGTTTTCGCAGAAACGTGGCTGGCCTGGTTCACCACGCGGGAAACGGTCTGA
TAAGAGACACCGGCATACTCTGCGACATCGTATAACGTTACTGGTTTCACATTCACCACCCTGAATTGACTCTCTTCCGGGCGCTATCAT
GCCATACCGCGAAAGGTTTTGCGCCATTCGATGGTGTCCGGGATCTCGACGCTCTCCCTTATGCGACTCCTGCATTAGGAAGCAGCCCAG
TAGTAGGTTGAGGCCGTTGAGCACCGCCGCCGCAAGGAATGGTGCATGCAAGGAGATGGCGCCCAACAGTCCCCCGGCCACGGGGCCTGC
CACCATACCCACGCCGAAACAAGCGCTCATGAGCCCGAAGTGGCGAGCCCGATCTTCCCCATCGGTGATGTCGGCGATATAGGCGCCAGC
AACCGCACCTGTGGCGCCGGTGATGCCGGCCACGATGCGTCCGGCGTAGAGGATCGAGATCTCGATCCCGCGAAATTAATACGACTCACT
ATAGGGGAATTGTGAGCGGATAACAATTCCCCTCTAGAAATAATTTTGTTTAACTTTAAGAAGGAGATATACATATGGATATCGGAATTA
ATTCGGATCCGAATTCGAGCTCCGTCGACAAGCTTGCGGCCGCA**GAGAACCTCTATTTCCAAGGG**CTCGAGCACCACCACCACCACCACT
GAGATCCGGCTGCTAACAAAGCCCGAAAGGAAGCTGAGTTGGCTGCTGCCACCGCTGAGCAATAACTAGCATAACCCCTTGGGGCCTCTA
AACGGGTCTTGAGGGGTTTTTTGCTGAAAGGAGGAACTATATCCGGAT

Legend: ACGCGC…CGCTTACAATTT f1 ori

TTCAAATATGT…AGACAAT	AmpR promoter
ATGAGTATTCAAC…GGTAA	AmpR
TTGAGATCCTTT…TGGAAA	pBR322/pUC ori
TCAGAGGTTTTC…GGTCAC	rop (anti-sense)
CACTG…TAACGGGTTTCAC	LacI (anti-sense)
ATTCACCA…CGATGGTGTC	LacI promoter ("antisense")
TAATACGACTCACTATAGG	T7 promoter
GGAATTGT…AACAATTCC	lac operator
AAGGAG	RBS
ATG	Start
CACCACCACCACCACCAC	His-tag
TGA	Stop
CTAGCAT…GGGGTTTTTTG	T7 terminator
**GAGAACCTCTATTTCCAAGGG**	TEV recognition sequence

4.2 TsRTA

DNA sequence:

…GGATCCG**ATGGCAACAATGGATAAAGTTTTTGCAGGTTATGCAGCACGTCAGAAAGCAATGGAAGCAGCAGGTAATCCGCTGAGCGAAG**
GTATTGCATGGGTTGAAGGTGAAATGGTTCCGCTGCATGAAGCACGTATTCCGATGCTGGATGAAGGTTTTATGCGTAGCGATCTGACCT
ATGATGTTCCGAGCGTTTGGGATGGTCGTTTTTTTCGTCTGGATGATCACCTGAGCCGTCTGGAAGCAAGCTGTGCAAAACTGCGTCTGA
AACTGCCGCTGCCTCGTGAAGAAGTGAAAAAAATCCTGGTTGAAATGGTGGCCAAAAGCGGTATTCGTGATGCATTTGTTGAAATTATTG
TTACCCGTGGTCTGAAAGGTGTTCGTGGTAGCCGTCCGGAAGAAATTGTTAATCGTCTGTATATGCTGGTTCAGCCGTATGTTTGGGTTA
TGGAACCGGAAGTTCAGCCGGTTGGTGGTGATGCAGTTATTGCACGTACCGTTCGTCGTGTTCCGCCTGGTAGCATTGATCCGACCGTTA
AAAATCTGCAGTGGGGTGATTTTGTGCGTGGTCTGTTTGAAGCAAGCGATCGTGGTGCAACCTATCCGTTTCTGACCGATGGTGATGCCA
ATCTGACCGAAGGTAGCGGTTTTAACATTGTTCTGGTTAAAGATGGCGTTCTGTATACACCGGATCGTGGCGTGCTGCAGGGTGTGACCC
GTAAAAGCGTTATTGATGTTGCAAATGCCAAAGGTTTTGAAGTGCGTGTTGAATATGTTCCGGTTGAAGCAGCATATCATGCCGATGAAA
TCTTTATGTGTACCACCGCAGGCGGTATTATGCCGATTCGTAGCCTGGATGGTAAACCGGTTAATGATGGTAAAGTTGGTCCGATTACCA
AAGCAATCTGGGATGGTTATTGGGAGATGCATTATGATCCGGCATATAGCTTCGAGATCAAATATCAGGTTGCAGAAGGCAAACCGCTGG
CAGGTTATCGTTTTCAAGAAAAGCTT…

In **bold**: the synthetic gene. Underlined: first and last codon of gene. Red: mutagenesis site.

Translated protein:

*MDIGINSDP***MATMDKVFAGYAARQKAMEAAGNPLSEGIAWVEGEMVPLHEARIPMLD**
EGFMRSDLTYDVPSVWDGRFFRLDDHLSRLEASCAKLRLKLPLPREEVKKILVEMVAK
SGIRDAFVEIIVTRGLKGVRGSRPEEIVNRLYMLVQPYVWVMEPEVQPVGGDAVIARTV
RRVPPGSIDPTVKNLQWGDFVRGLFEASDRGATYPFLTDGDANLTEGSGFNIVLVKDG
VLYTPDRGVLQGVTRKSVIDVANAKGFEVRVEYVPVEAAYHADEIFMCTTAGGIMPIR
SLDGKPVNDGKVGPITKAIWDGYWEMHYDPAYSFEIKYQVAEGKPLAGYRFQE*KLAAA*
ENLYFQ|GLEHHHHHH

In **bold**: the wild-type protein. In *italics*: additional amino acids from the expression vector. Underlined: TEV cleavage site. Red: G205.

4.3 AtRTA

DNA sequence:

…GAGCTCC**ATGGCCAGCATGGACAAAGTTTTTGCCGGTTATGCCGCCCGTCAAGCAATCTTAGAGAGCACGGAGACTACCAACCCATTTG
CAAAAGGTATCGCCTGGGTTGAGGGTGAATTAGTTCCATTAGCTGAGGCACGGATCCCATTATTAGATCAAGGTTTTATGCATAGCGATT
TAACGTATGATGTTCCAAGCGTTTGGGATGGTCGGTTTTTTCGGTTAGATGACCATATCACCCGGTTAGAAGCCTCGTGTACCAAATTAC
GGTTACGTTTACCATTACCACGGGATCAGGTTAAACAGATCTTAGTTGAGATGGTTGCCAAAAGCGGTATCCGTGACGCCTTCGTTGAAT
TAATCGTTACGCGTGGTTTAAAGGGCGTTCGGGGTACCCGCCCAGAAGATATCGTTAACAATTTATATATGTTTGTTCAACCATACGTGT
GGGTTATGGAGCCAGATATGCAACGTGTTGGCGGTAGCGCCGTGGTTGCACGGACGGTTCGGCGGGTTCCACCAGGTGCTATCGACCCAA
CGGTGAAAAACTTACAATGGGGCGATTTAGTTCGGGGTATGTTTGAAGCCGCAGATCGGGGTGCAACCTATCCATTTTTAACGGATGGTG
ATGCCCATTTAACGGAAGGTTCGGGTTTTAACATCGTGTTAGTTAAAGACGGTGTTTTATATACGCCGGATCGGGGTGTTTTACAGGGCG
TGACGCGGAAAAGCGTCATCAACGCAGCAGAGGCATTCGGGATCGAAGTGCGGGTTGAATTCGTTCCAGTTGAATTAGCATATCGCTGCG
ATGAAATCTTTATGTGCACGACGGCCGGTGGCATCATGCCAATCACGACGTTAGATGGTATGCCAGTCAACGGTGGTCAGATCGGTCCAA
TCACGAAAAAAATCTGGGATGGTTATTGGGCCATGCATTATGATGCAGCCTACTCGTTTGAAATCGACTATAACGAGCGTAAT**AAGCTT…

In **bold**: the synthetic gene. Underlined: first and last codon of gene. Red: mutagenesis site.

Translated protein:

*MDIGINSDPNSSS***MASMDKVFAGYAARQAILESTETTNPFAKGIAWVEGELVPLAEARIP
LLDQGFMHSDLTYDVPSVWDGRFFRLDDHITRLEASCTKLRLRLPLPRDQVKQILVEM
VAKSGIRDAFVELIVTRGLKGVRGTRPEDIVNNLYMFVQPYVWVMEPDMQRVGGSAV
VARTVRRVPPGAIDPTVKNLQWGDLVRGMFEAADRGATYPFLTDGDAHLTEGSGFNIV
LVKDGVLYTPDRGVLQGVTRKSVINAAEAFGIEVRVEFVPVELAYRCDEIFMCTTAGGI
MPITTLDGMPVNGGQIGPITKKIWDGYWAMHYDAAYSFEIDYNERN***KLAAAENLYFQ|GL
EHHHHHH*

In **bold**: the wild-type protein. In *italics*: additional amino acids from the expression vector.
Underlined: TEV cleavage site. Red: G207.

5 Enantiomeric excess chromatograms

5.1 GC-FID

o-fluoro-α-methylbenzylamine

*Ts*RTA_wild-type

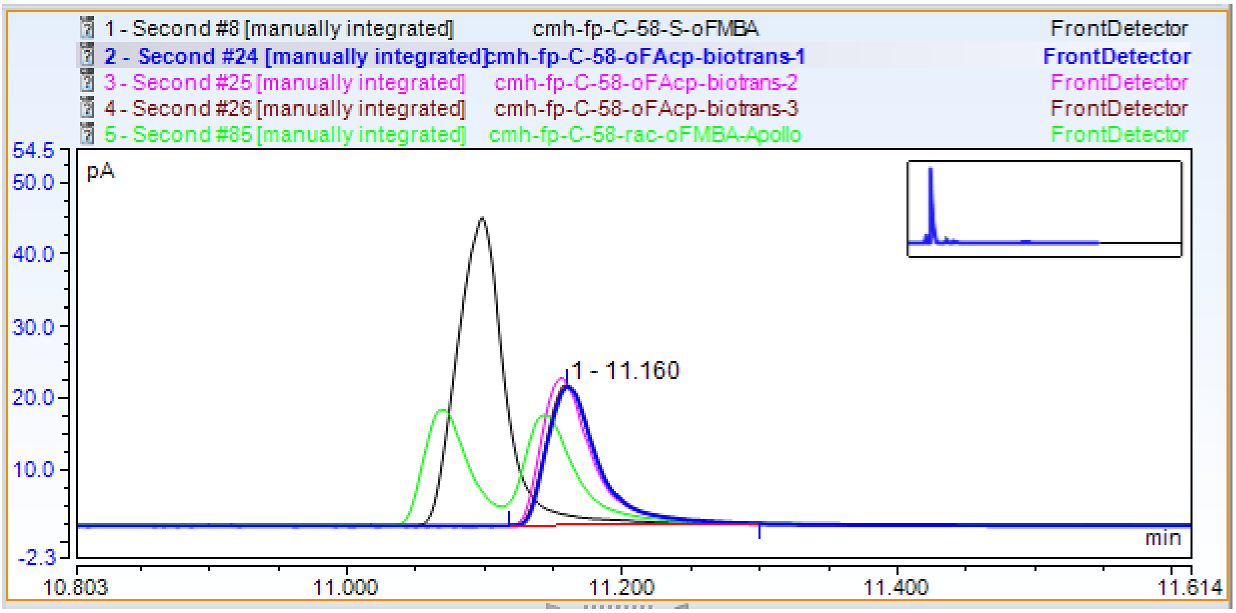

*Ts*RTA_G205C

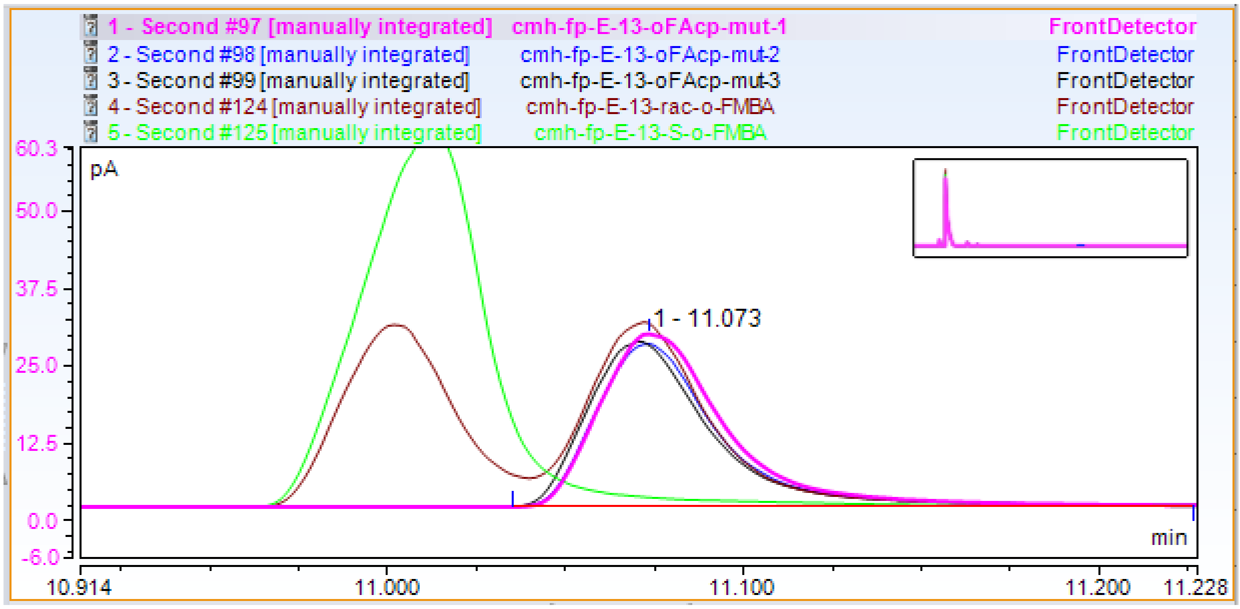

1-aminoindan

*Ts*RTA_wild-type

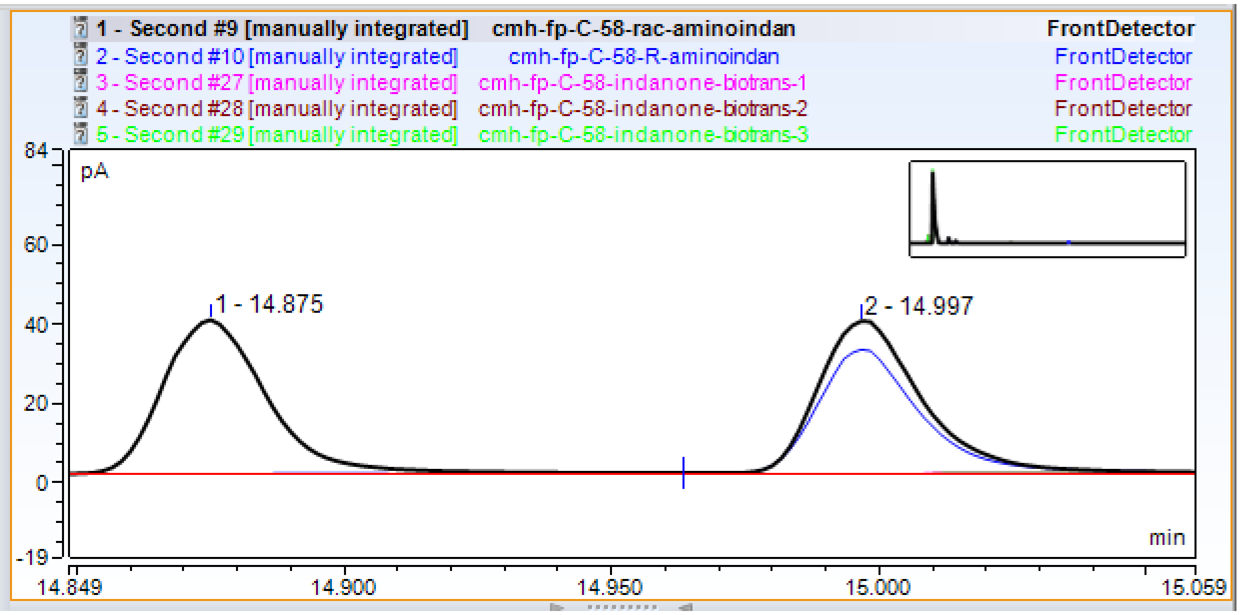

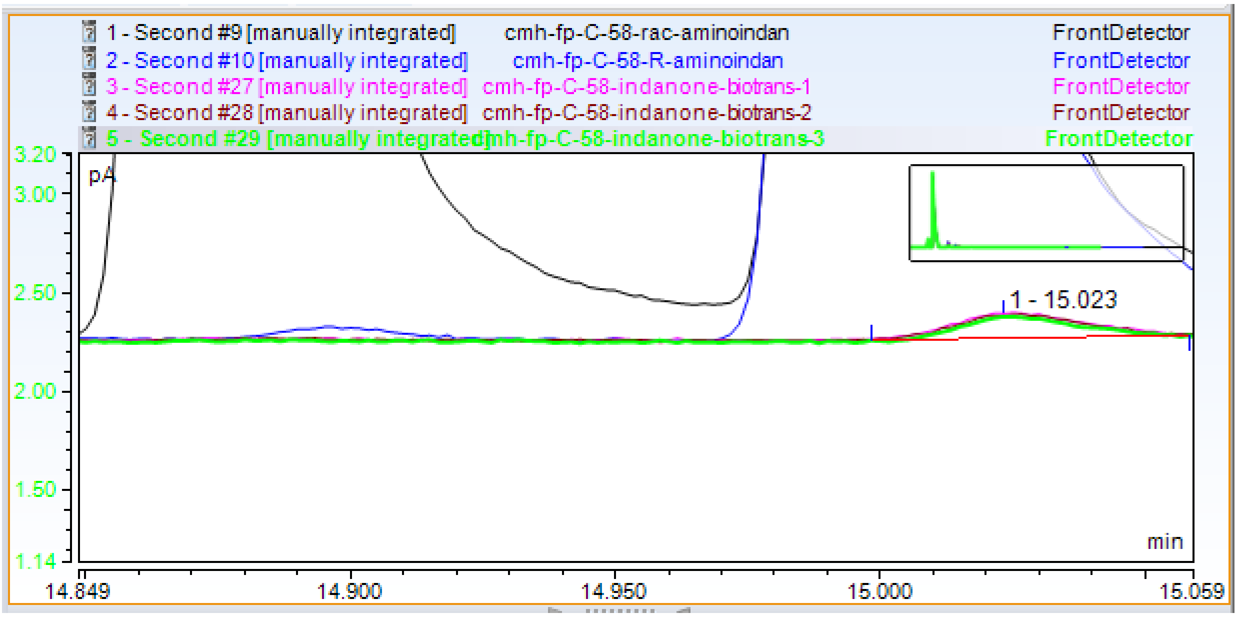

4-phenylbutan-2-amine

*Ts*RTA_wild-type

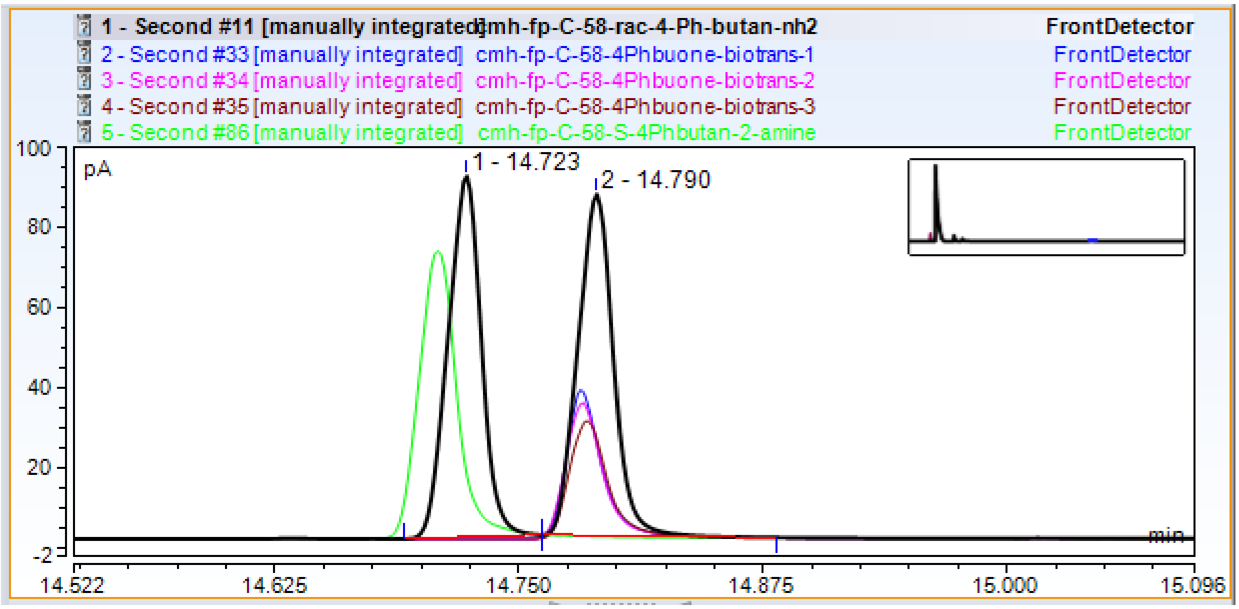

*Ts*RTA_G205C

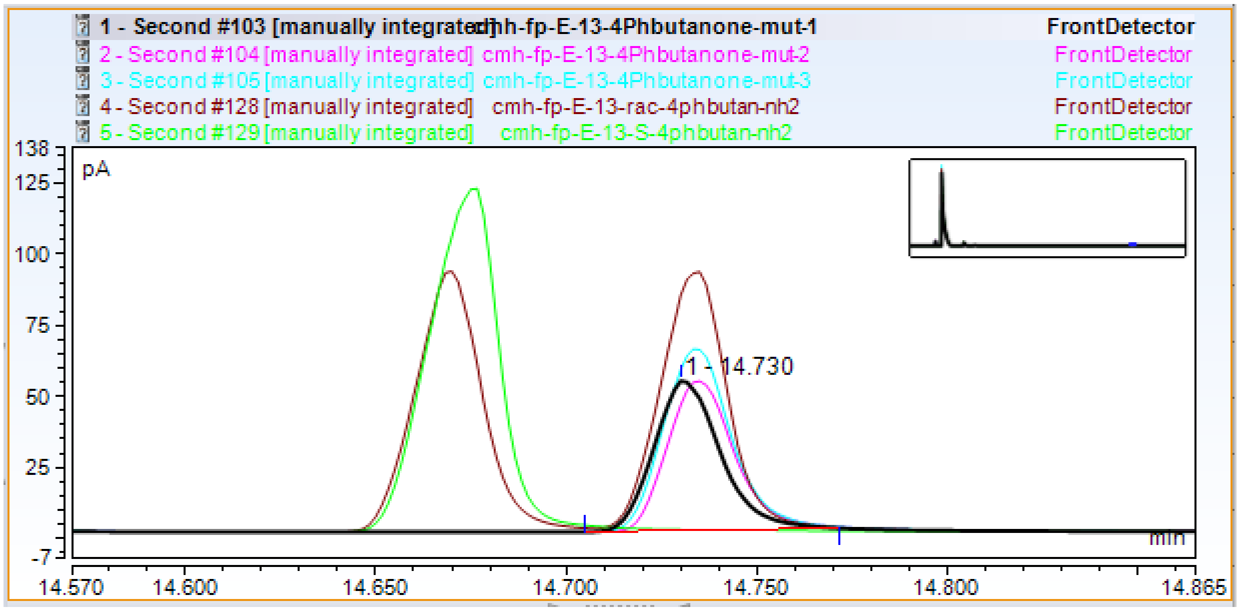

hexan-2-amine

*Ts*RTA_wild-type

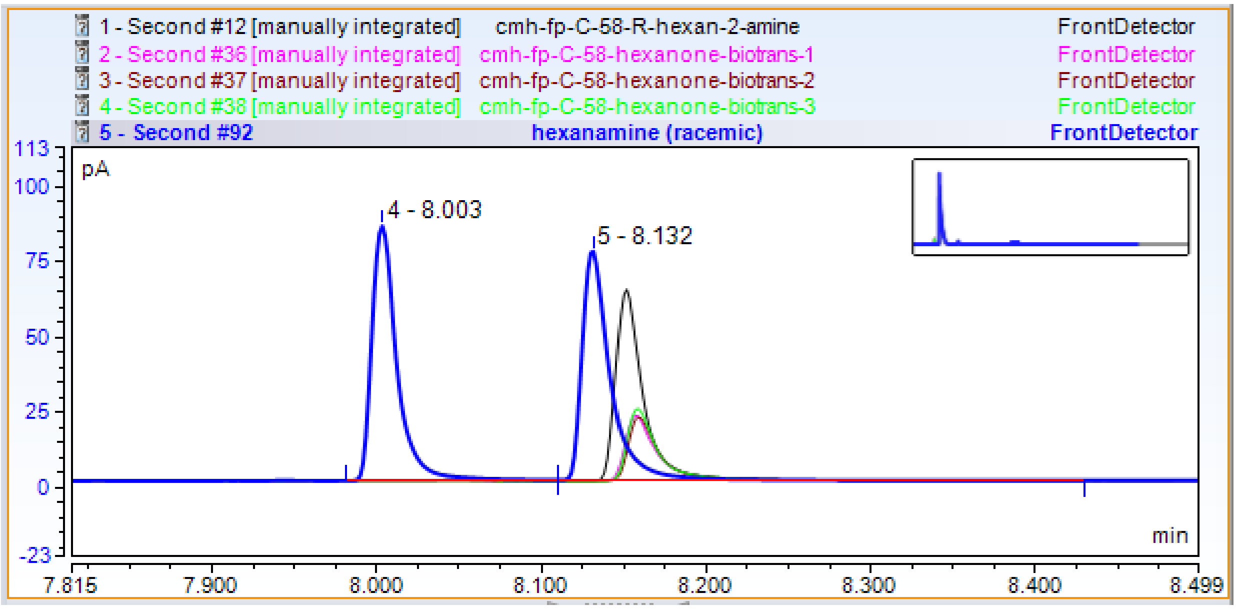

*Ts*RTA_G205C

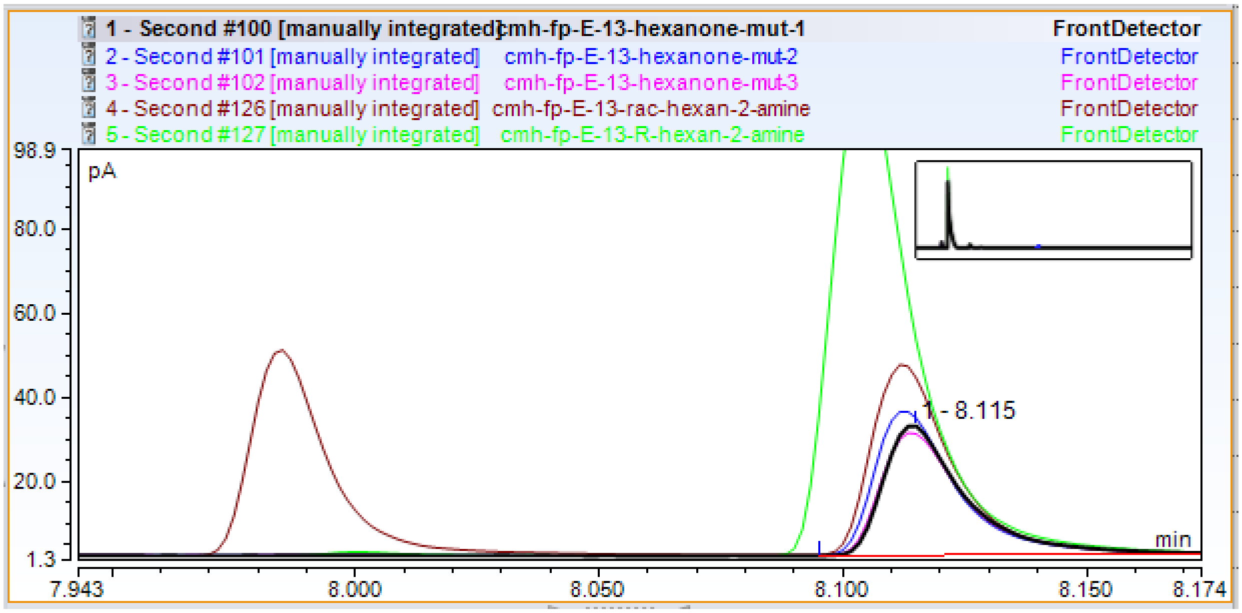

tetrahydrothiophene-3-amine

*Ts*RTA_wild-type

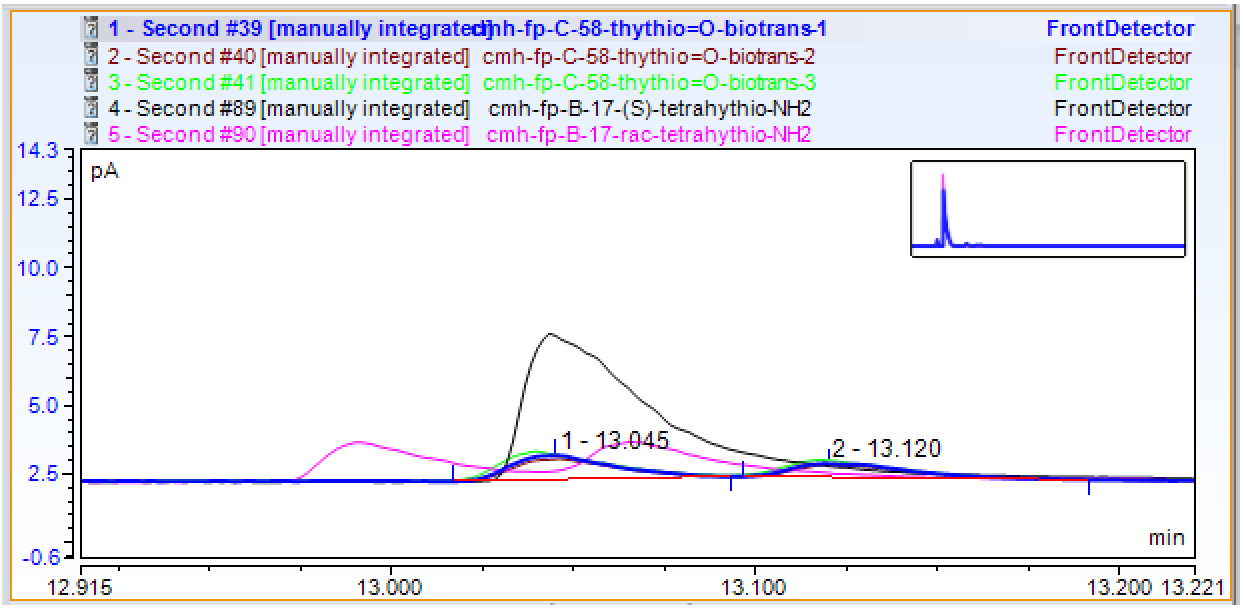

phenoxypropan-2-amine

*Ts*RTA_wild-type

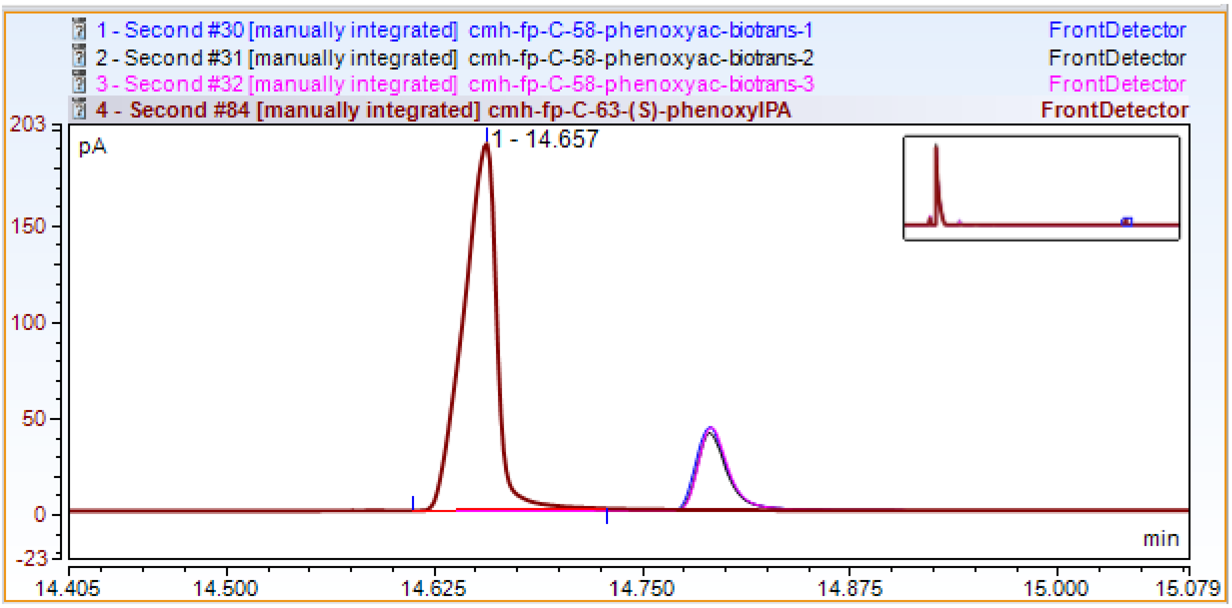

*Ts*RTA_G205C

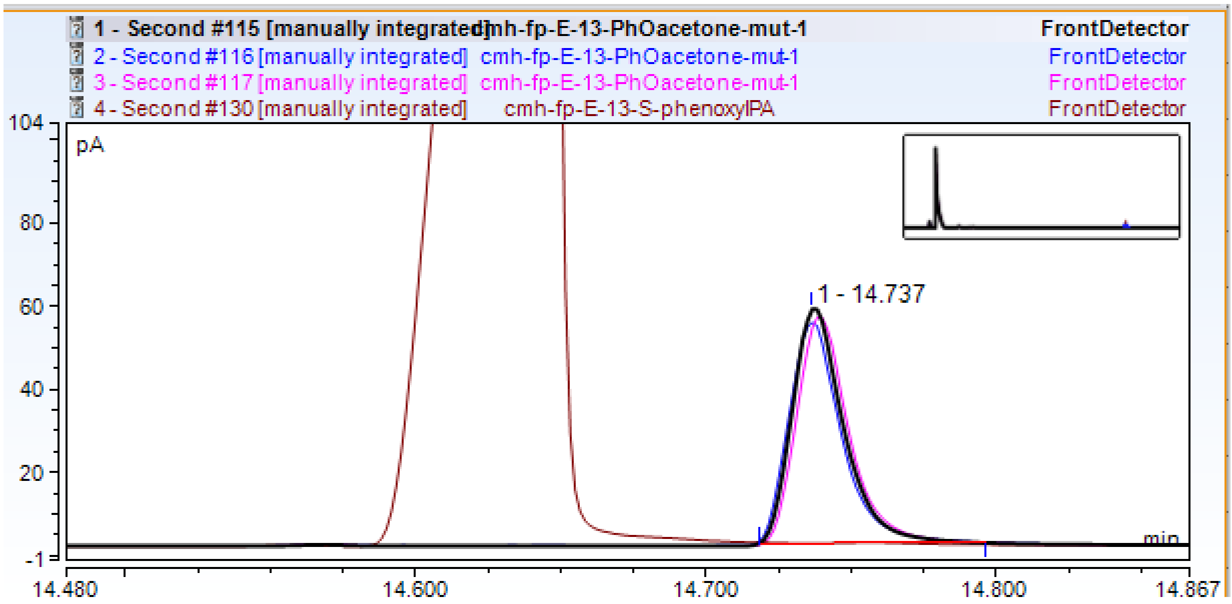

All intensification biotransformations of wild-type and G205C vs (*S*)-phenoxypropan-2-amine

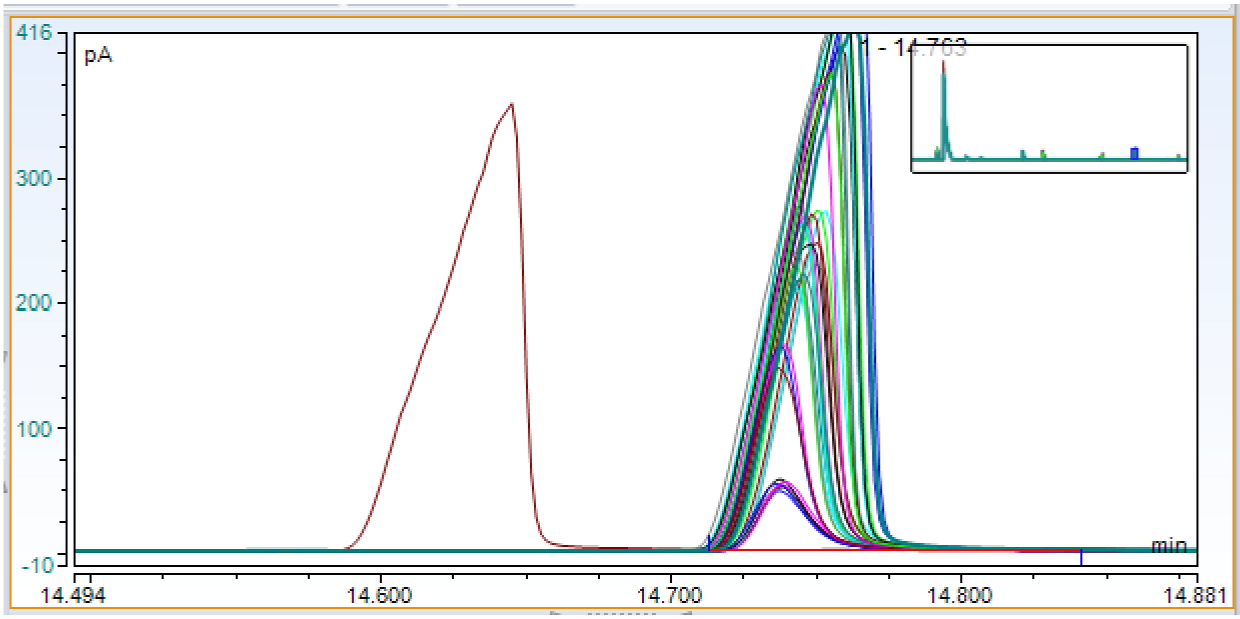

α-Ethylbenzylamine

*Ts*RTA_wild-type

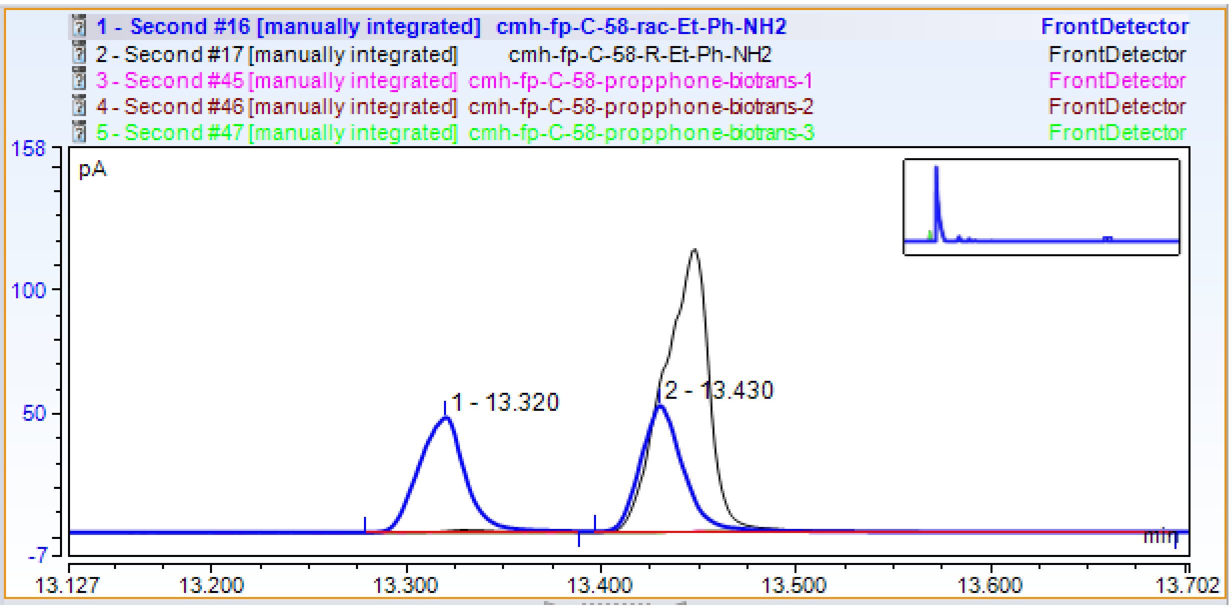

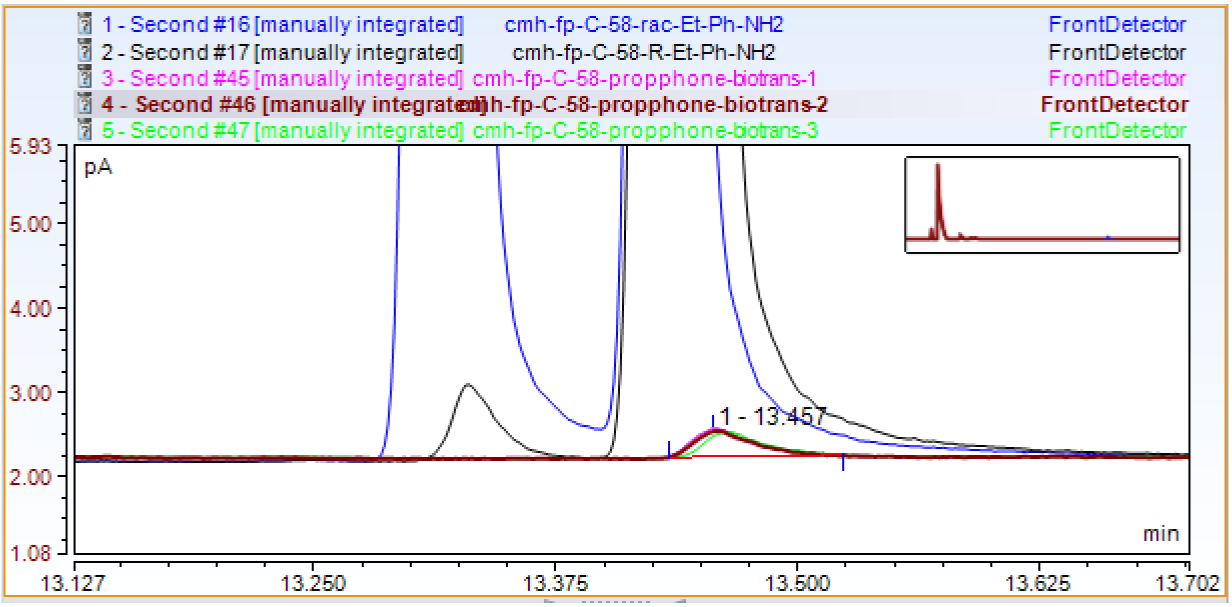

α-Methylbenzylamine

*Ts*RTA_wild-type

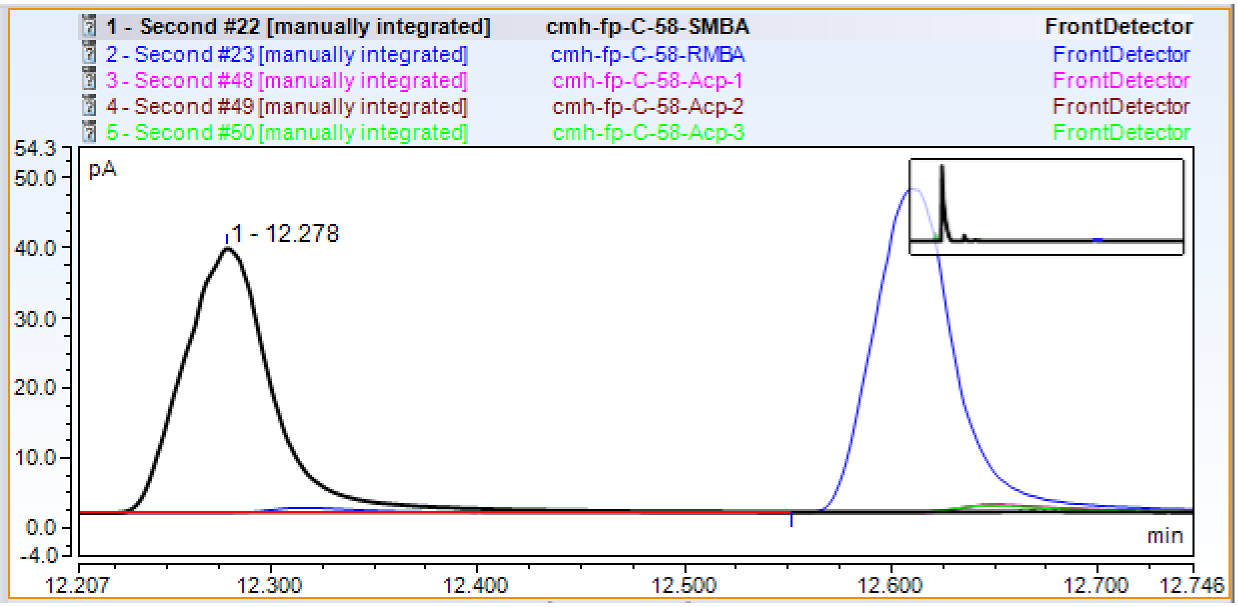

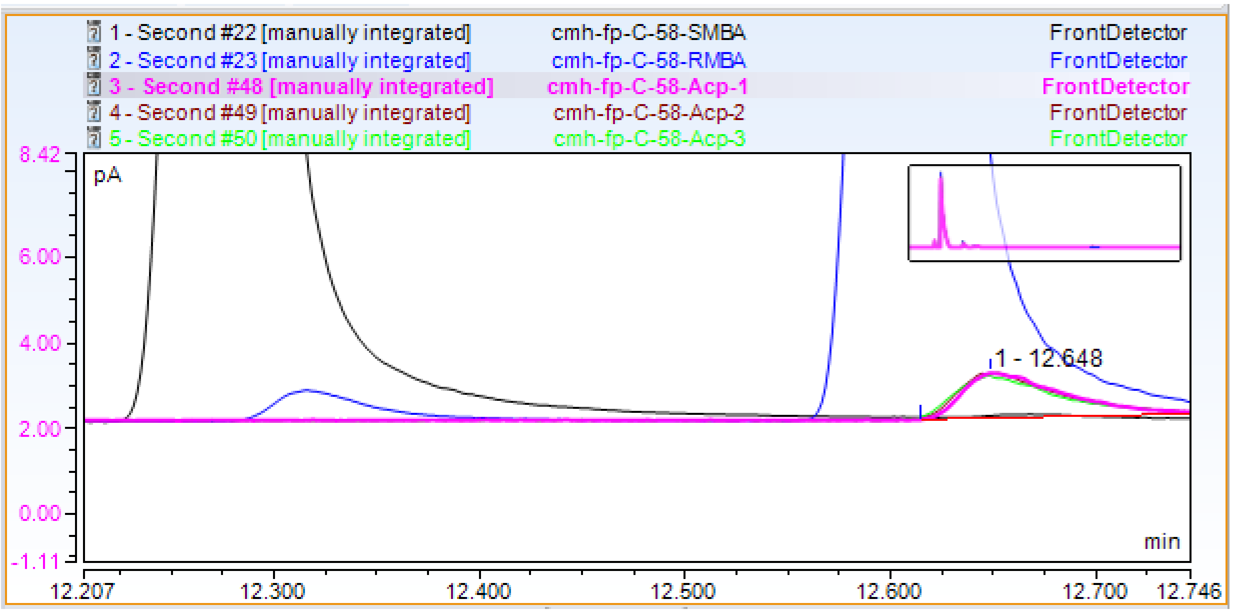

5.2 RP-HPLC

tetrahydrofuran-3-amine

*Ts*RTA_wild-type

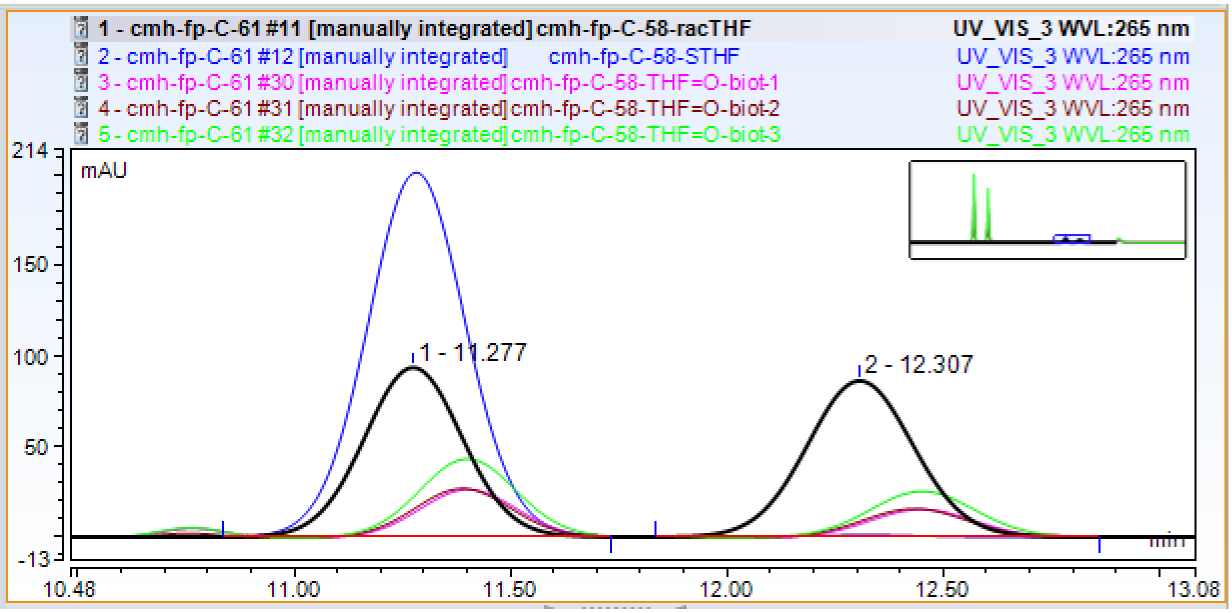

butan-2-amine

*Ts*RTA_wild-type

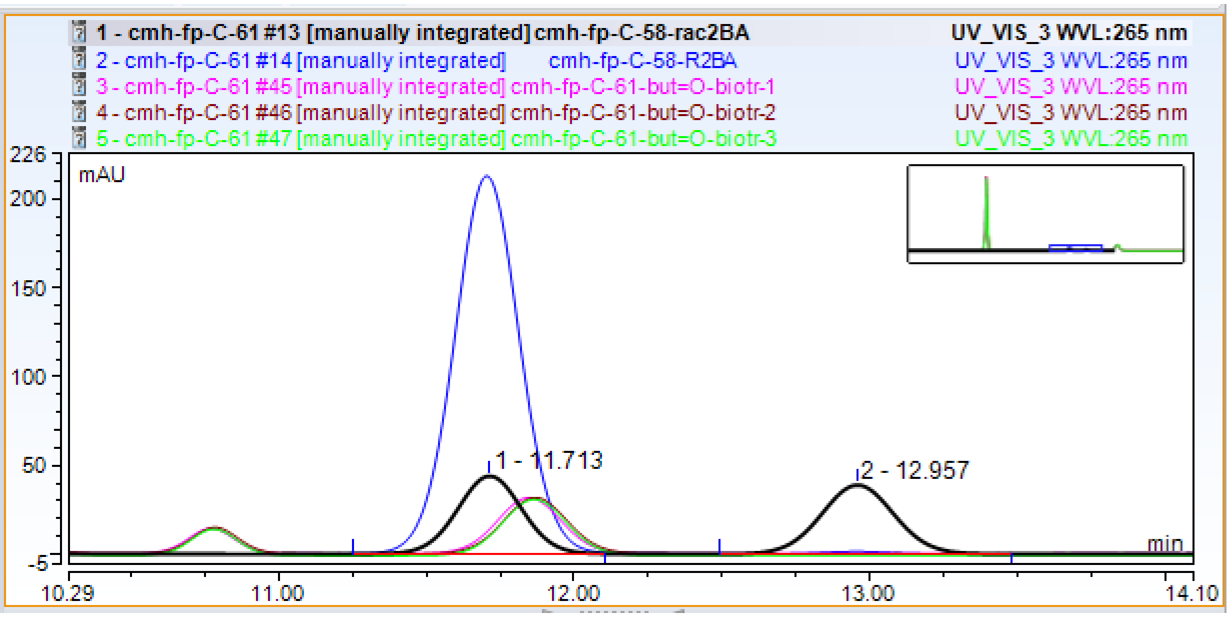

Serine

*Ts*RTA_wild-type

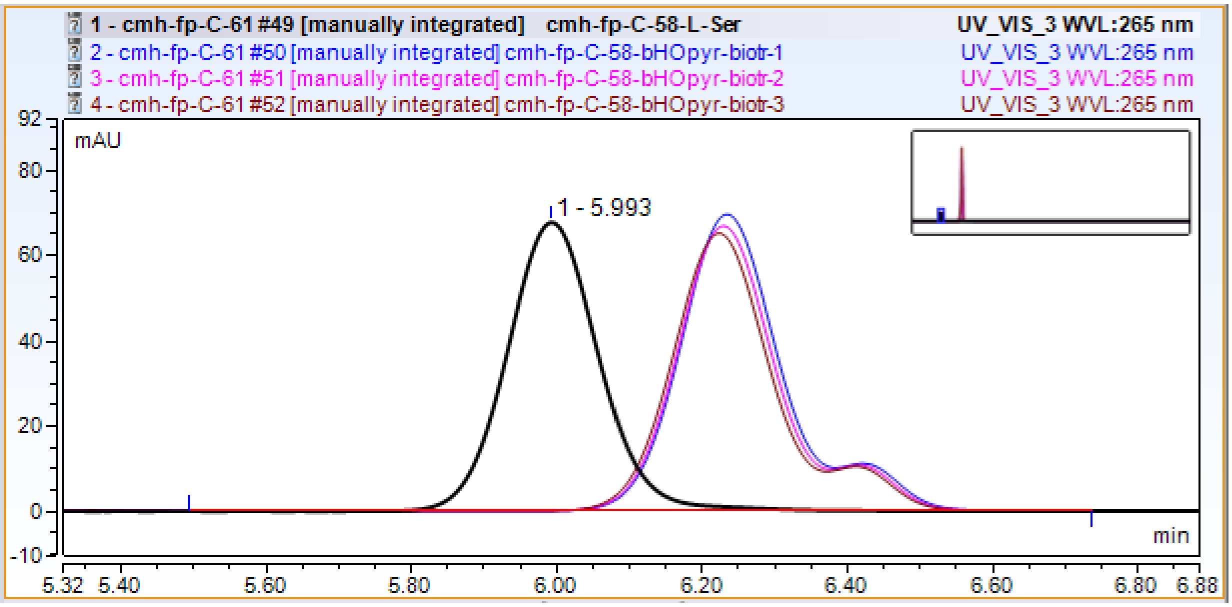

6 NMR spectra

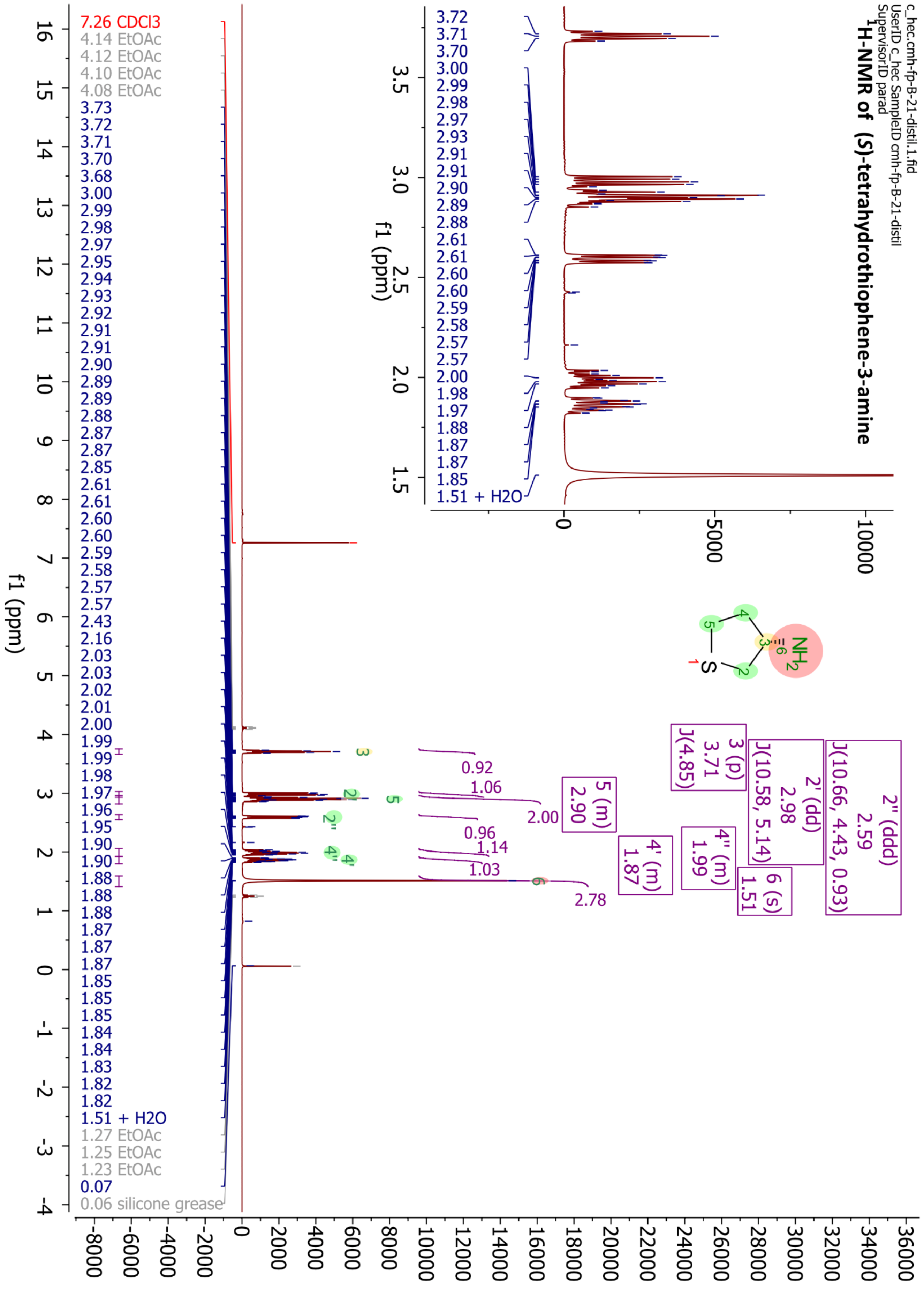
c_hec.cmh-fp-B-21-distil.1.fid
UserID c_hec SampleID cmh-fp-B-21-distil
SupervisorID parad
¹H-NMR of (S)-tetrahydrothiophene-3-amine
NH2
S
2'' (ddd) 2.59 J(10.66, 4.43, 0.93)
2' (dd) 2.98 J(10.58, 5.14)
3 (p) 3.71 J(4.85)
6 (s) 1.51
4'' (m) 1.99
4' (m) 1.87
5 (m) 2.90
0.92
1.06
2.00
0.96
1.14
1.03
2.78
7.26 CDCl3
4.14 EtOAc
4.12 EtOAc
4.10 EtOAc
4.08 EtOAc
1.51 + H2O
1.27 EtOAc
1.25 EtOAc
1.23 EtOAc
0.07
0.06 silicone grease
f1 (ppm)

c_hec.cmh-fp-B-21-distil.2.fid
UserID c_hec SampleID cmh-fp-B-21-distil SupervisorID parad
Absolute Referencing used Me4Si CDCl3, φ = 1% and Ratio of 25.145020

^{13}C-NMR of (*S*)-tetrahydrothiophene-3-amine

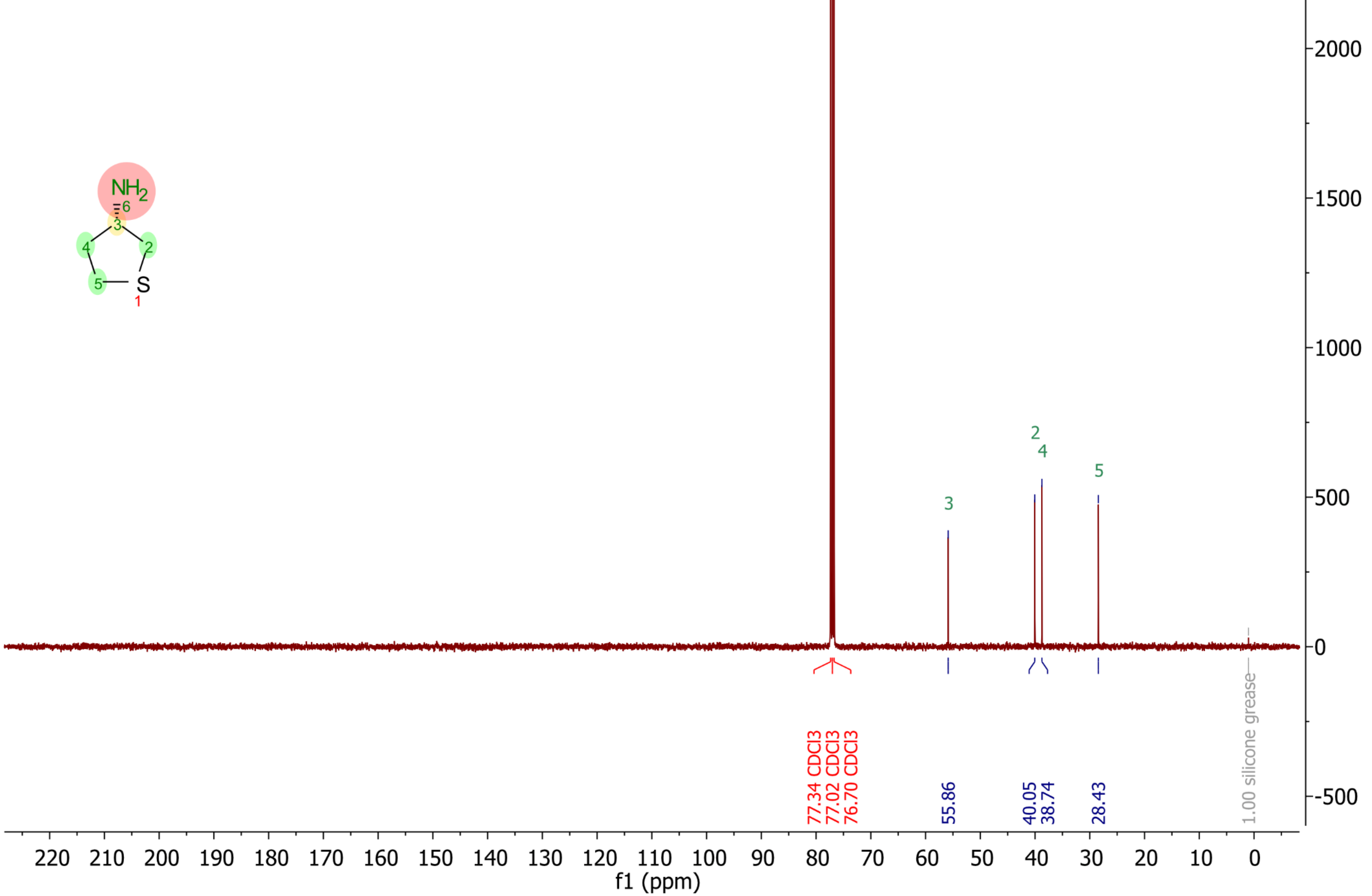

^{1}H-^{13}C-HSQC-ME of (*S*)-tetrahydrothiophene-3-amine

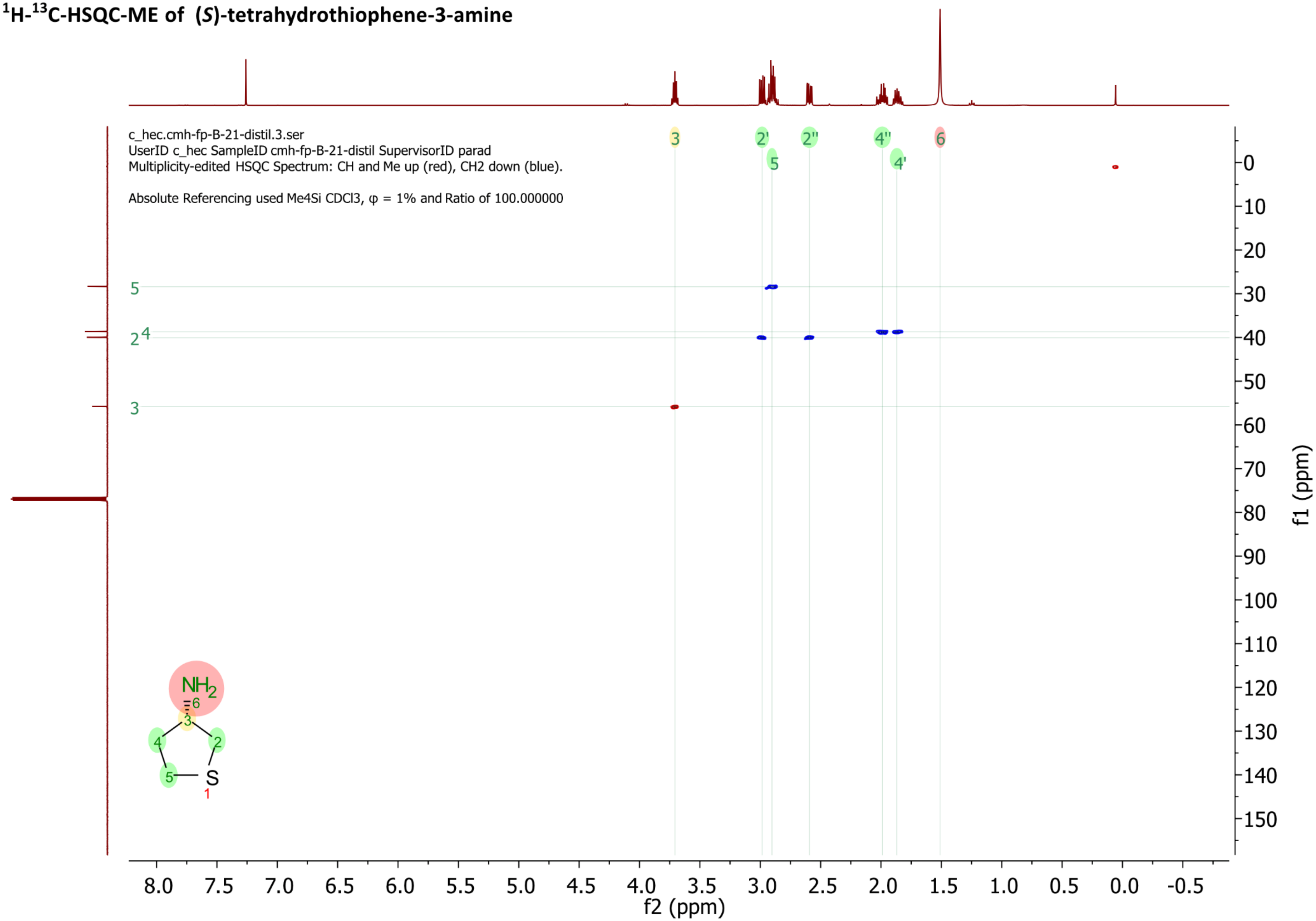

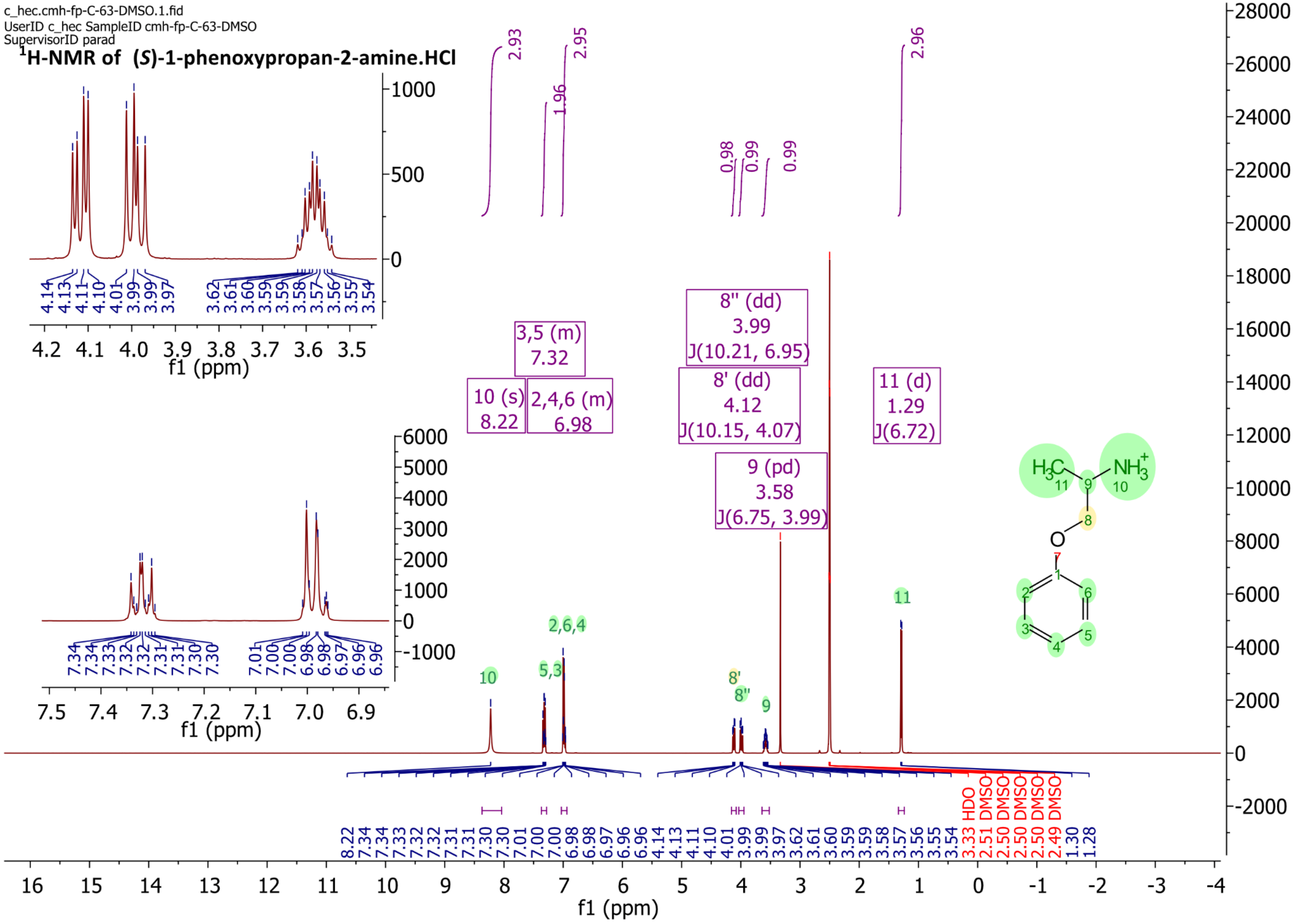

c_hec.cmh-fp-C-63-DMSO.1.fid
UserID c_hec SampleID cmh-fp-C-63-DMSO
SupervisorID parad
^{1}H-NMR of (*S*)-1-phenoxypropan-2-amine.HCl
10 (s) 8.22
3,5 (m) 7.32
2,4,6 (m) 6.98
8'' (dd) 3.99 J(10.21, 6.95)
8' (dd) 4.12 J(10.15, 4.07)
9 (pd) 3.58 J(6.75, 3.99)
11 (d) 1.29 J(6.72)
2.93
1.96
2.95
0.98
0.99
0.99
2.96
3.33 HDO
2.51 DMSO
2.50 DMSO
2.50 DMSO
2.50 DMSO
2.49 DMSO
f1 (ppm)

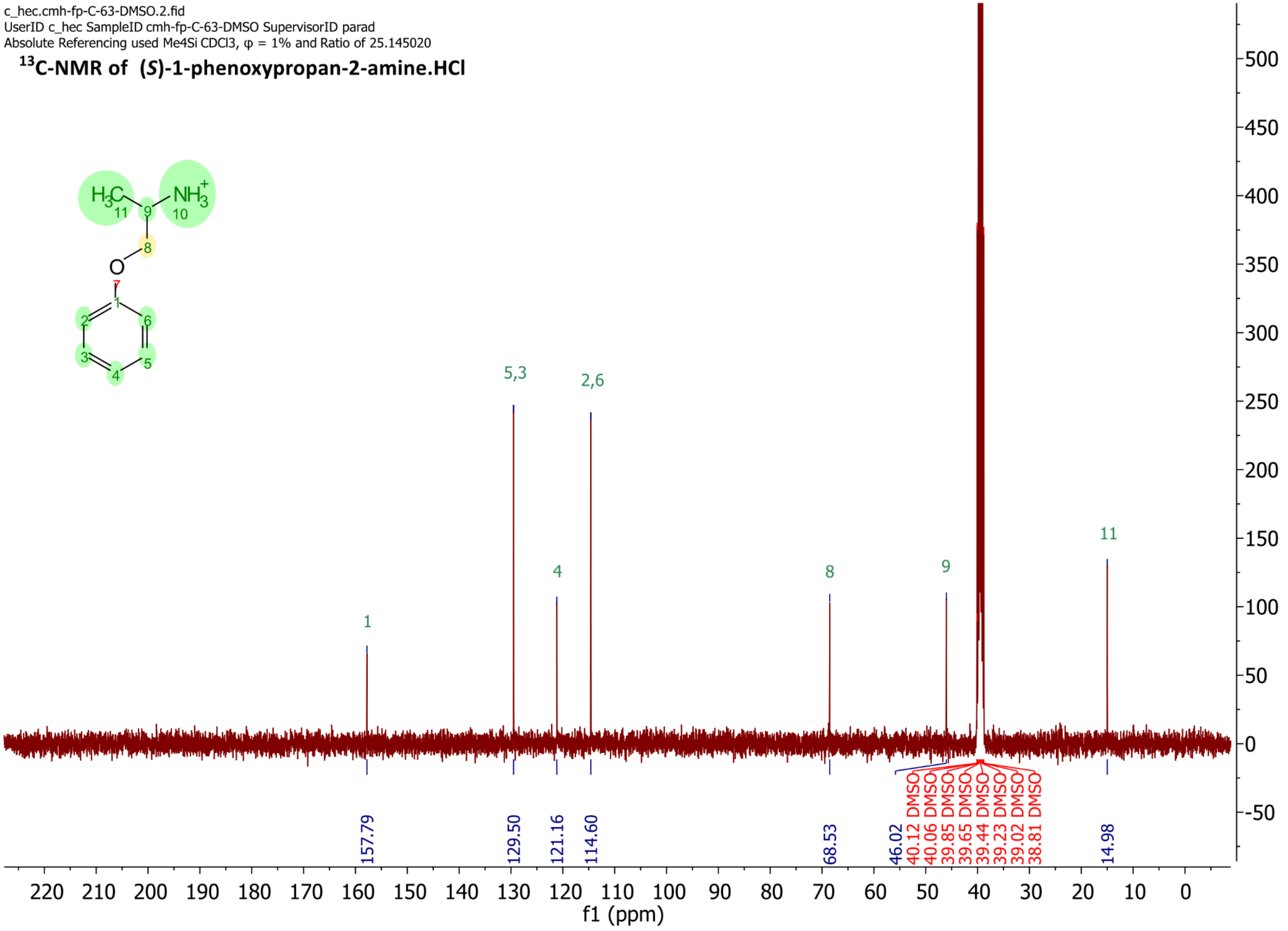

c_hec.cmh-fp-C-63-DMSO.2.fid
UserID c_hec SampleID cmh-fp-C-63-DMSO SupervisorID parad
Absolute Referencing used Me4Si CDCl3, φ = 1% and Ratio of 25.145020
13C-NMR of (S)-1-phenoxypropan-2-amine.HCl
1
5,3
4
2,6
8
9
11
157.79
129.50
121.16
114.60
68.53
46.02
40.12 DMSO
40.06 DMSO
39.85 DMSO
39.65 DMSO
39.44 DMSO
39.23 DMSO
39.02 DMSO
38.81 DMSO
14.98
f1 (ppm)

^{1}H-^{13}C-HSQC-ME of (*S*)-1-phenoxypropan-2-amine.HCl

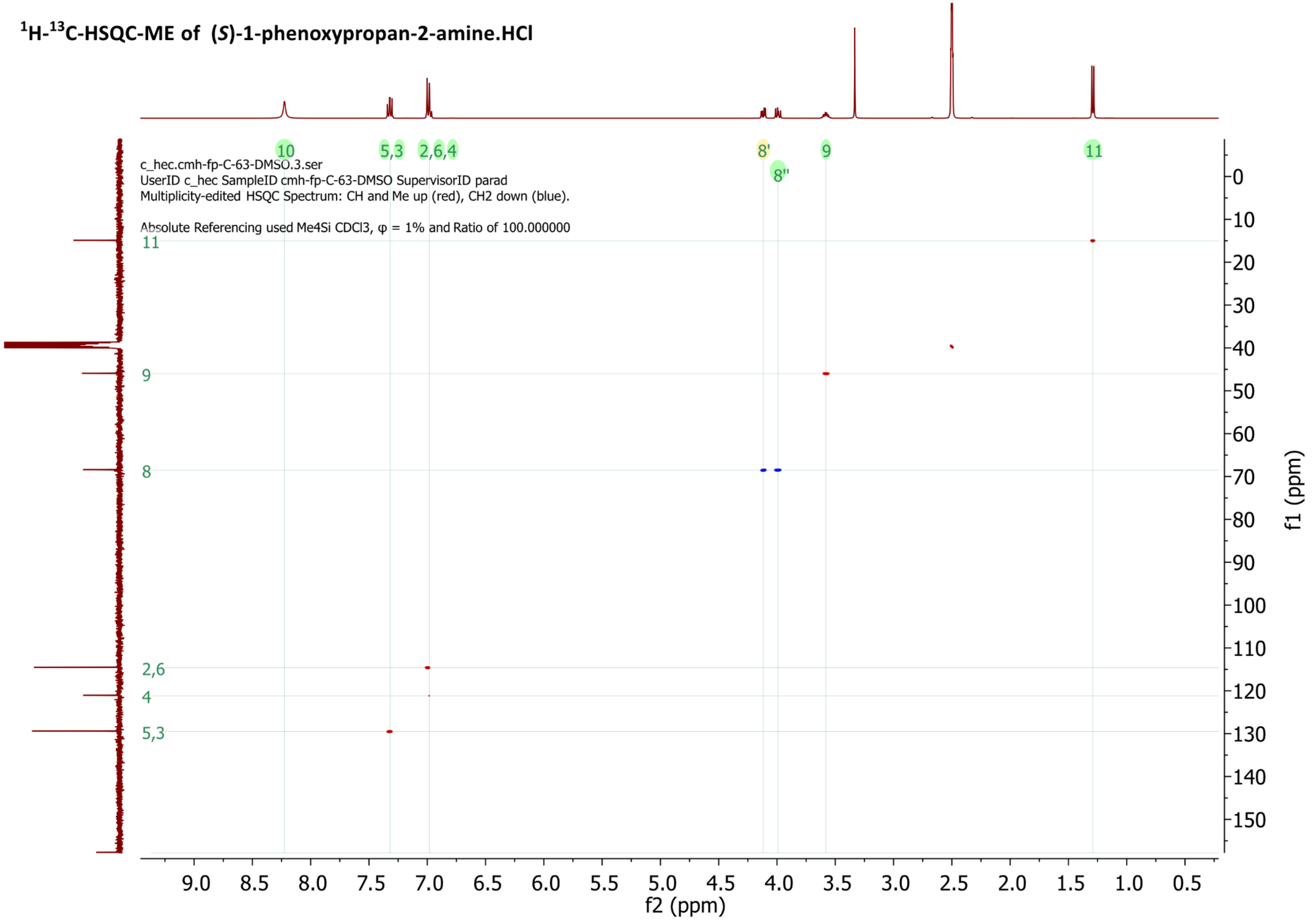

Supplement: Supplementary file 1 [file Data_Sheet_1.pdf]
